# In Vivo Regulation of the Zebrafish Endoderm Progenitor Niche by T-Box Transcription Factors

**DOI:** 10.1016/j.celrep.2017.06.011

**Published:** 2017-06-27

**Authors:** Andrew C. Nelson, Stephen J. Cutty, Saule N. Gasiunas, Isabella Deplae, Derek L. Stemple, Fiona C. Wardle

**Affiliations:** 1 Randall Division of Cell and Molecular Biophysics, King’s College London, London SE1 1UL, UK; 2 Sir William Dunn School of Pathology, University of Oxford, Oxford OX1 3RE, UK; 3 Wellcome Trust Sanger Institute, Hinxton, Cambridge CB10 1SA, UK; 4 School of Life Sciences, University of Warwick, Coventry CV4 7AL, UK

**Keywords:** T-box, endoderm, transcription, ChIP-seq, redundancy

## Abstract

T-box transcription factors T/Brachyury homolog A (Ta) and Tbx16 are essential for correct mesoderm development in zebrafish. The downstream transcriptional networks guiding their functional activities are poorly understood. Additionally, important contributions elsewhere are likely masked due to redundancy. Here, we exploit functional genomic strategies to identify Ta and Tbx16 targets in early embryogenesis. Surprisingly, we discovered they not only activate mesodermal gene expression but also redundantly regulate key endodermal determinants, leading to substantial loss of endoderm in double mutants. To further explore the gene regulatory networks (GRNs) governing endoderm formation, we identified targets of Ta/Tbx16-regulated homeodomain transcription factor Mixl1, which is absolutely required in zebrafish for endoderm formation. Interestingly, we find many endodermal determinants coordinately regulated through common genomic occupancy by Mixl1, Eomesa, Smad2, Nanog, Mxtx2, and Pou5f3. Collectively, these findings augment the endoderm GRN and reveal a panel of target genes underlying the Ta, Tbx16, and Mixl1 mutant phenotypes.

## Highlights

• Ta and Tbx16 redundantly regulate genes through common *cis-*regulatory modules
• Ta and Tbx16 control expression of *mixl1* and other key endodermal regulators
• Mixl1 occupies endodermal CRMs with Smad2, Eomesa, Nanog, Mxtx2, and Pou5f3
• *Ta* and *tbx16* double mutants fail to correctly form liver, pancreas, and gut tube

## Graphical Abstract

**Figure:**
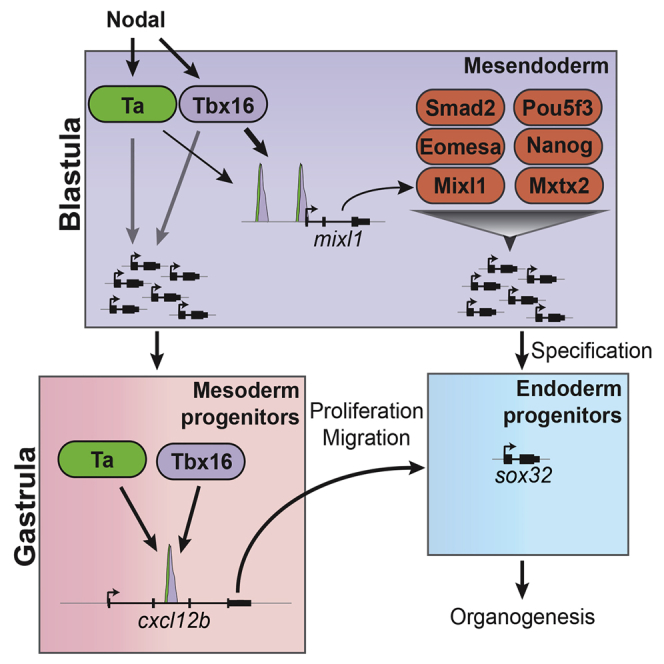

## Teaser

Endoderm contributes to the respiratory and gastrointestinal tracts and all associated organs. How transcription factors control endodermal progenitor specification and expansion is not completely understood. Here, Nelson et al. identify a key redundant requirement for T-box factors Ta and Tbx16 in zebrafish endoderm formation and explore the downstream transcriptional programs.

## Introduction

The primary germ layers of the vertebrate embryo—endoderm, mesoderm, and ectoderm—are specified early in development. Endoderm derivatives contribute to liver, pancreas, gut tube, and respiratory tract, whereas mesoderm gives rise to muscle, connective tissues, and blood. The transforming growth factor β (TGF-β) family growth factor Nodal is required for formation of bipotential precursors of mesoderm and endoderm—the mesendoderm (Schier, 2009). On pathway activation, its downstream effectors, transcription factors (TFs) Smad2/3, translocate into the nucleus and interact with other TFs, such as the T-box TF Eomes, to activate expression of mesendodermal target genes.

T-box TFs play key roles in mesoderm and endoderm formation. For example, in mouse, Eomesodermin (Eomes) is required for definitive endoderm formation (Arnold et al., 2008), whereas zebrafish Eomes homolog A (Eomesa) regulates early endoderm marker expression (Du et al., 2012). T is required for normal mesoderm formation, with notochord and posterior somites failing to differentiate in mutant mice (Dobrovolskaïa-Zavadskaïa, 1927). In zebrafish, Ta is also required for notochord formation and it acts synergistically with its paralog, Tb, in posterior somite formation (Halpern et al., 1993, Martin and Kimelman, 2008, Schulte-Merker et al., 1994). Another T-box TF, Tbx16, plays a key role in zebrafish mesoderm formation though directing migration of mesodermal progenitors during gastrulation (Ho and Kane, 1990). Both Ta and Tbx16 regulate fibroblast growth factor (FGF) and Wnt signaling to control intermediate mesoderm formation and somitogenesis (Kimelman, 2016, Mueller et al., 2010, Warga et al., 2013) and have independent and combinatorial roles in establishing left-right asymmetry (Amack et al., 2007). Indeed, T-box TFs often share partially overlapping functions. For example, in *Xenopus* T, Eomes and VegT (ortholog of Tbx16; Griffin et al., 1998) redundantly regulate neuromesodermal bipotency (Gentsch et al., 2013).

This study focuses on transcriptional networks directed by Ta and Tbx16 in early zebrafish development. We characterized their DNA-binding activities and target gene expression profiles during gastrulation. We discovered that Ta/Tbx16 genomic binding substantially overlaps and provide evidence that use of common *cis*-regulatory modules (CRMs) accounts for their functional redundancy (Garnett et al., 2009). Here, we describe a profound loss of endoderm in *ta/tbx16* double mutants and present findings demonstrating that Ta/Tbx16 directly regulate the cell-intrinsic endodermal regulator Mixl1 (Kikuchi et al., 2000), as well as extrinsic regulators of endoderm proliferation, the Cxcr4a ligands Cxcl12a/b (Mizoguchi et al., 2008, Stückemann et al., 2012).

To understand how transcriptional programs downstream of Ta and Tbx16 control endoderm formation, we assessed Mixl1 genomic binding during endoderm specification, revealing direct regulation of many key endoderm-intrinsic factors via CRM occupancy with Smad2 and Eomesa. Moreover, we found Mixl1 binds common CRMs with key endodermal determinants Nanog, Mxtx2, and Pou5f3 (Leichsenring et al., 2013, Lunde et al., 2004, Reim et al., 2004, Xu et al., 2012). Collectively, our data refine the transcriptional hierarchy underlying endoderm formation in zebrafish and strongly suggest these TFs act combinatorially to regulate target gene expression.

## Results

### Genome-wide ChIP-Seq Analysis of Ta and Tbx16 Binding in Zebrafish Gastrulae

**Figure 1:**
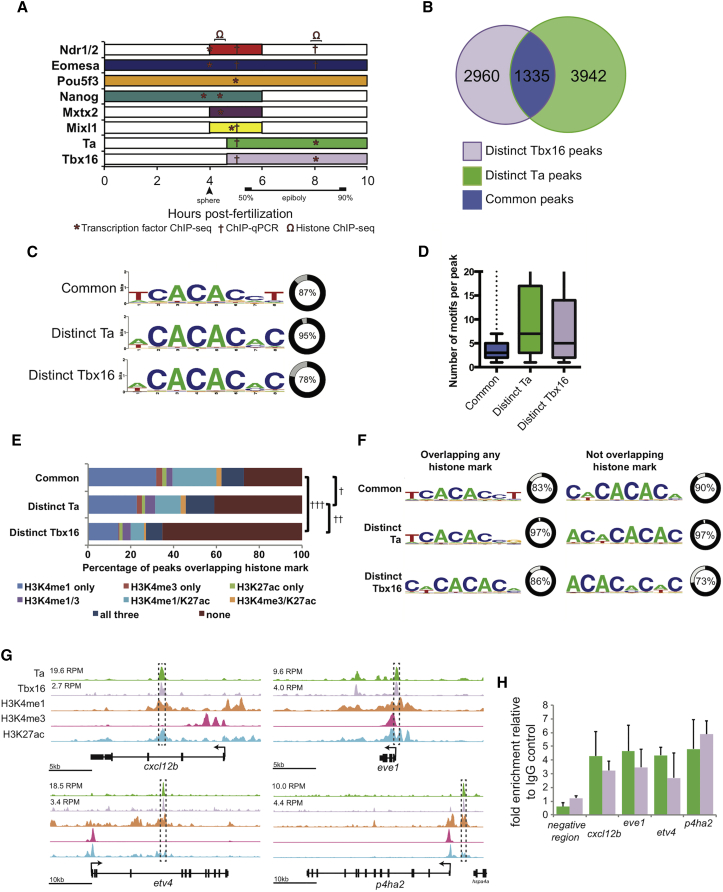
Genome-wide Analysis of Ta and Tbx16 Binding Sites (A) Summary of the expression of the endodermal regulators (or their upstream activator) for which ChIP data are presented. Bars indicate the temporal expression window of factors at the margin, color coded per factor as in subsequent figures. Datasets indicated are ChIP-seq: Smad2 (regulated by Ndr1/2) and Eomesa at 3.3–4 hpf; Nanog and Mxtx2 at 3.3 and 4.3 hpf; Pou5f3 at 5 hpf; Mixl1 at 4.7–5.3 hpf; Ta and Tbx16 at 8–8.5 hpf; and histones at 8.25 hpf. ChIP-qPCR are Smad2, Eomesa, Mixl1, Ta, and Tbx16 at 5.3 hpf and Ta and Tbx16 at 8–8.5 hpf. (B) Overlap of Ta and Tbx16 ChIP-seq peaks at 75%–85% epiboly (8–8.5 hpf). (C) Closest match to the consensus T-box binding site identified within each peak class. Percentage of peaks containing such a sequence is indicated. (D) Occurrences of motifs indicated in (C) within each peak of each class. Boxplots intervals are 10^th^, 25^th^, median, 75^th^, and 90^th^ percentiles. (E) Percentage of peaks in each class overlapping histone marks. †p = 3 × 10^−19^; ††p = 4 × 10^−89^; †††p = 9 × 10^−119^, chi-square test. See also Figure S1. (F) Closest match to the canonical T-box binding site identified within each class of peak overlapping histone marks with percentage of peaks containing such sequences indicated. (G) Stage-matched Ta, Tbx16, H3K4me1, H3K4me3, and H3K27ac and ChIP-seq at the various genomic loci. Peak heights in reads per million (RPM) are indicated. Boxed regions indicate regions used for ChIP-qPCR validation. (H) ChIP-qPCR validation of regions indicated in (G). Data are represented as mean ± SEM.

To study the roles of Ta, Tbx16, and other TFs, we assessed DNA binding, histone modification, and Ta/Tbx16-dependent target gene expression profiles between zygotic genome activation and the end of gastrulation. Figure 1A shows time points for individual TF datasets and the temporal expression of these TFs at the margin (mesodermal and endodermal cells).

Ta and Tbx16 chromatin immunoprecipitation sequencing (ChIP-seq) at mid-gastrulation (75%–85% epiboly; 8–8.5 hr post-fertilization [hpf]) identified a similar number of binding events (ChIP-seq peaks; Data S1) for each TF. Of these, ∼25%–30% overlap (Figure 1B; Data S1), which we designate “common” peaks, whereas peaks unique to each TF we designate “distinct”.

Previous studies demonstrated Ta and Tbx16 bind the T-box consensus sequence TCACACCT (Garnett et al., 2009, Kispert and Hermann, 1993, Morley et al., 2009); however, T-box TFs also bind AC-rich sequences at lower affinity (Evans et al., 2012). De novo motif analysis revealed 85% of common peaks contain close matches to the consensus T-box site, whereas distinct peaks are most enriched for AC-rich sequences (Figure 1C). Interestingly, common peaks contain few consensus T-box sites, whereas the distinct peaks contain numerous AC-rich sites (Figure 1D).

To determine whether there are differences in functionality between peak subsets, we compared our data with published histone ChIP-seq indicative of putative promoters (H3K4me3), putative enhancers (H3K4me1), and active enhancers (H3K27ac) (Bogdanovic et al., 2012). This genome-wide analysis revealed common peaks were significantly more likely to overlap these histone marks than distinct peaks, whereas distinct Ta peaks were more correlated with histone marks than distinct Tbx16 peaks (Figure 1E). Interestingly, whereas common peaks at histone marks are most enriched for the consensus T-box, common peaks lacking functional marks are most enriched for the AC-rich sequences (Figure 1F).

Putative target genes were then annotated (nearest transcription start site [TSS] ± 100 kb from each peak, though the majority are markedly closer; Figure S1). These, together with Ta and Tbx16 binding coordinates and associations with histone marks, are shown in Data S1. Among target genes with common Ta/Tbx16 peaks at functional chromatin are FGF target gene *etv4* (Roehl and Nüsslein-Volhard, 2001), mesodermally expressed endodermal regulator *cxcl12b* (Mizoguchi et al., 2008, Nair and Schilling, 2008, Stückemann et al., 2012), mesodermal progenitor regulator *eve1* (Seebald and Szeto, 2011), and migration-associated marker *ph4a2* (Chang et al., 2011; Figures 1G and 1H), all of which play roles in key Ta and Tbx16 activities.

### Expression Profiling of Ta and Tbx16 Target Genes

**Figure 2:**
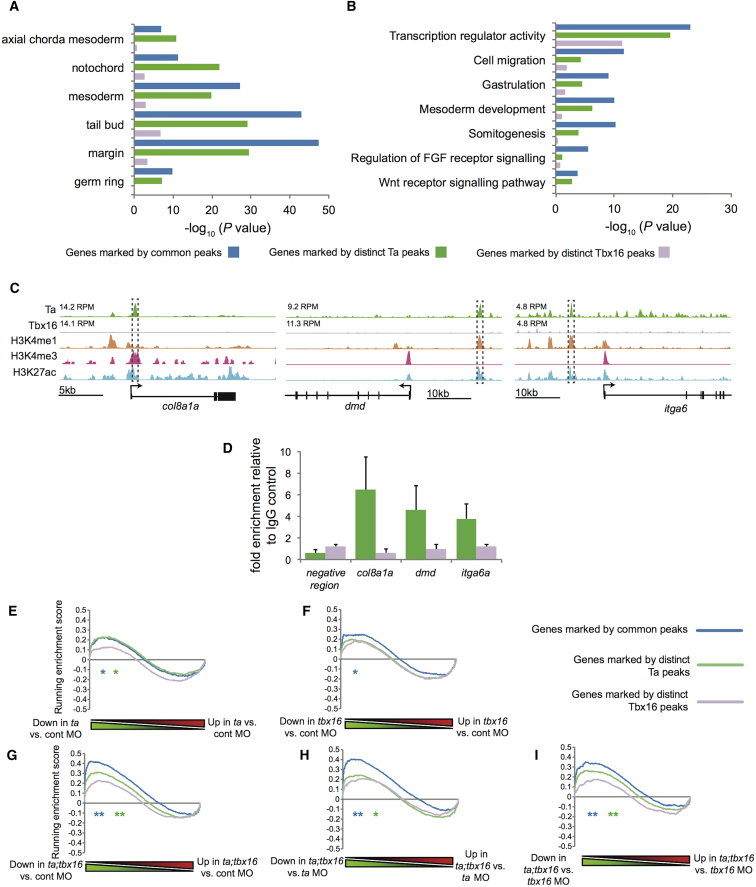
Ta and Tbx16 Show Cell-Type-Specific Binding Profiles and Redundantly Regulate Genes Showing Common Occupancy of Both Factors (A) Enrichment for target genes with distinct Ta, Tbx16, or common binding (as indicated in Figure 1B) expressed within cell types where *ta* and/or *tbx16* are expressed, as defined by the ZFIN database (http://www.zfin.org; Howe et al., 2013). Blue, common peaks; green, distinct Ta peaks; purple, distinct Tbx16 peaks. (B) Bar graph showing enrichment for Gene Ontology terms associated with target genes with distinct or common binding (as indicated in Figure 1B). (C) Stage-matched Ta, Tbx16, H3K4me1, H3K4me3, and H3K27ac and ChIP-seq profiles. Peak heights in RPM are indicated. Boxed regions indicate peaks used for ChIP-qPCR validation. (D) Ta (green) and Tbx16 (mauve) ChIP-qPCR validation of regions indicated in (C). Data are represented as mean ± SEM. (E–I) GSEA enrichment plots for comparison of target genes with distinct or common binding (as indicated in Figure 1B) with (E) *ta* KD relative to control; (F) *tbx16* KD relative to control; (G) *ta/tbx16* double KD relative to control; (H) *ta/tbx16* double KD relative to *ta* KD; (I) *ta/tbx16* double KD relative to *tbx16* KD. ^∗^Family-wise error rate (FWER) p ≤ 3 × 10^−2^; ^∗∗^FWER p ≤ 5 × 10^−4^.

Expression of target genes with common peaks was significantly enriched in tissues co-expressing Ta and Tbx16, such as the margin and tailbud (Figure 2A; Data S2). Similarly, gene ontology (GO) term analysis demonstrates enrichment for functions common to both TFs (Figure 2B; Data S2). Interestingly, genes with distinct Ta, but not Tbx16 peaks, show enriched expression in the axial chorda mesoderm and notochord, where *ta*, but not *tbx16*, is expressed (Figures 2A and 2C). ChIP-qPCR validation confirms Ta-specific binding at axial chorda mesoderm genes *col8a1a*, *dmd*, and *itga6* (Figure 2D). Thus, consistent with enrichment of distinct Ta peaks at functional chromatin marks (Figure 1E), these Ta-binding events seem to be cell type specific.

To further investigate functional redundancy, we examined microarray data for single or double knockdown (KD) of Ta and Tbx16 by validated morpholino (MO) injection, at the same developmental stage as our ChIP-seq data (Garnett et al., 2009). As judged by gene set enrichment analysis (GSEA), Ta/Tbx16 occupancy is not highly correlated with changes in target gene expression upon loss of either TF alone (Figures 2E and 2F). However, common binding is significantly correlated with changes on combinatorial loss of both TFs (Figures 2G–2I). This suggests that, whereas changes in target gene expression occur on loss of each individual TF, leading to the known mutant phenotypes, loss of both TFs leads to greater reduction of a subset of genes with Ta/Tbx16 binding at the same CRMs. These data extend earlier conclusions (Garnett et al., 2009), suggesting that target gene expression is controlled through common rather than distinct CRMs.

### Ta and Tbx16, Together with Eomesa and Smad2, Cooperatively Control Expression of Key Endodermal Regulators

**Figure 3:**
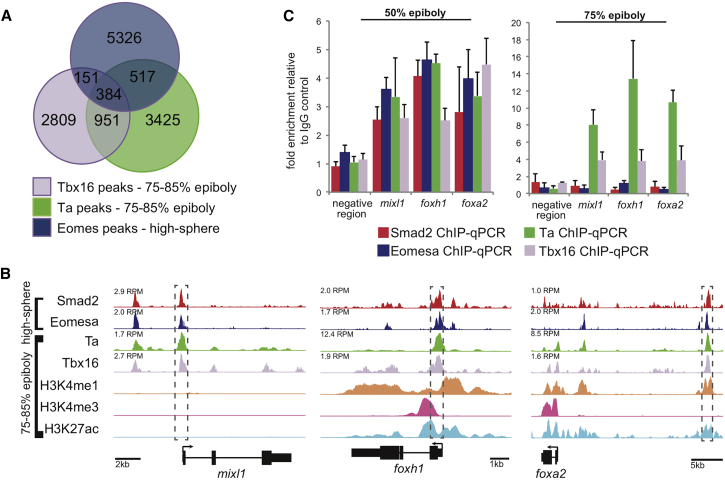
Comparison of Ta/Tbx16 Genomic Occupancy with Eomesa/Smad2 Reveals Direct Regulation of Endodermal Determinants prior to Gastrulation (A) Overlap of Ta and Tbx16 ChIP-seq peaks at 75%–85% epiboly (8–8.5 hpf) with Eomesa at high-sphere stage (3.3–4 hpf). (B) Smad2, Eomesa, Ta, Tbx16, H3K4me1, H3K4me3, and H3K27ac ChIP-seq peaks at indicated stages proximal to *mixl1*, *foxh1*, and *foxa2*. Peak heights in RPM are indicated. Boxed regions indicate peaks used for ChIP-qPCR validation. (C) ChIP-qPCR analysis of regions indicated in (B) at the indicated stages. Data are represented as mean ± SEM.

If co-expressed T-box TFs redundantly regulate a subset of targets via common CRMs, do Ta and Tbx16 share targets with Eomesa? We compared our Ta and Tbx16 ChIP-seq data with high-sphere stage (3.3–4 hpf) Eomesa and Smad2 ChIP-seq data (Nelson et al., 2014). Whereas these data are from a different developmental stage, they allowed us to test whether Ta/Tbx16/Eomesa ever occupy common CRMs. This revealed a subset of such sites, located near key genes regulating endoderm formation, such as *mixl1*, *foxh1*, and *foxa2* (Kikuchi et al., 2000, Nelson et al., 2014, Shin et al., 2008, Slagle et al., 2011; Figures 3A and 3B; Data S3). Ta and Tbx16 binding proximal to *foxh1* and *foxa2* correlates with functional chromatin marks at 80% epiboly (8.25 hpf; Figure 3B). *Mixl1* expression is restricted to earlier stages during endoderm specification (4–6 hpf; Kikuchi et al., 2000); hence, at 80% epiboly, *mixl1* lacks such chromatin marks (Figure 3B). However, we observe Tbx16 and Ta (as well as Eomesa and Smad2) binding by qPCR proximal to *mixl1* at 50% epiboly (5.3 hpf; Figure 3C), coincident with endoderm specification; thus, Ta and Tbx16 may positively regulate *mixl1* expression at this earlier stage.

**Figure 4:**
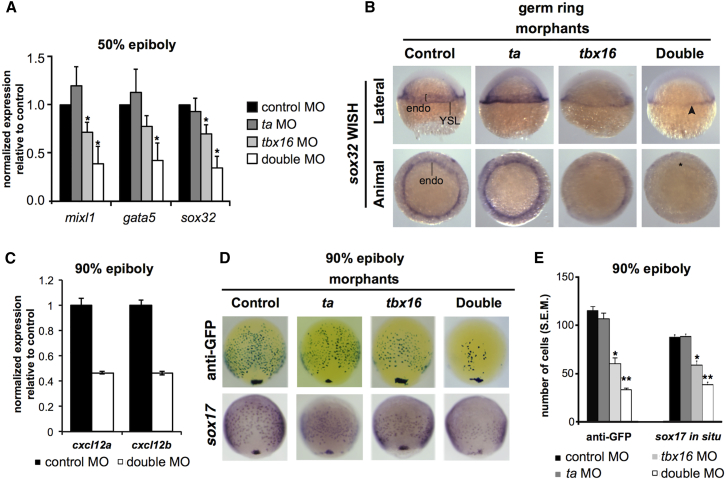
Loss of Ta and Tbx16 Leads to Downregulation of Endodermal Specifiers and Reduction of Endodermal Progenitors during Gastrulation (A) qPCR analysis of *mixl1*, *gata5*, and *sox32* at 50% epiboly (5.3 hpf) in single and double *ta/tbx16* morphants. All genes are significantly downregulated in double morphants; ^∗^p ≤ 5 × 10^−2^; Student’s t test. Data are represented as mean ± SEM. See also Figure S2. (B) WISH analysis of *sox32* in single and double morphants at germ ring stage (5.7 hpf). Arrowhead, YSL expression; ^∗^, loss of endoderm expression. (C) Microarray analysis at 90% epiboly (9 hpf) indicates downregulation of *cxcl12a/b*. Data are represented as mean ± SEM. (D) Immunological and WISH analysis of a *sox17*:*eGFP* transgene and endogenous *sox17* expression at 90% epiboly (9 hpf) in single and double morphants. (E) Cell numbers identified by immunostaining and WISH in (D). Cell numbers are representative of at least 20 embryos per condition. ^∗^p ≤ 1 × 10^−8^; ^∗∗^p ≤ 1 × 10^−20^; Student’s t test. Data are represented as mean ± SEM. See also Figure S3.

We also found Ta/Tbx16 binding proximal to *gata5* (Figure S2). To test whether Ta and Tbx16 are required for expression of *gata5*, *mixl1*, and their downstream target *sox32* (Bjornson et al., 2005), we examined *ta*, *tbx16*, and double morphants at 50% epiboly (5.3 hpf). Expression was weakly downregulated on *tbx16* KD and noticeably reduced on double KD (Figure 4A). Whole-mount in situ hybridization (WISH) revealed *sox32* expression was severely compromised in endoderm of double morphants, but not yolk syncytial layer (YSL), a region lacking *ta* and *tbx16* expression (Figure 4B). Thus, specification of endoderm progenitors requires both Ta and Tbx16, with Tbx16 having the greater effect.

Mesoderm-expressed chemokines *cxcl12a/b* were downregulated on *ta*/*tbx16* KD at 75% epiboly (8 hpf; Garnett et al., 2009) and our microarray dataset at 90% epiboly (9 hpf; Figure 4C; Data S4), demonstrating both *cxcl12a/b* are targets of Ta and Tbx16 (Figure 1G; Data S1). Thus, Ta and Tbx16 also regulate cell-extrinsic signaling cues driving proliferation of endodermal progenitors.

To evaluate endoderm expansion, we assayed expression of endoderm marker *sox17* at 90% epiboly (9 hpf). Immunodetection of a *sox17*:*eGFP* transgene and *sox17* WISH revealed endoderm cell numbers moderately reduced by *tbx16* KD and substantially reduced on *ta*/*tbx16* double KD (Figures 4D and 4E), strongly suggesting Ta and Tbx16 co-operatively promote both specification and expansion of the endoderm progenitor niche. In contrast, the *ta* paralog *tb* does not interact with *ta* in early endoderm formation because neither single nor *ta/tb* double KD significantly affected *sox17+* cell numbers at 90% epiboly (9 hpf; Figures S3A and S3B).

### Ta and Tbx16 Are Required for Correct Liver, Gut, and Pancreas Formation

**Figure 5:**
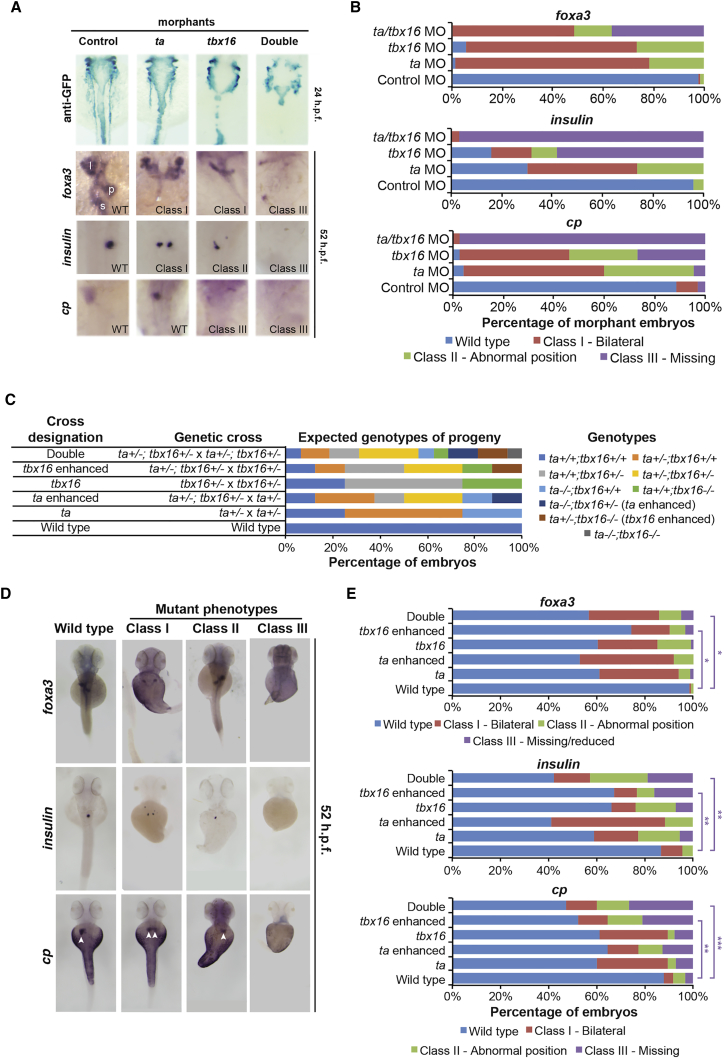
Ta and Tbx16 Are Redundantly Required for Liver, Pancreas, and Gut Development (A) GFP immunostaining in single and double morphant *sox17*:*eGFP* transgenic fish at 24 hpf and WISH analysis of broad endodermal organ marker *foxa3*, pancreas marker *ins*, and liver marker *cp* at 52–56 hpf in single and double morphants. l, liver; p, pancreas; s, stomach. Phenotypic classes as defined in (B) are indicated. (B) Percentage of KD embryos in each phenotypic class identified by WISH. Compare with Figure S3D. Graphs represent 19–124 embryos per group. (C) Genetic crosses and expected embryonic genotypes. *Ta*-enhanced (*ta*^*−/−*^;*tbx16*^*+/−*^) and *tbx16*-enhanced (*ta*^*+/−*^;*tbx16*^*−/−*^) genotypes are indicated. (D) Phenotypic classes of embryos from genetic crosses indicated in (C) identified by WISH. Arrowheads indicate liver *cp* staining. (E) Percentage of embryos in each phenotypic class from each genetic cross identified by WISH. Graphs represent 31–175 embryos per group. ^∗^p ≤ 3 × 10^−2^; ^∗∗^p ≤ 5 × 10^−3^; ^∗∗∗^p ≤ 1 × 10^−4^; all other comparisons with wild-type are not significant (p = 0.1–1); Fisher’s exact test. See also Figure S3.

If Ta and Tbx16 regulate endoderm specification and proliferation, we would expect gut and associated organs to form abnormally. Examination of *sox17*:*eGFP* at 24 hpf on double KD revealed the gut tube was severely compromised (Figure 5A), although pharyngeal endoderm remained intact. WISH analysis of broad endoderm (*foxa3*), pancreas (*ins*), and liver (*cp*) markers at 52–56 hpf revealed disordered liver and pancreas formation, such as laterality defects, in both *ta* and *tbx16* morphants (Amack et al., 2007, Danos and Yost, 1996), whereas Tbx16 morphants also display some loss of *ins* and *cp* expression. Double Ta/Tbx16 morphants, however, substantially lack *foxa3*, *ins*, and *cp* expression (Figures 5A and 5B). Double KD of Tb with Ta led to laterality defects (Figures S3C and S3D). Examination of *ta*^*+/−*^ and *tbx16*^*+/−*^ intercrosses revealed similar phenotypes to single Ta or Tbx16 morphants, whereas *ta*^*+/−*^;*tbx16*^*+/−*^ intercross embryos also gave phenotypes similar to Ta/Tbx16 double KD, at expected Mendelian ratios (Figures 5C–5E); thus, our observations are not artifacts of MO injection. Interestingly, intercrosses of *ta*^*+/−*^;*tbx16*^*+/−*^ with *tbx16*^*+/−*^ animals (*tbx16* enhanced) revealed that one wild-type *ta* allele is insufficient to rescue loss of *foxa3*, *cp*, and *ins* expression. In contrast, intercrosses of *ta*^*+/−*^;*tbx16*^*+/−*^ with *ta*^*+/−*^ animals (*ta* enhanced) that maintain a wild-type *tbx16* allele did not show significant loss of *ins* and *cp* expression, indicating Tbx16 has the greater influence on endoderm formation (Figures 5C–5E).

We conclude that Ta and Tbx16 play essential roles in endoderm progenitors, acting cell-autonomously via Mixl1 and non-autonomously governing cell-extrinsic Cxcl12a/b signaling pathways. Moreover, Ta and Tbx16 are redundantly required for correct gut, liver, and pancreas formation.

### Mixl1 Acting Downstream of Ta and Tbx16 Governs Nodal/Smad Target Gene Expression

**Figure 6:**
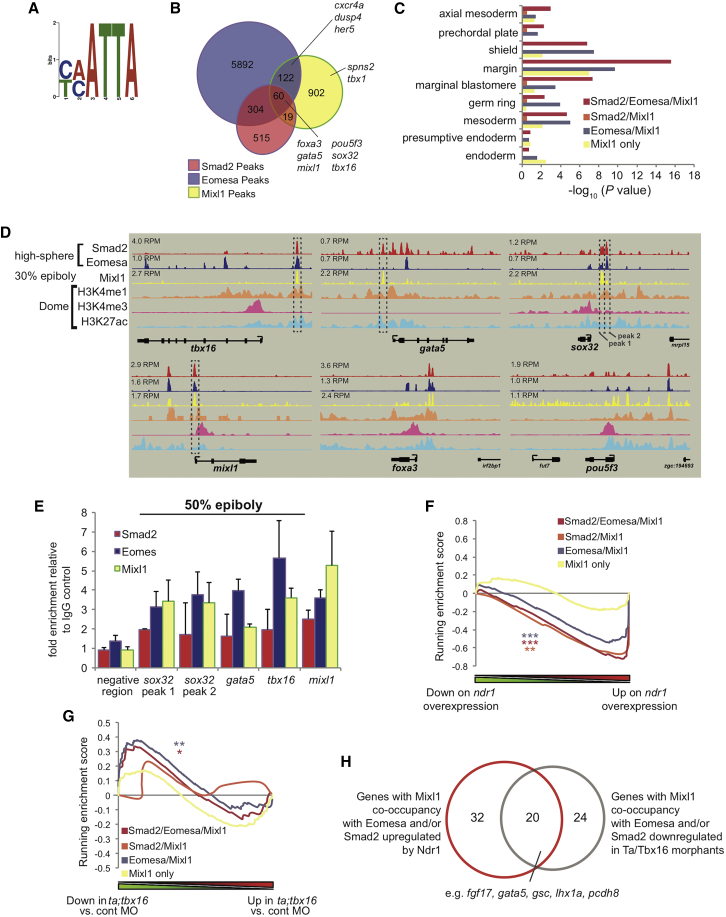
Mixl1 Occupies the Same Sites as Smad2 and Eomesa Proximal to Nodal-Responsive Endodermal Genes (A) Motif identified within Mixl1 ChIP-seq peaks using DREME; e = 2.7 × 10^−19^; p = 7.1 × 10^−24^. (B) Overlap of Eomesa and Smad2 ChIP-seq peaks at high-sphere stage (3.3–4 hpf) with Mixl1 peaks at 30%–50% epiboly (4.7–5.3 hpf). Endodermal regulators with occupancy of TFs are indicated. (C) Enrichment for genes with Eomesa and/or Smad2 and/or Mixl1 proximal binding (as indicated in B). The graph shows enrichment for cell types where *ndr1* and/or *ndr2* are expressed. (D) Smad2, Eomesa, Mixl1, H3K4me1, H3K4me3, and H3K27ac ChIP-seq at indicated stages proximal to *tbx16*, *gata5*, *sox32*, *mixl1*, *foxa3*, and *pou5f3*. Peak heights in reads per million (RPM) are indicated. Boxed regions indicate peaks used for ChIP-qPCR validation. (E) ChIP-qPCR analysis of regions indicated in (D) at 50% epiboly (5.3 hpf). Data are represented as mean ± SEM. (F and G) GSEA plots of genes with proximal binding of Mixl1 alone or at the same CRMs as Eomesa and/or Smad2 (defined and color-coded as in B) compared with microarray data: (F) changes in expression on *ndr1* overexpression in blastulae—Mixl1 binding with Eomesa and/or Smad2 is highly correlated with genes induced by Ndr1; (G) changes in expression on *ta/tbx16* KD at shield (6 hpf)—Mixl1 binding with Eomesa and Smad2 is highly correlated with downregulated genes. ^∗^FWER p ≤ 2 × 10^−2^; ^∗∗^FWER p ≤ 1 × 10^−3^; ^∗∗∗^FWER p ≤ 5 × 10^−4^. (H) Overlap of genes with occupancy of Mixl1 with Eomesa and/or Smad2 upregulated by Ndr1 (identified in F) or downregulated in Ta/Tbx16 morphants (identified in G). See also Figure S4.

To understand how transcriptional programs downstream of Ta and Tbx16 control endoderm formation, we next investigated Mixl1 target genes during endoderm specification by ChIP-seq at 30%–50% epiboly (4.7–5.3 hpf; Figure S4). De novo motif analysis identified a sequence closely resembling the previously described consensus binding motif within our ChIP-seq peaks (Zhang et al., 2009; Figure 6A).

We previously showed Smad2 and Eomesa bind CRMs proximal to Nodal-responsive genes (Nelson et al., 2014), and Mixl1 is known to physically interact with Smad2 at Nodal-responsive CRMs (Germain et al., 2000). Consistent with this, we observe Mixl1 occupancy at the same CRMs as Eomesa and Smad2 proximal to key regulators of endoderm formation (Figure 6B; Data S3). Targets with overlapping Eomesa, Smad2, and Mixl1 ChIP-seq peaks display expression domains co-localized with Nodal activity, further suggesting Mixl1 regulates similar Nodal targets to Eomesa/Smad2 (Figure 6C; Data S5). Moreover, common occupancy of Mixl1/Smad2/Eomesa at endoderm target genes is associated with functional chromatin marks at dome stage (4.3 hpf; Figure 6D). To confirm occupancy of Eomesa, Smad2, and Mixl1 at common target sites during endoderm specification, we performed ChIP-qPCR at 50% epiboly (5.3 hpf; Figure 6E). Comparison of our ChIP-seq data with microarray data on overexpression of the Nodal ligand *ndr1* in zebrafish blastulae (3.5–4 hpf; Nelson et al., 2014) revealed highly significant association between upregulated genes and binding of Mixl1 with Smad2 and/or Eomesa (Figure 6F). Genes with Mixl1 binding at the same CRMs as Eomesa and/or Smad2 are therefore induced by Ndr1, strongly suggesting Mixl1 co-operatively regulates Nodal target genes in association with Eomesa and Smad2. Intriguingly, 20 Ndr1-induced genes with proximal Mixl1/Smad2/Eomesa binding, including *gata5*, *gsc*, *wnt8a*, and *fgf8a* were also downregulated on Ta/Tbx16 double KD at shield stage (6 hpf; Figures 6G and 6H; Data S6). This suggests Ta and Tbx16 influence a subset of Nodal targets and that this may be partially due to their regulation of *mixl1*.

### Characterization of the Endodermal Gene Regulatory Network through Comparison of Mixl1, Smad2, Eomesa, Nanog, Mxtx2, and Pou5f3 Occupancy

**Figure 7:**
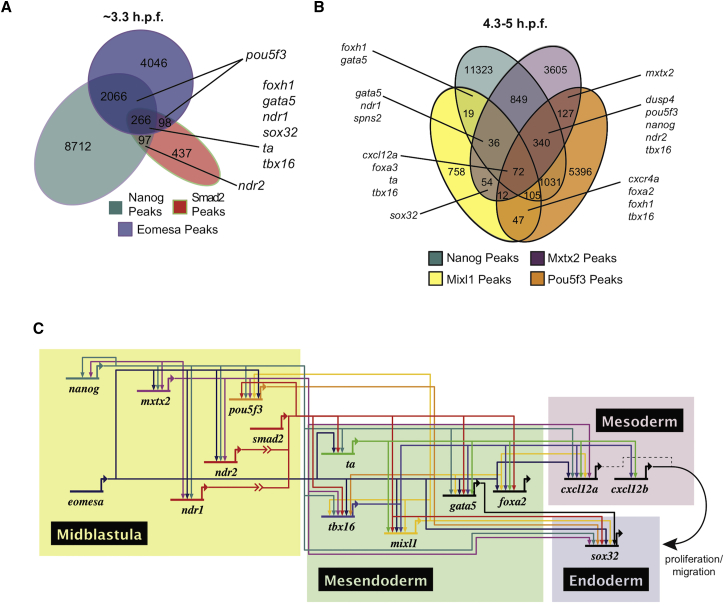
Nanog, Mxtx2, and Pou5f3 Occupy Sites Bound by Mixl1/Smad2/Eomesa Proximal to Key Endodermal Regulators (A) Overlap of Nanog, Smad2, and Eomesa ChIP-seq peaks at high stage (3.3 hpf). Endodermal regulators with occupancy of TFs are indicated. (B) Overlap of Nanog, Mxtx2, Pou5f3, and Mixl1 ChIP-seq peaks at 4.3–5 hpf. Endodermal regulators with occupancy of TFs are indicated. (C) A GRN for endoderm formation informed by this study. Links within the network represent binding identified by ChIP plus expression change in this or cited studies. Illustrated boxes contain the following: “midblastula”—factors implicated in mesendoderm induction, a subset of which are maternally contributed; “mesendoderm”—TFs induced at the margin between onset of zygotic transcription and gastrulation, promoting endoderm formation; “endoderm”—master regulator of zebrafish endoderm formation Sox32, which ensures endoderm fate specification; and “mesoderm”—secreted chemokines induced in at the margin and expressed by mesoderm to promote endoderm proliferation and migration. ≫ indicates ligand-receptor binding, leading to Smad2 activation. Dotted line indicates the reported minor influence of Cxcl12a compared with Cxcl12b (Boldajipour et al., 2011). See also Figures S5 and S6.

Because TFs Nanog, Mxtx2, and Pou5f3 also play known roles in endoderm formation in the blastula embryo (Lunde et al., 2004, Reim et al., 2004, Xu et al., 2012), we compared our Mixl1, Smad2, and Eomesa ChIP-seq data with that for Nanog, Mxtx2 (both 3.3 and 4.3 hpf), and Pou5f3 (5 hpf; Leichsenring et al., 2013, Xu et al., 2012). At high stage (3.3 hpf), although only a minority of Nanog peaks overlap with Smad2 and/or Eomesa, we found these common ChIP-seq peaks proximal to key endodermal regulators, such as *ndr1*, *gata5*, *sox32*, and *tbx16* (Figure 7A). Similarly, at dome-50% epiboly (4.3–5 hpf), despite limited Nanog, Mxtx2, Pou5f3, and Mixl1 peak overlap, these TFs display common binding at CRMs proximal to key endodermal regulators (Figure 7B; Data S7). Importantly, genes exhibiting proximal binding of multiple TFs were highly enriched for relevant developmental functions and expression patterns. Genes with proximal binding of individual TFs alone were notably less enriched for such terms (Figures S5 and S6), strongly suggesting that these four TFs perform their developmental roles in combination (Data S5 and S7).

Our analyses suggest the transcriptional processes underlying endoderm formation are complex, requiring coordinated temporal regulation of vital target genes by combinations of numerous TFs. An updated gene regulatory network (GRN) for endoderm formation incorporating this study is in Figure 7C.

## Discussion

The combinatorial requirement for Ta and Tbx16 in trunk and tail mesoderm formation has been known for 15 years (Amacher et al., 2002). Here, we report their redundant role in endoderm formation. Through characterizing genome-wide binding profiles of these TFs, we have identified a set of common target CRMs and regulated genes that can account for the action of Ta and Tbx16 in formation of mesoderm and endoderm. We find that Ta and Tbx16 bind and regulate *mixl1* expression, which is required for endoderm formation (mutated in *bonnie and clyde*; Kikuchi et al., 2000), suggesting that Ta and Tbx16 influence endoderm formation via Mixl1. To better understand how Mixl1 controls endoderm formation, we profiled its genomic binding and integrated these data with existing Eomesa, Smad2, Nanog, Mxtx2, and Pou5f3 early embryo datasets to present an augmented GRN for zebrafish endoderm formation.

### High-Affinity Binding of Ta and Tbx16 at Functional CRMs

Our data show that Ta and Tbx16 are able to bind a subset of the same CRMs. These common peaks are enriched for low numbers of consensus T-box motifs overlapping functional chromatin marks and are correlated with genes that mediate Ta and Tbx16 function. Conversely, CRMs bound by either Ta or Tbx16 only (distinct peaks) contain AC-rich sequences, as do common peaks that do not overlap functional chromatin. Previous work suggests that T-box TFs bind AC-rich sites with low affinity but the consensus site with high affinity (Evans et al., 2012). Our data therefore support the idea that the high-affinity sites are more efficiently bound and are important in targeting T-box TFs to functional CRMs.

Although Ta and Tbx16 bind common CRMs in vivo, whether they simultaneously bind the same CRM in the same cells or bind independently is unclear. Whereas our whole-embryo ChIP data cannot distinguish between these two possibilities, our previous in vitro electromobility shift analysis of a CRM controlling *dlc* expression suggests that, although four spatially distinct T-box sites are present, the CRM is only occupied by Ta or Tbx16 individually (Jahangiri et al., 2012). Whether this is also the case in vivo or at other CRMs remains to be determined. However, we note some tissue-specific binding may be detected in our data because Ta, which is expressed in notochord, binds CRMs in the vicinity of notochord genes, whereas Tbx16, which is not expressed in the notochord, does not bind these regions.

### Ta and Tbx16 in Formation of Mesendoderm

Through comparison of our Ta/Tbx16-binding data with that of maternal T-box TF Eomesa, which is involved in mesoderm and endoderm formation, we discovered all three TFs bind an overlapping set of CRMs, including those associated with endodermal genes, such as *mixl1*. This led us to ask whether Ta and Tbx16 are also involved in endoderm formation. Indeed, we show that embryos with reduced Ta and Tbx16 activity have reduced *mixl1* expression, less endoderm at the end of gastrulation, and consequently fail to form a complete gut and associated organs, the pancreas and liver.

In zebrafish, mesendoderm formation requires Nodal signaling (Schier, 2009), whereas FGF is required for correct mesoderm formation, in part via activation of *ta* and *tbx16*, and by antagonizing endoderm formation through phosphorylation and inactivation of Sox32 (Poulain et al., 2006). Nodal signaling, which is mediated by Smad2, is active in the first five or six cell tiers of the margin in the blastula embryo, whereas FGF activity extends further (van Boxtel et al., 2015), leading to the idea that cells closest to the margin become endoderm due to high levels of Nodal signaling, whereas endoderm fate is repressed by FGF further from the margin. During mesendoderm formation, Eomesa interacts with Smad2 and other TFs, including its downstream target Mixl1, to activate endodermal CRMs, although the requirement for Eomesa to activate endodermal genes is transient (Du et al., 2012). In response to Nodal, *ta* and *tbx16* are induced at the margin, coincident with endodermal genes, such as *gata5* (Rodaway et al., 1999) and *mixl1*.

Our data suggest that this expression of *ta* and *tbx16* at the margin prior to gastrulation is key to establishing *sox32* expression via upstream regulators, including *mixl1*, thus locking down endoderm fate, whereas presence of Ta and Tbx16 in cell tiers further from the margin lacking phospho-Smad2 would be insufficient to promote endoderm fate. It is also tempting to speculate that, with downregulation of Eomesa activity during blastula stages (Bruce et al., 2003), Ta and Tbx16 may target Smad2 to CRMs promoting endoderm fate. Consistent with this, we show co-occupancy of Ta/Tbx16/Smad2 at such elements. This idea is also supported by the observation that Mixl1 overexpression can only induce endoderm at the margin (Bjornson et al., 2005), where Ta and Tbx16 are expressed.

At the onset of gastrulation, *ta* and *tbx16* (along with their target *mixl1*) are rapidly downregulated in endoderm. At this stage, they appear to act in mesoderm to control *cxcl12a/b* expression, thus non-autonomously promoting correct endoderm migration and proliferation. Downstream endoderm lineages are therefore likely lost in mutants due to a diminished endoderm progenitor pool.

The function of Ta/Tbx16 in mesoderm may be in part independent of Nodal because Smad2 is absent from mesodermal progenitors further from the margin but may instead rely on interaction with BMP-regulated Smads, such as Smad1 (Faial et al., 2015, Messenger et al., 2005), as well as Smad-independent mechanisms.

### Conserved and Divergent Functions for T-Box Factors

T is highly conserved in sequence and function across all vertebrates, being required for notochord and posterior mesoderm formation (Naiche et al., 2005). Although not required, a role for T in endoderm formation may also be conserved. During mammalian stem cell differentiation, cells expressing T go on to form either mesoderm or endoderm, and T has been implicated in endoderm formation in mouse and human in vitro through binding endodermal CRMs (Faial et al., 2015, Lolas et al., 2014). In addition, in the presence of Smad2, with which it physically interacts, T is able to induce endoderm markers in differentiating human cells (Faial et al., 2015).

A role in endoderm formation for *tbx16* orthologs may also be conserved. For instance, the *Xenopus* ortholog of *tbx16*, *vegt* (Griffin et al., 1998), is required for endoderm formation (Zhang et al., 1998). Despite VegT being maternally contributed and *eomes* zygotically expressed (Showell et al., 2004)—the opposite of zebrafish where Tbx16 is zygotic and Eomesa maternal—the importance of Tbx16 to zebrafish endoderm formation highlights a clear parallel with *Xenopus*.

In mouse, of all T-box TFs, Eomes alone is required for endoderm formation (Arnold et al., 2008). It is interesting to note that *tbx16* orthologs are present in teleost, amphibian, and avian species but lost in mammals (Ahn et al., 2012); thus, it is possible that a key difference between zebrafish/*Xenopus* and mammals may be an increased dependency on Eomes due to loss of Tbx16/VegT.

### An Augmented GRN for Zebrafish Endoderm Formation

Mixl1 is key to endoderm formation, with mutants failing to form the vast majority of endoderm (Kikuchi et al., 2000), though its direct target genes in zebrafish were unknown aside from *sox32* (Bjornson et al., 2005). We identified a panel of candidate Mixl1 target genes sufficient to explain the loss of endoderm in *mixl1* mutants, such as *pou5f3* and *gata5*, acting upstream of *sox32* (Kikuchi et al., 2001, Lunde et al., 2004), as well as *sox32* itself.

Previous understanding of the GRN controlling early zebrafish endoderm formation involved maternally contributed TFs, including Eomesa and Nanog, combining to induce YSL formation via Mxtx2, leading to Nodal production. Along with other maternal TFs, such as Pou5f3 and Smad2, they also control expression of key endoderm determinants, such as *gata5*, *mixl1*, and *sox32* in the emerging mesendoderm (Bjornson et al., 2005, Bruce et al., 2005, Dubrulle et al., 2015, Lunde et al., 2004, Reim et al., 2004, Xu et al., 2012). Combinations of these TFs positively regulate *sox32* expression, thus establishing endodermal fate. This study reveals roles for Ta and Tbx16 within this GRN, through regulation of *mixl1* and *cxcl12a/b*.

Intriguingly, our analyses also indicate that a subset of CRMs are bound by combinations of Eomesa, Smad2, Mixl1, Nanog, Mxtx2, and Pou5f3, including those proximal to other genes implicated in endoderm formation, such as *dusp4* (Brown et al., 2008), *cxcr4a* (Stückemann et al., 2012), and *spns2* (Osborne et al., 2008). It will be interesting to learn more about how these TFs collectively contribute to the function of the identified CRMs.

Whereas this study focused on endoderm formation, Ta, Tbx16, and Mixl1 also have key functions in mesoderm formation, which are represented in our data. We therefore provide a rich resource for future study, as well as adding additional players to the story of endoderm formation.

## Experimental Procedures

Details of immunohistochemistry, in situ hybridization, qRT-PCR, ChIP-qPCR, and cell counting are provided in Supplemental Experimental Procedures.

### Animals

AB, *ta*^*b*195/+^, *tbx16*^*b*104/+^, and *ta*^*b*195/+^;*tbx16*^*b*104/+^ fish were reared as described (Westerfield, 2000). All zebrafish studies complied fully with the UK Animals (Scientific Procedures) Act 1986 as implemented by King’s College London.

### Morpholino Injection

One-cell stage embryos were injected with 0.5 pmol *tbx16* (Bisgrove et al., 2005) and 0.25 pmol *ta* (Feldman and Stemple, 2001), which recapitulate the mutant *ta* and *tbx16* phenotypes, respectively, or equivalent quantities of standard control MO (GeneTools).

### ChIP-Seq and Data Analysis

For ChIP-seq, two independent replicate experiments were performed using 5,000 embryos each at the indicated developmental stage as described (Nelson et al., 2014) using previously characterized anti-Ta (Morley et al., 2009, Schulte-Merker et al., 1992) and anti-Tbx16 antibodies (Amacher et al., 2002, Garnett et al., 2009, Jahangiri et al., 2012) or a commercial anti-Mixl1 antibody (Anaspec 55613; Figure S4). Reads were mapped to the Zv9 zebrafish genome with Bowtie (Langmead et al., 2009) in Galaxy (Giardine et al., 2005, Goecks et al., 2010) using default parameters with the exceptions: -y -m2 -k2 –best (Table S1). We therefore used a maximum of two acceptable alignments, ensuring that best possible alignments were identified. Peak calling, relative to matched input samples, was performed using MACS (Zhang et al., 2008) with the parameters: Ta – m-fold 10, p value 1e−8; Tbx16 – m-fold 10, p value 1e−4; and Mixl1 – m-fold 10, p value 1e−5. For Ta ChIP-seq, one replicate gave lower signal-to-noise ratio. We therefore used the stronger replicate for further analyses. Key peak were validated by ChIP-qPCR (Figures 1H, 2D, and 3C). Peaks identified in both replicates are indicated in Data S1.

Histone ChIP-seq data were downloaded from NCBI GEO: GSE32483, mapped to the Zv9 genome as above, and peaks called using default MACS parameters with one exception (m-fold 20).

Other ChIP-seq data were downloaded from GEO: Nanog and Mxtx2, GEO: GSE34683; Pou5f3, GEO: GSE39780; and Eomesa and Smad2, GEO: GSE51894.

Peaks were associated with genes by annotating the nearest transcription start site ± 100 kb. Functional annotation analysis was performed using DAVID (Huang et al., 2009a, Huang et al., 2009b). De novo motif discovery was performed using Weeder (Pavesi et al., 2006) for Ta and Tbx16 peaks and DREME (Bailey, 2011) for Mixl1.

### Microarray Experiments

Data for morphant embryos were generated at shield (6 hpf) and 90% epiboly (9 hpf) using Agilent Zebrafish Gene Expression Microarrays (V3) and analyzed as previously described (Nelson et al., 2014). Microarray data for control, *ta*, *tbx16*, and *ta*;*tbx16* double morphants at 75% epiboly (8 hpf; Garnett et al., 2009) were downloaded from GEO: GSE12857, cyclic loess normalized, and differential expression determined using the R package oneChannelGUI (Sanges et al., 2007). Data for *ndr1* overexpression in blastulae were previously described (Nelson et al., 2014; GEO: GSE51894).

For GSEA (Mootha et al., 2003, Subramanian et al., 2005), GSEA v2.2.2 was used applying 2,000 permutations to gene lists preranked on statistics obtained from microarray data analysis. To be sufficiently stringent when multiple gene sets were analyzed, the family-wise error rate (FWER) p value was used to establish significance.

### Statistical Analysis

Quantitative data, including expression levels and cell counts, are expressed as mean ± SEM. Differences between groups were compared with a two-tailed Student’s t distribution test. Differences in qualitatively scored phenotypes from mutant and wild-type matings or in knockdown experiments were compared using chi-square test. Differences in overlap of Ta and Tbx16 ChIP-seq peaks with histone marks were also compared using chi-square test. All tests were performed with a confidence level of 95%.

## Author Contributions

A.C.N. and F.C.W. conceived the study, performed the experiments, and wrote the manuscript; S.J.C., S.N.G., and I.D. performed the experiments; and D.L.S. participated in and facilitated acquisition of ChIP-seq data. All authors read and approved the final manuscript.

## Accession Numbers

The accession number for the microarray and ChIP-seq data reported in this paper is GEO: GSE84619.

## Supplemental Information

Document S1. Supplemental Experimental Procedures, Figures S1–S6, and Table S1

Data S1. Ta and Tbx16 ChIP-Seq Coordinates, Related to Figure 1 The nearest TSS ±100kb of each peak and associated gene descriptions and distance from the peak are provided, as well as whether each peak overlaps H3K4me1, H3K4me3 and H3K27ac ChIP-seq peaks from the Bogdanovic et al. (2012) dataset (relates to Figure 1).

Data S2. Anatomical and Functional Annotation Analysis of Genes Marked by Common and Distinct Ta and Tbx16 Peaks, Related to Figure 2

Data S3. Overlap of Ta and Tbx16 ChIP-Seq Peaks with Eomesa ChIP-Seq Peaks plus Mixl1 ChIP-Seq Peak Overlaps with Eomesa and Smad2 ChIP-Seq Peaks, Related to Figures 3 and 6 The nearest TSS ±100kb of each peak and associated gene descriptions are provided.

Data S4. Microarray Data Analysis of Wild-Type versus *ta*;*tbx16* Double Morphants at 90% Epiboly, Related to Figure 4

Data S5. Anatomical Annotation Analysis of Genes Marked by Mixl1 ChIP-Seq Peaks Overlapping Smad2 and/or Eomesa ChIP-Seq Peaks, Related to Figure 6 Also anatomical and functional annotation analysis of genes marked by Mixl1, Nanog, Mxtx2 and/or Pou5f3 related to Figures S5 and S6.

Data S6. Microarray Data Analysis of Wild-Type versus *ta*;*tbx16* Double Morphants at Shield Stage, and List of 20 Genes from Leading Edge Analysis Highlighted in Figure 6H

Data S7. Tables of Genomic Coordinates Occupied by Two or More of Eomesa, Mixl1, Mxtx2, Nanog, Pou5f3, and Smad2, Related to Figure 7 The nearest TSS ±100kb of each peak and associated gene descriptions are provided.

Document S2. Article plus Supplemental Information
