# Supplementary material for: In Vivo Regulation of the Zebrafish Endoderm Progenitor Niche by T-Box Transcription Factors
Source: Cell Rep. 2017 Jun 27;19(13):2782–95. doi: 10.1016/j.celrep.2017.06.011 (PMC5494305; doi:10.1016/j.celrep.2017.06.011)
Supplement: Document S1. Supplemental Experimental Procedures, Figures S1–S6, and Table S1 [file mmc1.pdf]

Cell Reports, Volume *19*

Supplemental Information

In Vivo Regulation of the Zebrafish Endoderm

Progenitor Niche by T-Box Transcription Factors

Andrew C. Nelson, Stephen J. Cutty, Saule N. Gasiunas, Isabella Deplae, Derek L. Stemple, and Fiona C. Wardle

Supplemental Figures

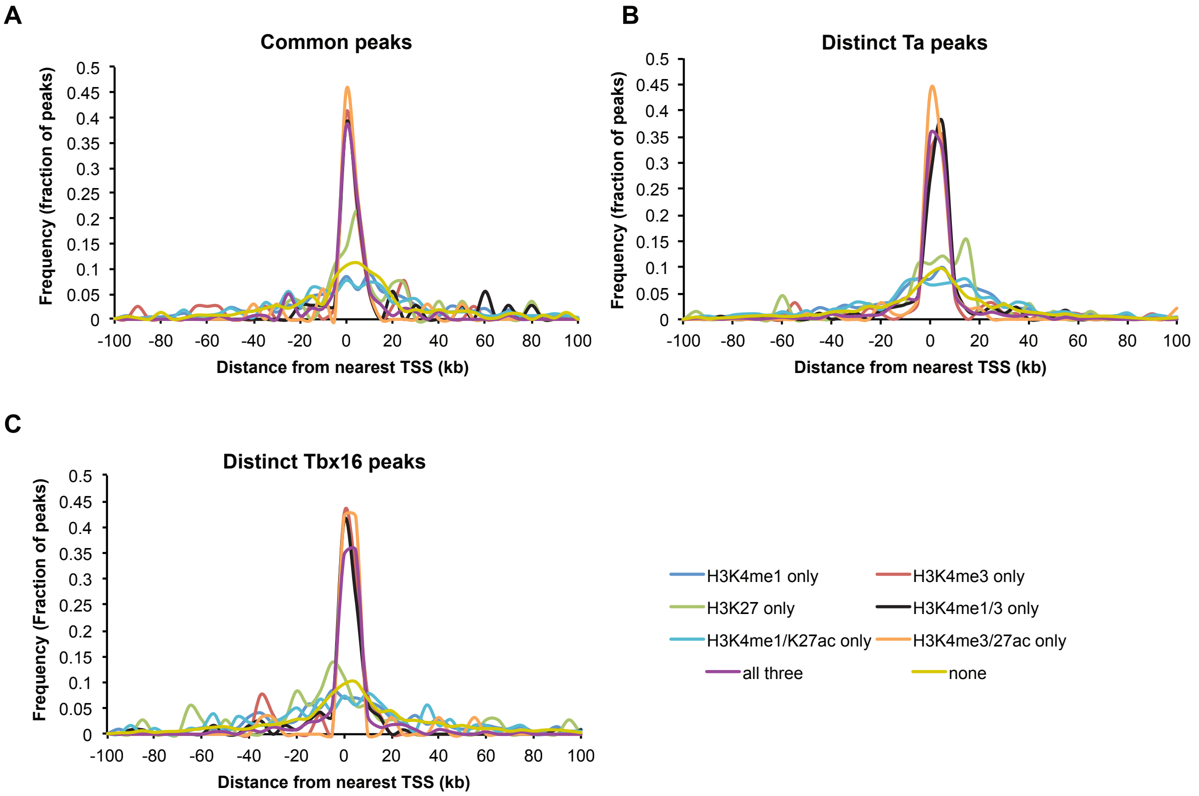

Figure S1 (relates to Figure 1). Distribution of Ta and Tbx16 peaks overlapping histone marks relative to the nearest TSS ±100kb.

(A-C) Graphs indicating the distribution of Ta/Tbx16 common and distinct peaks at each combination of histone marks as defined in Figure 1H. Though nearest TSS ±100kb were annotated the majority of peaks are markedly closer. As expected, Ta and Tbx16 ChIP-seq peaks overlapping H3K4me3 (putative promoters) tend to occur in close proximity to TSSs, while those overlapping H3K4me1 (putative enhancers) are more distant.

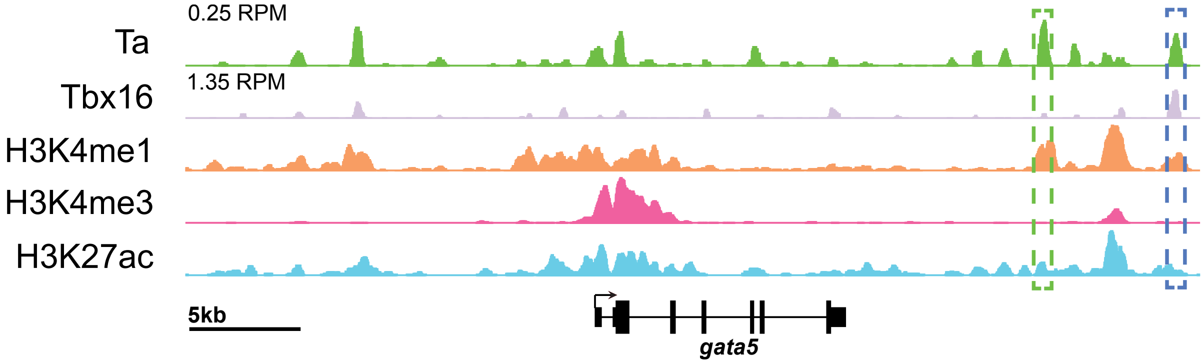

Figure S2 (relates to Figure 4). Endodermal regulator *gata5* exhibits proximal binding of Ta and Tbx16. Stage-matched Ta, Tbx16, H3K4me1, H3K4me3 and H3K27ac and ChIP-seq at the *gata5* locus. Peak heights in reads per million (RPM) are indicated. Common (blue box) and Distinct Ta (green box) peaks called as significant are indicated.

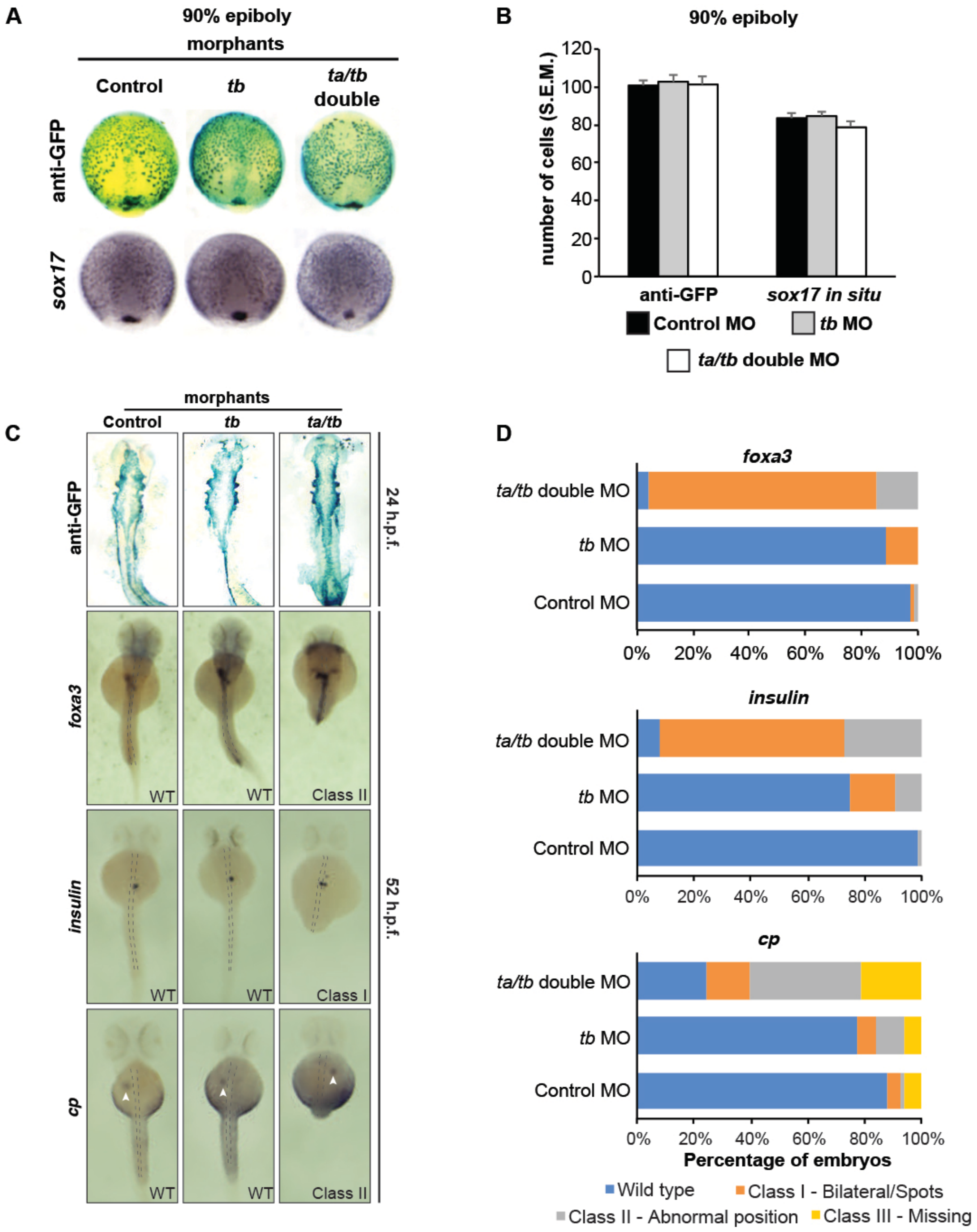

Figure S3 (relates to Figures 4 and 5). Morpholino knockdown of Brachyury homologue Tb does not substantially enhance the Ta-mediated effects on endoderm progenitor numbers at 90% epiboly, but does enhance downstream laterality defects. (A) Immunological and WISH analysis of a *sox17:eGFP* transgene and endogenous *sox17* expression at 90% epiboly (9 h.p.f.) in single and double morphants as indicated. (B) Quantification of cell numbers identified by immunostaining and WISH in panel A. Cell numbers are representative of at least 20 embryos per condition. (C) Anti-GFP immunostaining of flat-mounted *sox17:eGFP* embryos at 24 h.p.f. and WISH analysis of broad marker of endodermal organs *foxa3*, pancreas marker *ins* and liver marker *cp* at 52-56 h.p.f. in single and double morphants. Examples of phenotypic classifications are indicated. WT = wild type. Dashed lines indicate the midline. (D) Phenotypic quantification of morphant embryos from panel C experiment. Each group is representative of at least 30 embryos.

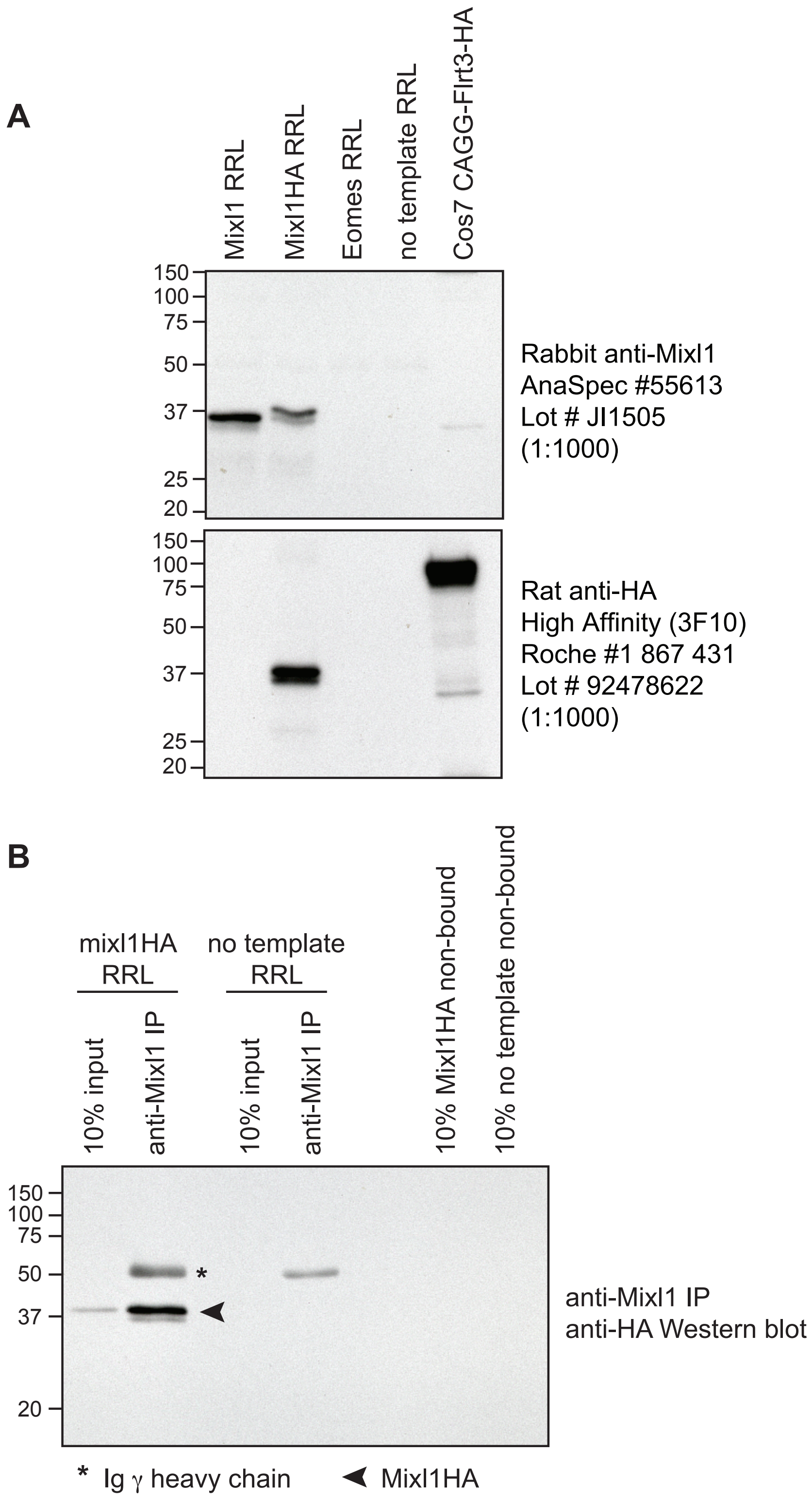

Figure S4 (relates to Figure 6). The rabbit polyclonal antibody used for anti-Mixl1 ChIP-seq recognizes Mixl1 protein in both Western blot and immunoprecipitation assays. (A) Western blot demonstrating that the anti-Mixl1 rabbit polyclonal antibody cleanly recognizes HA-tagged and untagged Mixl1 protein produced in rabbit reticulocyte lysate (RRL), whereas a rat anti-HA monoclonal antibody only recognizes HA-tagged proteins. Molecular weight standards in kDa are indicated. (B) Immunoprecipitation of Mixl1HA protein using anti-Mixl1 rabbit polyclonal antibody followed by anti-HA Western blot demonstrates that Mixl1 protein was efficiently captured.

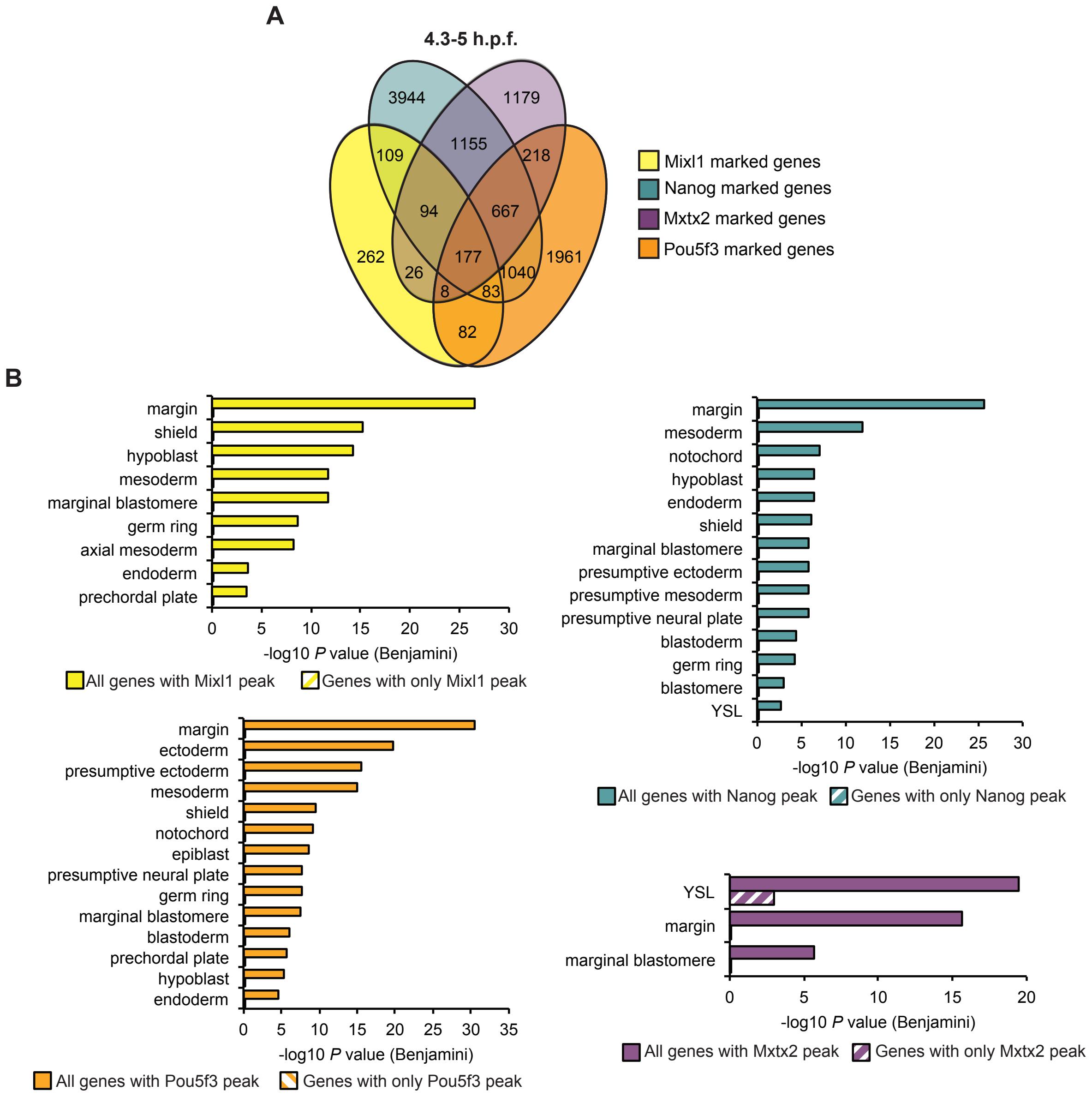

Figure S5 (relates to Figure 7). Common putative targets of Mixl1/Nanog/Mxtx2/Pou5f3 correlate with their expression patterns in the early embryo.

(A) Venn diagram indicating the number of genes with binding of Mixl1, Nanog, Mxtx2 or Pou5f3 (nearest TSS ±100kb; different to Figure 7B which indicated the overlap between ChIP-seq peaks – this Venn diagram indicates number of genes marked by ChIP-seq peaks regardless of whether the peaks overlap). Note the lower proportion of genes marked by a single factor, compared with ChIP-seq peaks unique to a single factor. This is due to many genes displaying binding for multiple factors at different CRMs.

(B) Anatomical enrichment analysis of total genes bound by each factor (nearest TSS ±100kb), or of genes bound by only each individual factor. Analysis was performed relative to anatomical expression patterns recorded in the ZFIN database using DAVID. The reported terms are for cell types/tissues where the factors are reported to be expressed, or which are known to be directly altered in loss-of-function mutants. Note that with the exception of Mxtx2, genes showing binding for individual factors alone are poorly enriched for anatomical terms. Mxtx2 alone shows binding at YSL genes as expected for this YSL expressed factor. This analysis suggests combinations of these four factors regulate their known cell type-specific functions. Tables of significant functional and anatomical terms are in Data S5.

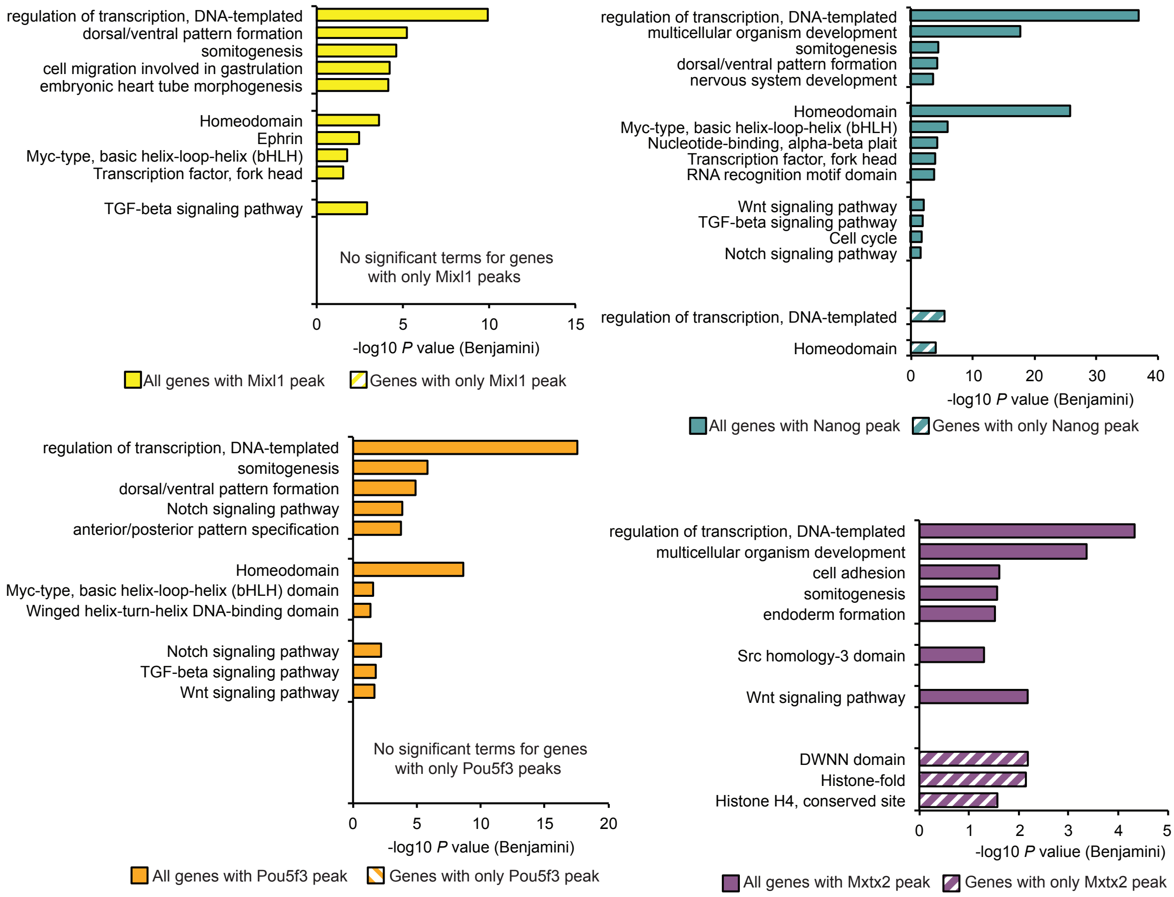

Figure S6 (relates to Figure 7). Genes with binding of individual factors show poor enrichment for functional annotation terms.

Reported for each of Mixl1, Nanog, Pou5f3 and Mxtx2 are the five most significant (fewer if fewer significant terms) non-redundant Gene Ontology Biological Process, InterPro and KEGG Pathway terms significantly enriched amongst genes with ChIP-seq peaks (nearest TSS ±100kb). Note that with the exception of Mxtx2, genes showing binding for individual factors alone are poorly enriched for functional terms. This analysis strongly suggests that these factors act in combination to regulate their target genes in early development. Tables of significant functional and anatomical terms are in Data S5.

Factor	Stage	Sample	Read length	Total number of reads	Final number of mapped reads	Percentage of total
**Ta**	75-85%	ChIP1	38	11,396,372	2,779,764	24.4
	epiboly	ChIP2	38	25,406,256	6,021,884	23.7
		Input	38	10,690,296	2,692,914	25.2
**Tbx16**	75-85%	ChIP1	38	31,622,398	6,957,583	22.0
	epiboly	ChIP2	38	29,880,883	7,173,663	24.0
		Input	38	12,412,184	4,850,591	39.1
**Mixl1**	30-50%	ChIP1	63	29,671,488	5,913,904	19.9
	epiboly	ChIP2	63	239,043,044	7,984,896	3.3
		Input	63	107,848,351	10,018,032	9.3

Table S1 (relates to Figures 1-3 and 6-7). Summary of reads and mapping statistics for Ta, Tbx16 and Mixl1 ChIP-seq experiments.

Supplemental Data

Data S1. Ta and Tbx16 ChIP-seq coordinates. The nearest TSS ±100kb of each peak and associated gene descriptions and distance from the peak are provided, as well as whether each peak overlaps H3K4me1, H3K4me3 and H3K27ac ChIP-seq peaks from the Bogdanovic *et al.* (2012) dataset (relates to Figure 1).

Data S2. Anatomical and functional annotation analysis of genes marked by common and distinct Ta and Tbx16 peaks (relates to Figure 2).

Data S3. Overlap of Ta and Tbx16 ChIP-seq peaks with Eomesa ChIP-seq peaks, plus Mixl1 ChIP-seq peak overlaps with Eomesa and Smad2 ChIP-seq peaks (relates to Figures 3 and 6). The nearest TSS ±100kb of each peak and associated gene descriptions are provided.

Data S4. Microarray data analysis of wild type vs. *ta;tbx16* double morphants at 90% epiboly (9 h.p.f.; relates to Figure 4).

Data S5. Anatomical annotation analysis of genes marked by Mixl1 ChIP-seq peaks overlapping Smad2 and/or Eomesa ChIP-seq peaks (relates to Figure 6). Also anatomical and functional annotation analysis of genes marked by Mixl1, Nanog, Mxtx2 and/or Pou5f3 related to Figure S5-6.

Data S6. Microarray data analysis of wild type vs. *ta;tbx16* double morphants at shield stage (6 h.p.f.), and list of 20 genes from leading edge analysis highlighted in Figure 6H.

Data S7. Tables of genomic coordinates occupied by two or more of Eomesa, Mixl1, Mxtx2, Nanog, Pou5f3 and Smad2 (relates to Figure 7). The nearest TSS ±100kb of each peak and associated gene descriptions are provided.

Supplemental Experimental Procedures

RNA extraction, cDNA synthesis and qRT-PCR

Extraction of RNA, synthesis of cDNA and qPCR was performed as reported previously (Cutty et al., 2012). The primers used for qRT-PCR analysis are listed below:

gene	forward	reverse	reference
sox32	CCGTACATGCAAGAAGCAGA	ATCAAAGGTGGCATTTGGAG	
mixl1	ACCCAAAGGAACACGAACAG	AACAGGCGATGTGTTTAGGG	
gata5	AGAGACGGAACCGGACACTA	TCTCCTCCACAGTGTTGTCG	

ChIP-qPCR analysis

ChIP-qPCR was performed on approximately 300 to 500 embryos at the indicated stages using 1ul anti-Mixl1 antibody (Anaspec 55613, lot number JI1505), 3ul anti-Ta antibody, 3ul anti-Tbx16 antibody, 3ul anti-Eomesa antibody, 0.5ug anti-Smad2 antibody (clone 31H15L4; Thermo Fisher Scientific) and normal rabbit IgG as described (Garnett et al., 2009; Jahangiri et al., 2012; Morley et al., 2009; Nelson et al., 2014). qPCR was carried out on a Mx3005P (Agilent Technologies, Santa Clara, CA, USA) using SYBR Green 1 Master kit (Roche Basel, Switzerland) according to the manufacturer's instructions. Values for each condition were calculated as a percentage of input and are presented as fold enrichment relative to the IgG control. Primer sequences were as follows:

gene	forward	reverse	reference
col8a1a	ATGGTGTCTTTGCATGCGTG	GCCTCTAATGGACGTCTGCT	
p4ha2	ACCCGACTGCTTCCTGTTAA	GCTGTTCTGAAAAGCCGACT	
etv4	GTGGGAGTTGACTGAATGGC	AGGTGGGAGCTGGACTTTAC	
eve1	CCTCCAGAAAAGCTTTCTTCT CTAT	CAGAGGGAGGTGTTAAATTGTC TT	Morley et al., 2009
itga6a	TCACATTCTTCCGGCTTCCT	TGGCAGCTCGTATCTCTCTG	
dmd	TCGCATTGTCGAGAGTTCCA	CCCTCGCTGTCACACATAGT	
tbx16 peak	CAGAAAGCAAACCCCTCGAG	GCATGAATAGAGAGCCTGCTG	
cxcl12b	TCTGACACCTCACACATGCA	GCAGATTTGGGAGTTCAGCC	Nelson et al., 2014 (Eomes peak 3484)
mixl1 peak	ATTAAACTCGCACACGAACCT T	CCTGAAATGAGTGGCTTTTCTT	Nelson et al., 2014 (Common Peak 187)
foxh1	TCTGCAGTCAAGGTGATGGT	CAGTCTGAGGGGTTGAGGAG	
foxa2	CCTGTGGCCCAATTGAAGAG	CCTGCGAGTGACTGCAATAC	
sox32 peak 1	GCCCGTGTGTAGTGAGAGAT	AACTGCGCGACTATTTCTGA	
sox32 peak 2	AATTGTGCGAAATGGCCAC	ACTACAGCACAGTCACACGT	
gata5 peak	TAAGCCGTGTAACCATGCAG	TGATGTTGCGACCTGTGAAC	
Negative region (*rhod*)	GACTCCACACAATCTGCAACA T	ACCACCTACGCTAAAGAAACCA	Morley et al., 2009

Whole-mount immunostaining and *in situ* hybridization

Embryos fixed in 4% PFA were washed (wash buffer: Phosphate Buffered Saline (PBS);1%DMSO; 0.1%Triton X-100), blocked (2% goat serum;1% Bovine Serum Albumin (BSA) in wash buffer) for 1 hour at room temperature, rewashed and incubated with mouse monoclonal anti-GFP 3E6 IgG2a in wash buffer (A11120, Molecular Probes, 1:300 in block) overnight at 4^oC. Embryos were rewashed, reblocked and incubated with Goat anti-mouse HRP- conjugate in wash buffer (Thermo Scientific diluted to 1µg/ml in block) for four hours at room temperature. Embryos were rewashed, developed using HistoGreen (Linaris) according to the manufacturer's instructions, rewashed, processed through a glycerol gradient and imaged on a Leica MZ16F dissecting

microscope. 24 h.p.f. were permeabilized with ice cold acetone for 7 minutes at -20^oC prior to primary antibody incubation.

In situ hybridization was performed as described (Jowett and Lettice, 1994). Dioxygenin-labelled RNA probes for *sox32* (Alexander et al., 1999), *sox17* (Alexander and Stainier, 1999), *foxa3* (Odenthal and Nusslein-Volhard, 1998), *ins* (Milewski et al., 1998) and *cp* (Korzh et al., 2001) were produced as previously described.

Cell counting
Photographs of each embryo were taken from a dorsal perspective and using PowerPoint, sharpness was increased to 100% on each photograph to enhance contrast between stained cells and background. A grid was overlaid to ensure the orientation of the embryo was the same in each photograph, with the embryonic midline central on the grid and the dorsal forerunner cells located in the bottom central square, and the cells were manually counted.

Tb morpholino injection
0.5 pmol of *tb* translation blocking morpholino (GACAGTCACCGGAGTCCATTCTATC; Martin and Kimelman, 2008) was injected with 0.25 pmol of the p53 translation blocking morpholino (GCGCCATTGCTTTGCAAGAATTG; Langheinrich et al., 2002) into one-cell stage embryos.

***In vitro* protein production and Western blot analyses**
Mixl1 expression constructs were pCS2+ *mixl1*, pCS2+ *mix1HA* and pCS2+ *Eomesodermin*. Plasmids were linearized using NotI. Linearized constructs were transcribed with SP6 polymerase to generate capped mRNA according to standard protocols. Capped mRNA for each protein was used to synthesize protein in rabbit reticulocyte lysate according to manufacturer's instructions (Promega). Reticulocyte lysates were subjected to SDS/PAGE on a 10% gel, then blotted to PVDF membrane (GE Healthcare) according to standard protocols. Membranes were blocked for 1 hour at room temperature in 5% skimmed milk powder in Tris buffered saline/0.1% Tween 20 (TBST), then incubated for 16 hours at 4^oC in antibodies diluted in block as indicated in Figure S2. Blots were then washed extensively in TBST, incubated for 1 hour in either anti-rat or anti-rabbit HRP-conjugated antibodies (GE Healthcare), washed extensively in TBST before detection with Amersham ECL Prime Western Blotting Detection Reagent (GE Healthcare).

Immunoprecipitation
1μl of *in vitro* translated Mixl1HA protein was incubated for 16 hours with 1μg of anti-Mixl1 rabbit polyclonal antibody (Anaspec 55613, lot number JI1505) and 15μl BSA-blocked Dynabeads Protein G (ThermoFisher) at 4 ^oC followed by extensive washing with RIPA buffer. Dynabeads were then boiled in 1x SDS sample buffer and eluate subjected to anti-HA Western blot analysis.

Supplemental References

Alexander, J., Rothenberg, M., Henry, G.L., and Stainier, D.Y. (1999). casanova plays an early and essential role in endoderm formation in zebrafish. Dev Biol *215*, 343-357.
Alexander, J., and Stainier, D.Y. (1999). A molecular pathway leading to endoderm formation in zebrafish. Curr Biol *9*, 1147-1157.
Cutty, S.J., Fior, R., Henriques, P.M., Saude, L., and Wardle, F.C. (2012). Identification and expression analysis of two novel members of the Mesp family in zebrafish. Int J Dev Biol *56*, 285-294.
Korzh, S., Emelyanov, A., and Korzh, V. (2001). Developmental analysis of ceruloplasmin gene and liver formation in zebrafish. Mech Dev *103*, 137-139.
Langheinrich, U., Hennen, E., Stott, G., and Vacun, G. (2002). Zebrafish as a model organism for the identification and characterization of drugs and genes affecting p53 signaling. Curr Biol *12*, 2023-2028.
Milewski, W.M., Duguay, S.J., Chan, S.J., and Steiner, D.F. (1998). Conservation of PDX-1 structure, function, and expression in zebrafish. Endocrinology *139*, 1440-1449.
Odenthal, J., and Nusslein-Volhard, C. (1998). fork head domain genes in zebrafish. Dev Genes Evol *208*, 245-258.
