# Supplementary material for: In Vivo Regulation of the Zebrafish Endoderm Progenitor Niche by T-Box Transcription Factors
Source: Cell Rep. 2017 Jun 27;19(13):2782–95. doi: 10.1016/j.celrep.2017.06.011 (PMC5494305; doi:10.1016/j.celrep.2017.06.011)
Supplement: Document S2. Article plus Supplemental Information [file mmc9.pdf]

Cell Reports

Article

In Vivo Regulation of the Zebrafish Endoderm Progenitor Niche by T-Box Transcription Factors

Graphical Abstract

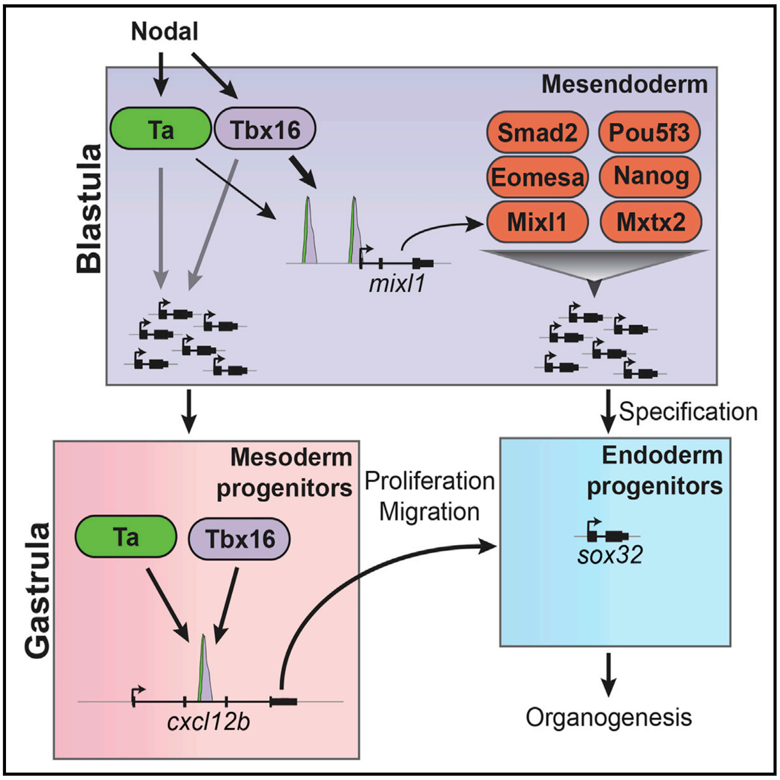

Highlights

- Ta and Tbx16 redundantly regulate genes through common *cis*-regulatory modules
- Ta and Tbx16 control expression of *mixl1* and other key endodermal regulators
- Mixl1 occupies endodermal CRMs with Smad2, Eomesa, Nanog, Mxtx2, and Pou5f3
- *Ta* and *tbx16* double mutants fail to correctly form liver, pancreas, and gut tube

Authors

Andrew C. Nelson, Stephen J. Cutty, Saule N. Gasiunas, Isabella Deplae, Derek L. Stemple, Fiona C. Wardle

Correspondence

a.nelson.1@warwick.ac.uk (A.C.N.), fiona.wardle@kcl.ac.uk (F.C.W.)

In Brief

Endoderm contributes to the respiratory and gastrointestinal tracts and all associated organs. How transcription factors control endodermal progenitor specification and expansion is not completely understood. Here, Nelson et al. identify a key redundant requirement for T-box factors Ta and Tbx16 in zebrafish endoderm formation and explore the downstream transcriptional programs.

Accession Numbers

GSE84619

Nelson et al., 2017, Cell Reports *19*, 2782–2795
June 27, 2017 © 2017 The Author(s).
http://dx.doi.org/10.1016/j.celrep.2017.06.011

OPEN ACCESS
CellPress

Cell Reports
Article

In Vivo Regulation of the Zebrafish Endoderm Progenitor Niche by T-Box Transcription Factors

Andrew C. Nelson,[1,2,4,5,*] Stephen J. Cutty,[1] Saule N. Gasiunas,[1] Isabella Deplae,[1] Derek L. Stemple,[3] and Fiona C. Wardle[1,*]
[1]Randall Division of Cell and Molecular Biophysics, King's College London, London SE1 1UL, UK
[2]Sir William Dunn School of Pathology, University of Oxford, Oxford OX1 3RE, UK
[3]Wellcome Trust Sanger Institute, Hinxton, Cambridge CB10 1SA, UK
[4]School of Life Sciences, University of Warwick, Coventry CV4 7AL, UK
[5]Lead Contact
*Correspondence: a.nelson.1@warwick.ac.uk (A.C.N.), fiona.wardle@kcl.ac.uk (F.C.W.)
http://dx.doi.org/10.1016/j.celrep.2017.06.011

SUMMARY

T-box transcription factors T/Brachyury homolog A (Ta) and Tbx16 are essential for correct mesoderm development in zebrafish. The downstream transcriptional networks guiding their functional activities are poorly understood. Additionally, important contributions elsewhere are likely masked due to redundancy. Here, we exploit functional genomic strategies to identify Ta and Tbx16 targets in early embryogenesis. Surprisingly, we discovered they not only activate mesodermal gene expression but also redundantly regulate key endodermal determinants, leading to substantial loss of endoderm in double mutants. To further explore the gene regulatory networks (GRNs) governing endoderm formation, we identified targets of Ta/Tbx16-regulated homeodomain transcription factor Mixl1, which is absolutely required in zebrafish for endoderm formation. Interestingly, we find many endodermal determinants coordinately regulated through common genomic occupancy by Mixl1, Eomesa, Smad2, Nanog, Mxtx2, and Pou5f3. Collectively, these findings augment the endoderm GRN and reveal a panel of target genes underlying the Ta, Tbx16, and Mixl1 mutant phenotypes.

INTRODUCTION

The primary germ layers of the vertebrate embryo—endoderm, mesoderm, and ectoderm—are specified early in development. Endoderm derivatives contribute to liver, pancreas, gut tube, and respiratory tract, whereas mesoderm gives rise to muscle, connective tissues, and blood. The transforming growth factor β (TGF-β) family growth factor Nodal is required for formation of bipotential precursors of mesoderm and endoderm—the mesendoderm (Schier, 2009). On pathway activation, its downstream effectors, transcription factors (TFs) Smad2/3, translocate into the nucleus and interact with other TFs, such as the T-box TF Eomes, to activate expression of mesendodermal target genes.

T-box TFs play key roles in mesoderm and endoderm formation. For example, in mouse, Eomesodermin (Eomes) is required for definitive endoderm formation (Arnold et al., 2008), whereas zebrafish Eomes homolog A (Eomesa) regulates early endoderm marker expression (Du et al., 2012). T is required for normal mesoderm formation, with notochord and posterior somites failing to differentiate in mutant mice (Dobrovolskaïa-Zavadskaïa, 1927). In zebrafish, Ta is also required for notochord formation and it acts synergistically with its paralog, Tb, in posterior somite formation (Halpern et al., 1993; Martin and Kimelman, 2008; Schulte-Merker et al., 1994). Another T-box TF, Tbx16, plays a key role in zebrafish mesoderm formation though directing migration of mesodermal progenitors during gastrulation (Ho and Kane, 1990). Both Ta and Tbx16 regulate fibroblast growth factor (FGF) and Wnt signaling to control intermediate mesoderm formation and somitogenesis (Kimelman, 2016; Mueller et al., 2010; Warga et al., 2013) and have independent and combinatorial roles in establishing left-right asymmetry (Amack et al., 2007). Indeed, T-box TFs often share partially overlapping functions. For example, in *Xenopus* T, Eomes and VegT (ortholog of Tbx16; Griffin et al., 1998) redundantly regulate neuromesodermal bipotency (Gentsch et al., 2013).

This study focuses on transcriptional networks directed by Ta and Tbx16 in early zebrafish development. We characterized their DNA-binding activities and target gene expression profiles during gastrulation. We discovered that Ta/Tbx16 genomic binding substantially overlaps and provide evidence that use of common *cis*-regulatory modules (CRMs) accounts for their functional redundancy (Garnett et al., 2009). Here, we describe a profound loss of endoderm in *ta/tbx16* double mutants and present findings demonstrating that Ta/Tbx16 directly regulate the cell-intrinsic endodermal regulator Mixl1 (Kikuchi et al., 2000), as well as extrinsic regulators of endoderm proliferation, the Cxcr4a ligands Cxcl12a/b (Mizoguchi et al., 2008; Stückemann et al., 2012).

To understand how transcriptional programs downstream of Ta and Tbx16 control endoderm formation, we assessed Mixl1

 © 2017 The Author(s).
This is an open access article under the CC BY license (http://creativecommons.org/licenses/by/4.0/).

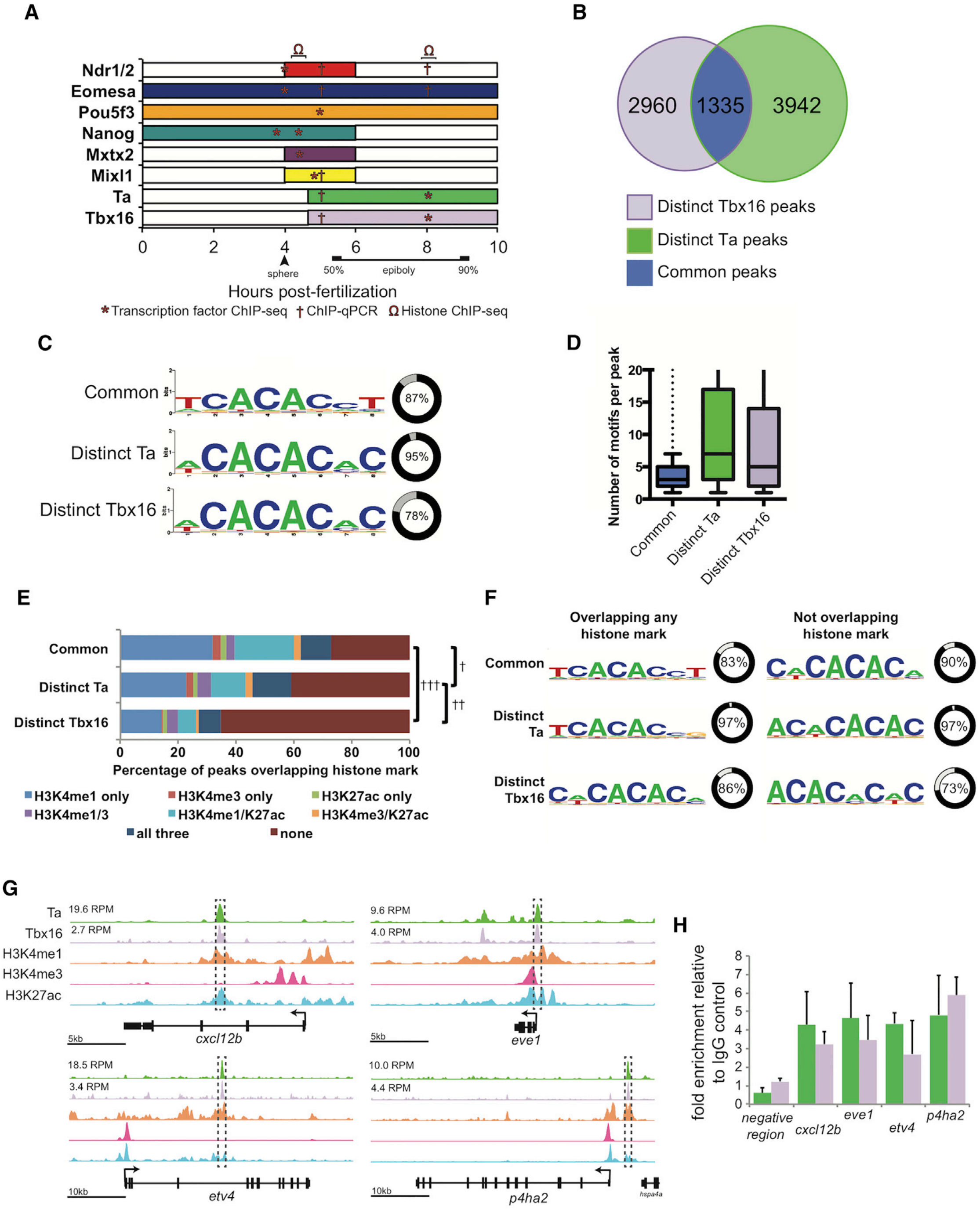

Figure 1. Genome-wide Analysis of Ta and Tbx16 Binding Sites

(A) Summary of the expression of the endodermal regulators (or their upstream activator) for which ChIP data are presented. Bars indicate the temporal expression window of factors at the margin, color coded per factor as in subsequent figures. Datasets indicated are ChIP-seq: Smad2 (regulated by Ndr1/2) and Eomesa at 3.3–4 hpf; Nanog and Mxtx2 at 3.3 and 4.3 hpf; Pou5f3 at 5 hpf; Mixl1 at 4.7–5.3 hpf; Ta and Tbx16 at 8–8.5 hpf; and histones at 8.25 hpf. ChIP-qPCR are Smad2, Eomesa, Mixl1, Ta, and Tbx16 at 5.3 hpf and Ta and Tbx16 at 8–8.5 hpf.

(B) Overlap of Ta and Tbx16 ChIP-seq peaks at 75%–85% epiboly (8–8.5 hpf).

(C) Closest match to the consensus T-box binding site identified within each peak class. Percentage of peaks containing such a sequence is indicated.

(D) Occurrences of motifs indicated in (C) within each peak of each class. Boxplots intervals are 10th, 25th, median, 75th, and 90th percentiles.

(E) Percentage of peaks in each class overlapping histone marks. †p = 3×10^{-19}; ††p = 4×10^{-89}; †††p = 9×10^{-119}, chi-square test. See also Figure S1.

(legend continued on next page)

genomic binding during endoderm specification, revealing direct regulation of many key endoderm-intrinsic factors via CRM occupancy with Smad2 and Eomesa. Moreover, we found Mixl1 binds common CRMs with key endodermal determinants Nanog, Mxtx2, and Pou5f3 (Leichsenring et al., 2013; Lunde et al., 2004; Reim et al., 2004; Xu et al., 2012). Collectively, our data refine the transcriptional hierarchy underlying endoderm formation in zebrafish and strongly suggest these TFs act combinatorially to regulate target gene expression.

RESULTS

Genome-wide ChIP-Seq Analysis of Ta and Tbx16 Binding in Zebrafish Gastrulae

To study the roles of Ta, Tbx16, and other TFs, we assessed DNA binding, histone modification, and Ta/Tbx16-dependent target gene expression profiles between zygotic genome activation and the end of gastrulation. Figure 1A shows time points for individual TF datasets and the temporal expression of these TFs at the margin (mesodermal and endodermal cells).

Ta and Tbx16 chromatin immunoprecipitation sequencing (ChIP-seq) at mid-gastrulation (75%–85% epiboly; 8–8.5 hr post-fertilization [hpf]) identified a similar number of binding events (ChIP-seq peaks; Data S1) for each TF. Of these, ~25%–30% overlap (Figure 1B; Data S1), which we designate “common” peaks, whereas peaks unique to each TF we designate “distinct”.

Previous studies demonstrated Ta and Tbx16 bind the T-box consensus sequence TCACACCT (Garnett et al., 2009; Kispert and Hermann, 1993; Morley et al., 2009); however, T-box TFs also bind AC-rich sequences at lower affinity (Evans et al., 2012). De novo motif analysis revealed 85% of common peaks contain close matches to the consensus T-box site, whereas distinct peaks are most enriched for AC-rich sequences (Figure 1C). Interestingly, common peaks contain few consensus T-box sites, whereas the distinct peaks contain numerous AC-rich sites (Figure 1D).

To determine whether there are differences in functionality between peak subsets, we compared our data with published histone ChIP-seq indicative of putative promoters (H3K4me3), putative enhancers (H3K4me1), and active enhancers (H3K27ac) (Bogdanovic et al., 2012). This genome-wide analysis revealed common peaks were significantly more likely to overlap these histone marks than distinct peaks, whereas distinct Ta peaks were more correlated with histone marks than distinct Tbx16 peaks (Figure 1E). Interestingly, whereas common peaks at histone marks are most enriched for the consensus T-box, common peaks lacking functional marks are most enriched for the AC-rich sequences (Figure 1F).

Putative target genes were then annotated (nearest transcription start site [TSS] ± 100 kb from each peak, though the majority are markedly closer; Figure S1). These, together with Ta and Tbx16 binding coordinates and associations with histone marks, are shown in Data S1. Among target genes with common Ta/Tbx16 peaks at functional chromatin are FGF target gene *etv4* (Roehl and Nüsslein-Volhard, 2001), mesodermally expressed endodermal regulator *cxcl12b* (Mizoguchi et al., 2008; Nair and Schilling, 2008; Stückemann et al., 2012), mesodermal progenitor regulator *eve1* (Seebald and Szeto, 2011), and migration-associated marker *ph4a2* (Chang et al., 2011; Figures 1G and 1H), all of which play roles in key Ta and Tbx16 activities.

Expression Profiling of Ta and Tbx16 Target Genes

Expression of target genes with common peaks was significantly enriched in tissues co-expressing Ta and Tbx16, such as the margin and tailbud (Figure 2A; Data S2). Similarly, gene ontology (GO) term analysis demonstrates enrichment for functions common to both TFs (Figure 2B; Data S2). Interestingly, genes with distinct Ta, but not Tbx16 peaks, show enriched expression in the axial chorda mesoderm and notochord, where *ta*, but not *tbx16*, is expressed (Figures 2A and 2C). ChIP-qPCR validation confirms Ta-specific binding at axial chorda mesoderm genes *col8a1a*, *dmd*, and *itga6* (Figure 2D). Thus, consistent with enrichment of distinct Ta peaks at functional chromatin marks (Figure 1E), these Ta-binding events seem to be cell type specific.

To further investigate functional redundancy, we examined microarray data for single or double knockdown (KD) of Ta and Tbx16 by validated morpholino (MO) injection, at the same developmental stage as our ChIP-seq data (Garnett et al., 2009). As judged by gene set enrichment analysis (GSEA), Ta/Tbx16 occupancy is not highly correlated with changes in target gene expression upon loss of either TF alone (Figures 2E and 2F). However, common binding is significantly correlated with changes on combinatorial loss of both TFs (Figures 2G–2I). This suggests that, whereas changes in target gene expression occur on loss of each individual TF, leading to the known mutant phenotypes, loss of both TFs leads to greater reduction of a subset of genes with Ta/Tbx16 binding at the same CRMs. These data extend earlier conclusions (Garnett et al., 2009), suggesting that target gene expression is controlled through common rather than distinct CRMs.

Ta and Tbx16, Together with Eomesa and Smad2, Cooperatively Control Expression of Key Endodermal Regulators

If co-expressed T-box TFs redundantly regulate a subset of targets via common CRMs, do Ta and Tbx16 share targets with Eomesa? We compared our Ta and Tbx16 ChIP-seq data with high-sphere stage (3.3–4 hpf) Eomesa and Smad2 ChIP-seq data (Nelson et al., 2014). Whereas these data are from a different developmental stage, they allowed us to test whether Ta/Tbx16/Eomesa ever occupy common CRMs. This revealed

(F) Closest match to the canonical T-box binding site identified within each class of peak overlapping histone marks with percentage of peaks containing such sequences indicated.

(G) Stage-matched Ta, Tbx16, H3K4me1, H3K4me3, and H3K27ac and ChIP-seq at the various genomic loci. Peak heights in reads per million (RPM) are indicated. Boxed regions indicate regions used for ChIP-qPCR validation.

(H) ChIP-qPCR validation of regions indicated in (G). Data are represented as mean ± SEM.

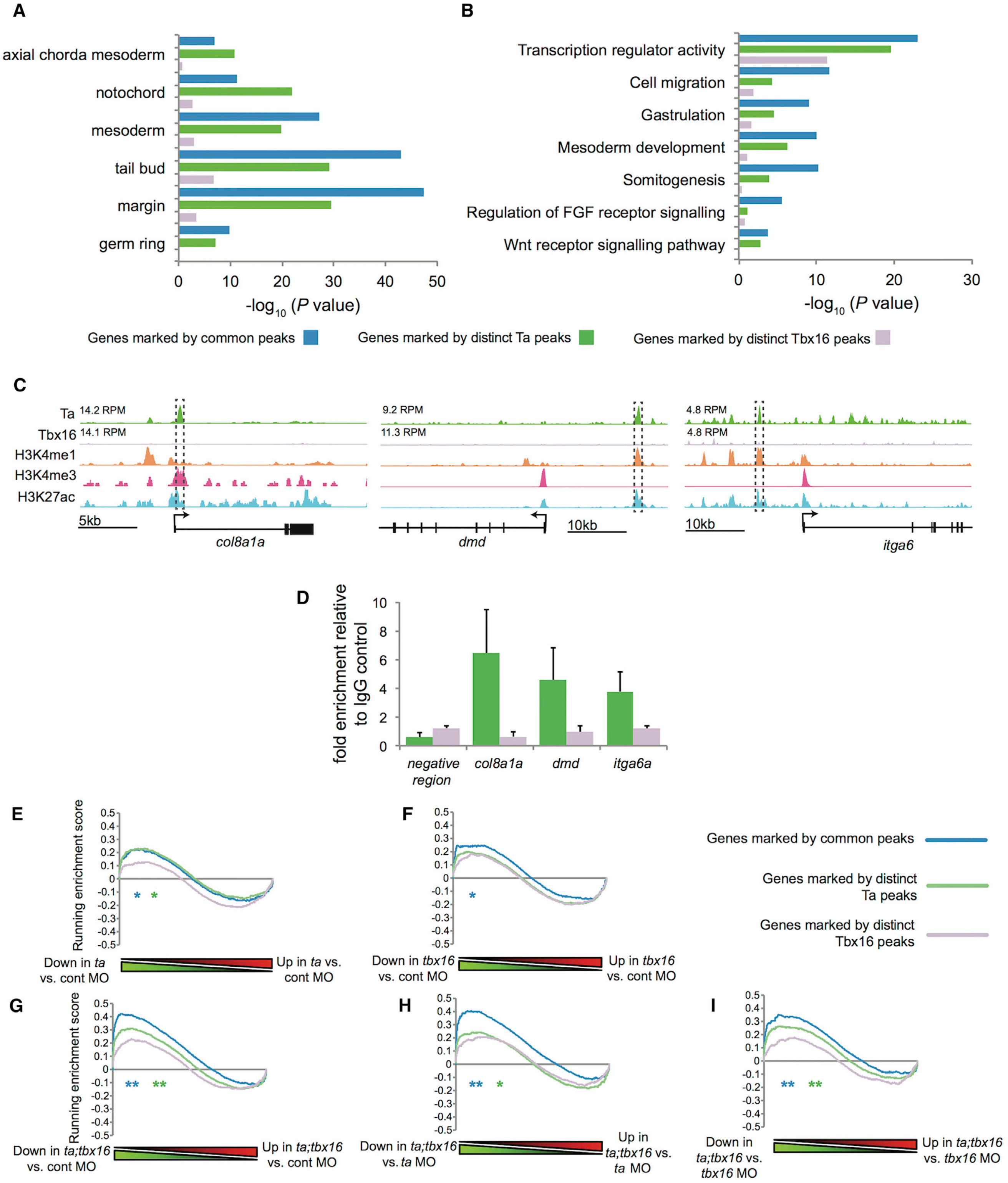

Figure 2. Ta and Tbx16 Show Cell-Type-Specific Binding Profiles and Redundantly Regulate Genes Showing Common Occupancy of Both Factors

(A) Enrichment for target genes with distinct Ta, Tbx16, or common binding (as indicated in Figure 1B) expressed within cell types where *ta* and/or *tbx16* are expressed, as defined by the ZFIN database (http://www.zfin.org; Howe et al., 2013). Blue, common peaks; green, distinct Ta peaks; purple, distinct Tbx16 peaks.

(legend continued on next page)

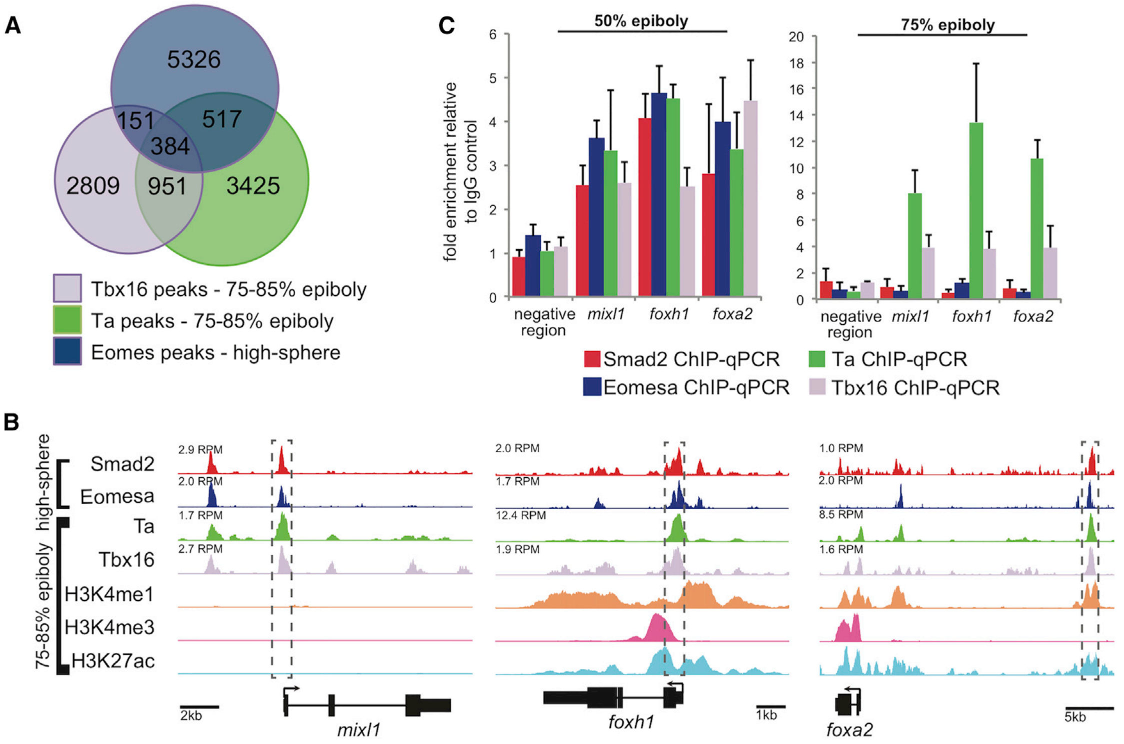

Figure 3. Comparison of Ta/Tbx16 Genomic Occupancy with Eomesa/Smad2 Reveals Direct Regulation of Endodermal Determinants prior to Gastrulation

(A) Overlap of Ta and Tbx16 ChIP-seq peaks at 75%–85% epiboly (8–8.5 hpf) with Eomesa at high-sphere stage (3.3–4 hpf).

(B) Smad2, Eomesa, Ta, Tbx16, H3K4me1, H3K4me3, and H3K27ac ChIP-seq peaks at indicated stages proximal to *mixl1*, *foxh1*, and *foxa2*. Peak heights in RPM are indicated. Boxed regions indicate peaks used for ChIP-qPCR validation.

(C) ChIP-qPCR analysis of regions indicated in (B) at the indicated stages. Data are represented as mean ± SEM.

a subset of such sites, located near key genes regulating endoderm formation, such as *mixl1*, *foxh1*, and *foxa2* (Kikuchi et al., 2000; Nelson et al., 2014; Shin et al., 2008; Slagle et al., 2011; Figures 3A and 3B; Data S3). Ta and Tbx16 binding proximal to *foxh1* and *foxa2* correlates with functional chromatin marks at 80% epiboly (8.25 hpf; Figure 3B). *Mixl1* expression is restricted to earlier stages during endoderm specification (4–6 hpf; Kikuchi et al., 2000); hence, at 80% epiboly, *mixl1* lacks such chromatin marks (Figure 3B). However, we observe Tbx16 and Ta (as well as Eomesa and Smad2) binding by qPCR proximal to *mixl1* at 50% epiboly (5.3 hpf; Figure 3C), coincident with endoderm specification; thus, Ta and Tbx16 may positively regulate *mixl1* expression at this earlier stage.

We also found Ta/Tbx16 binding proximal to *gata5* (Figure S2). To test whether Ta and Tbx16 are required for expression of *gata5*, *mixl1*, and their downstream target *sox32* (Bjornson et al., 2005), we examined *ta*, *tbx16*, and double morphants at 50% epiboly (5.3 hpf). Expression was weakly downregulated on *tbx16* KD and noticeably reduced on double KD (Figure 4A). Whole-mount in situ hybridization (WISH) revealed *sox32* expression was severely compromised in endoderm of double morphants, but not yolk syncytial layer (YSL), a region lacking *ta* and *tbx16* expression (Figure 4B). Thus, specification of endoderm progenitors requires both Ta and Tbx16, with Tbx16 having the greater effect.

Mesoderm-expressed chemokines *cxcl12a/b* were downregulated on *ta/tbx16* KD at 75% epiboly (8 hpf; Garnett et al., 2009) and our microarray dataset at 90% epiboly (9 hpf; Figure 4C; Data S4), demonstrating both *cxcl12a/b* are targets of Ta and Tbx16 (Figure 1G; Data S1). Thus, Ta and Tbx16 also regulate cell-extrinsic signaling cues driving proliferation of endodermal progenitors.

To evaluate endoderm expansion, we assayed expression of endoderm marker *sox17* at 90% epiboly (9 hpf). Immunodetection

(B) Bar graph showing enrichment for Gene Ontology terms associated with target genes with distinct or common binding (as indicated in Figure 1B).

(C) Stage-matched Ta, Tbx16, H3K4me1, H3K4me3, and H3K27ac and ChIP-seq profiles. Peak heights in RPM are indicated. Boxed regions indicate peaks used for ChIP-qPCR validation.

(D) Ta (green) and Tbx16 (mauve) ChIP-qPCR validation of regions indicated in (C). Data are represented as mean ± SEM.

(E–I) GSEA enrichment plots for comparison of target genes with distinct or common binding (as indicated in Figure 1B) with (E) *ta* KD relative to control; (F) *tbx16* KD relative to control; (G) *ta/tbx16* double KD relative to control; (H) *ta/tbx16* double KD relative to *ta* KD; (I) *ta/tbx16* double KD relative to *tbx16* KD. *Family-wise error rate (FWER) $p \leq 3 \times 10^{-2}$; **FWER $p \leq 5 \times 10^{-4}$.

OPEN
ACCESS
CellPress

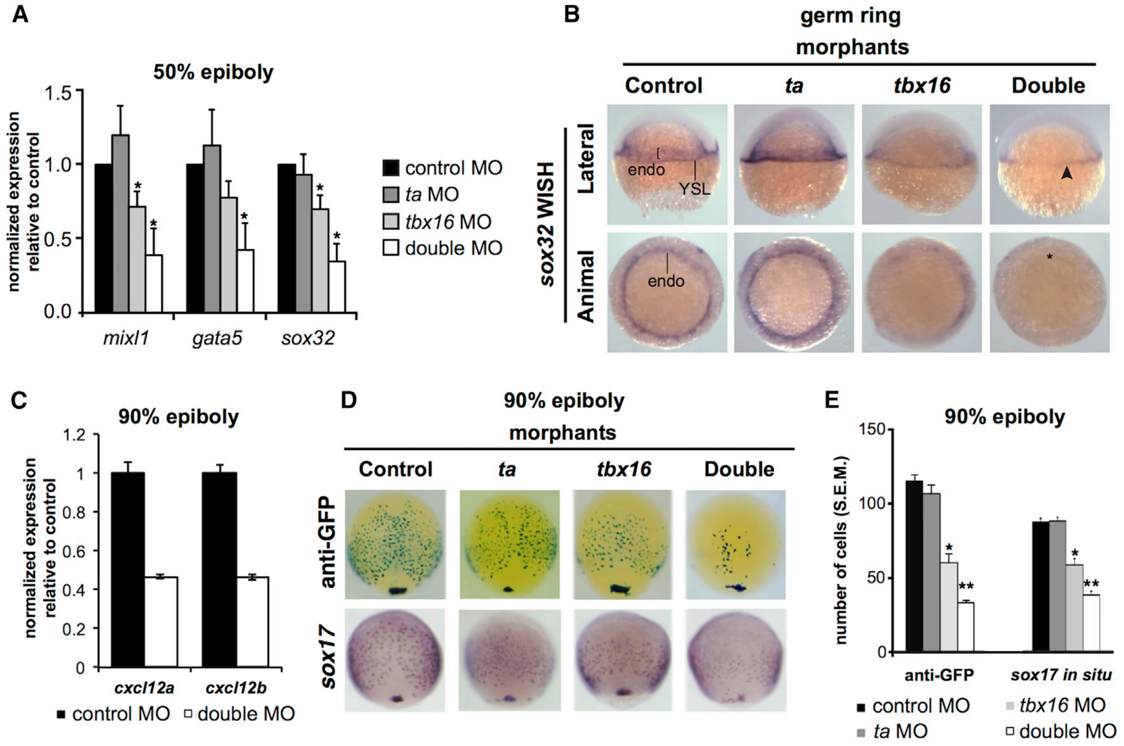

Figure 4. Loss of Ta and Tbx16 Leads to Downregulation of Endodermal Specifiers and Reduction of Endodermal Progenitors during Gastrulation

(A) qPCR analysis of *mixl1*, *gata5*, and *sox32* at 50% epiboly (5.3 hpf) in single and double *ta/tbx16* morphants. All genes are significantly downregulated in double morphants; $*p \leq 5 \times 10^{-2}$; Student's t test. Data are represented as mean ± SEM. See also Figure S2.

(B) WISH analysis of *sox32* in single and double morphants at germ ring stage (5.7 hpf). Arrowhead, YSL expression; *, loss of endoderm expression.

(C) Microarray analysis at 90% epiboly (9 hpf) indicates downregulation of *cxcl12a/b*. Data are represented as mean ± SEM.

(D) Immunological and WISH analysis of a *sox17:eGFP* transgene and endogenous *sox17* expression at 90% epiboly (9 hpf) in single and double morphants.

(E) Cell numbers identified by immunostaining and WISH in (D). Cell numbers are representative of at least 20 embryos per condition. $*p \leq 1 \times 10^{-8}$; $**p \leq 1 \times 10^{-20}$; Student's t test. Data are represented as mean ± SEM.

See also Figure S3.

of a *sox17:eGFP* transgene and *sox17* WISH revealed endoderm cell numbers moderately reduced by *tbx16* KD and substantially reduced on *ta/tbx16* double KD (Figures 4D and 4E), strongly suggesting Ta and Tbx16 co-operatively promote both specification and expansion of the endoderm progenitor niche. In contrast, the *ta* paralog *tb* does not interact with *ta* in early endoderm formation because neither single nor *ta/tb* double KD significantly affected *sox17*+ cell numbers at 90% epiboly (9 hpf; Figures S3A and S3B).

Ta and Tbx16 Are Required for Correct Liver, Gut, and Pancreas Formation

If Ta and Tbx16 regulate endoderm specification and proliferation, we would expect gut and associated organs to form abnormally. Examination of *sox17:eGFP* at 24 hpf on double KD revealed the gut tube was severely compromised (Figure 5A), although pharyngeal endoderm remained intact. WISH analysis of broad endoderm (*foxa3*), pancreas (*ins*), and liver (*cp*) markers at 52–56 hpf revealed disordered liver and pancreas formation, such as laterality defects, in both *ta* and *tbx16* morphants (Amack et al., 2007; Danos and Yost, 1996), whereas Tbx16 morphants also display some loss of *ins* and *cp* expression. Double Ta/Tbx16 morphants, however, substantially lack *foxa3*, *ins*, and *cp* expression (Figures 5A and 5B). Double KD of Tb with Ta led to laterality defects (Figures S3C and S3D). Examination of $ta^{+/-}$ and $tbx16^{+/-}$ intercrosses revealed similar phenotypes to single Ta or Tbx16 morphants, whereas $ta^{+/-};tbx16^{+/-}$ intercross embryos also gave phenotypes similar to Ta/Tbx16 double KD, at expected Mendelian ratios (Figures 5C–5E); thus, our observations are not artifacts of MO injection. Interestingly, intercrosses of $ta^{+/-};tbx16^{+/-}$ with $tbx16^{+/-}$ animals (*tbx16* enhanced) revealed that one wild-type *ta* allele is insufficient to rescue loss of *foxa3*, *cp*, and *ins* expression. In contrast, intercrosses of $ta^{+/-};tbx16^{+/-}$ with $ta^{+/-}$ animals (*ta* enhanced) that maintain a wild-type *tbx16* allele did not show significant loss of *ins* and *cp* expression, indicating Tbx16 has the greater influence on endoderm formation (Figures 5C–5E).

We conclude that Ta and Tbx16 play essential roles in endoderm progenitors, acting cell-autonomously via Mixl1 and non-autonomously governing cell-extrinsic Cxcl12a/b signaling pathways. Moreover, Ta and Tbx16 are redundantly required for correct gut, liver, and pancreas formation.

Mixl1 Acting Downstream of Ta and Tbx16 Governs Nodal/Smad Target Gene Expression

To understand how transcriptional programs downstream of Ta and Tbx16 control endoderm formation, we next investigated Mixl1 target genes during endoderm specification by ChIP-seq at 30%–50% epiboly (4.7–5.3 hpf; Figure S4). De novo motif analysis identified a sequence closely resembling the previously described consensus binding motif within our ChIP-seq peaks (Zhang et al., 2009; Figure 6A).

We previously showed Smad2 and Eomesa bind CRMs proximal to Nodal-responsive genes (Nelson et al., 2014), and Mixl1 is known to physically interact with Smad2 at Nodal-responsive CRMs (Germain et al., 2000). Consistent with this, we observe Mixl1 occupancy at the same CRMs as Eomesa and Smad2 proximal to key regulators of endoderm formation (Figure 6B; Data S3). Targets with overlapping Eomesa, Smad2, and Mixl1 ChIP-seq peaks display expression domains co-localized with Nodal activity, further suggesting Mixl1 regulates similar Nodal targets to Eomesa/Smad2 (Figure 6C; Data S5). Moreover, common occupancy of Mixl1/Smad2/Eomesa at endoderm target genes is associated with functional chromatin marks at dome stage (4.3 hpf; Figure 6D). To confirm occupancy of Eomesa, Smad2, and Mixl1 at common target sites during endoderm specification, we performed ChIP-qPCR at 50% epiboly (5.3 hpf; Figure 6E). Comparison of our ChIP-seq data with microarray data on overexpression of the Nodal ligand *ndr1* in zebrafish blastulae (3.5–4 hpf; Nelson et al., 2014) revealed highly significant association between upregulated genes and binding of Mixl1 with Smad2 and/or Eomesa (Figure 6F). Genes with Mixl1 binding at the same CRMs as Eomesa and/or Smad2 are therefore induced by Ndr1, strongly suggesting Mixl1 co-operatively regulates Nodal target genes in association with Eomesa and Smad2. Intriguingly, 20 Ndr1-induced genes with proximal Mixl1/Smad2/Eomesa binding, including *gata5*, *gsc*, *wnt8a*, and *fgf8a* were also downregulated on Ta/Tbx16 double KD at shield stage (6 hpf; Figures 6G and 6H; Data S6). This suggests Ta and Tbx16 influence a subset of Nodal targets and that this may be partially due to their regulation of *mixl1*.

Characterization of the Endodermal Gene Regulatory Network through Comparison of Mixl1, Smad2, Eomesa, Nanog, Mxtx2, and Pou5f3 Occupancy

Because TFs Nanog, Mxtx2, and Pou5f3 also play known roles in endoderm formation in the blastula embryo (Lunde et al., 2004; Reim et al., 2004; Xu et al., 2012), we compared our Mixl1, Smad2, and Eomesa ChIP-seq data with that for Nanog, Mxtx2 (both 3.3 and 4.3 hpf), and Pou5f3 (5 hpf; Leichsenring et al., 2013; Xu et al., 2012). At high stage (3.3 hpf), although only a minority of Nanog peaks overlap with Smad2 and/or Eomesa, we found these common ChIP-seq peaks proximal to key endodermal regulators, such as *ndr1*, *gata5*, *sox32*, and *tbx16* (Figure 7A). Similarly, at dome-50% epiboly (4.3–5 hpf), despite limited Nanog, Mxtx2, Pou5f3, and Mixl1 peak overlap, these TFs display common binding at CRMs proximal to key endodermal regulators (Figure 7B; Data S7). Importantly, genes exhibiting proximal binding of multiple TFs were highly enriched for relevant developmental functions and expression patterns. Genes with proximal binding of individual TFs alone were notably less enriched for such terms (Figures S5 and S6), strongly suggesting that these four TFs perform their developmental roles in combination (Data S5 and S7).

Our analyses suggest the transcriptional processes underlying endoderm formation are complex, requiring coordinated temporal regulation of vital target genes by combinations of numerous TFs. An updated gene regulatory network (GRN) for endoderm formation incorporating this study is in Figure 7C.

DISCUSSION

The combinatorial requirement for Ta and Tbx16 in trunk and tail mesoderm formation has been known for 15 years (Amacher et al., 2002). Here, we report their redundant role in endoderm formation. Through characterizing genome-wide binding profiles of these TFs, we have identified a set of common target CRMs and regulated genes that can account for the action of Ta and Tbx16 in formation of mesoderm and endoderm. We find that Ta and Tbx16 bind and regulate *mixl1* expression, which is required for endoderm formation (mutated in *bonnie and clyde*; Kikuchi et al., 2000), suggesting that Ta and Tbx16 influence endoderm formation via Mixl1. To better understand how Mixl1 controls endoderm formation, we profiled its genomic binding and integrated these data with existing Eomesa, Smad2, Nanog, Mxtx2, and Pou5f3 early embryo datasets to present an augmented GRN for zebrafish endoderm formation.

High-Affinity Binding of Ta and Tbx16 at Functional CRMs

Our data show that Ta and Tbx16 are able to bind a subset of the same CRMs. These common peaks are enriched for low numbers of consensus T-box motifs overlapping functional chromatin marks and are correlated with genes that mediate Ta and Tbx16 function. Conversely, CRMs bound by either Ta or Tbx16 only (distinct peaks) contain AC-rich sequences, as do common peaks that do not overlap functional chromatin. Previous work suggests that T-box TFs bind AC-rich sites with low affinity but the consensus site with high affinity (Evans et al., 2012). Our data therefore support the idea that the high-affinity sites are more efficiently bound and are important in targeting T-box TFs to functional CRMs.

Although Ta and Tbx16 bind common CRMs in vivo, whether they simultaneously bind the same CRM in the same cells or bind independently is unclear. Whereas our whole-embryo ChIP data cannot distinguish between these two possibilities, our previous in vitro electromobility shift analysis of a CRM controlling *dlc* expression suggests that, although four spatially distinct T-box sites are present, the CRM is only occupied by Ta or Tbx16 individually (Jahangiri et al., 2012). Whether this is also the case in vivo or at other CRMs remains to be determined. However, we note some tissue-specific binding may be detected in our data because Ta, which is expressed in notochord, binds CRMs in the vicinity of notochord genes, whereas Tbx16, which is not expressed in the notochord, does not bind these regions.

Ta and Tbx16 in Formation of Mesendoderm

Through comparison of our Ta/Tbx16-binding data with that of maternal T-box TF Eomesa, which is involved in mesoderm

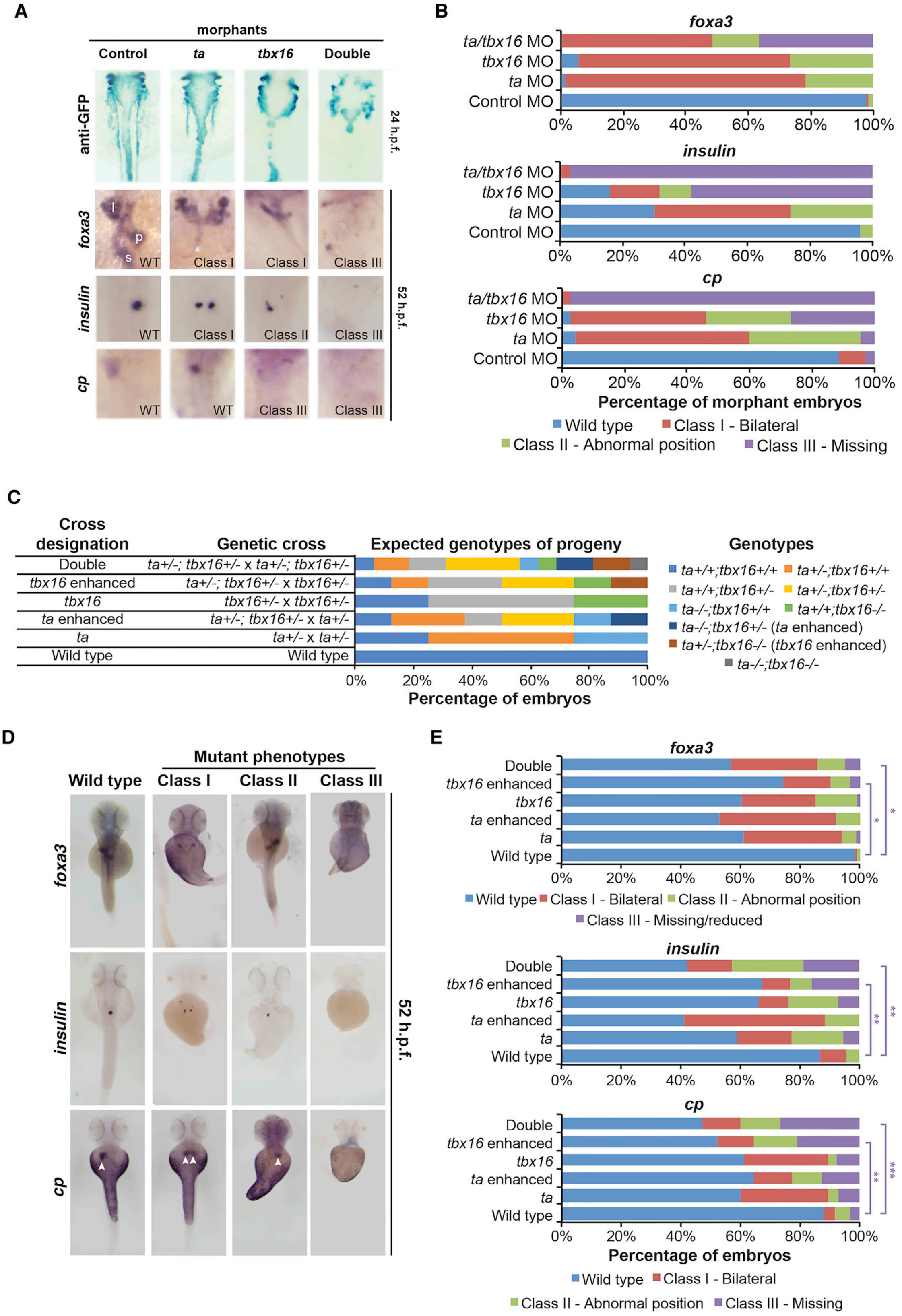

(legend on next page)

and endoderm formation, we discovered all three TFs bind an overlapping set of CRMs, including those associated with endodermal genes, such as *mixl1*. This led us to ask whether Ta and Tbx16 are also involved in endoderm formation. Indeed, we show that embryos with reduced Ta and Tbx16 activity have reduced *mixl1* expression, less endoderm at the end of gastrulation, and consequently fail to form a complete gut and associated organs, the pancreas and liver.

In zebrafish, mesendoderm formation requires Nodal signaling (Schier, 2009), whereas FGF is required for correct mesoderm formation, in part via activation of *ta* and *tbx16*, and by antagonizing endoderm formation through phosphorylation and inactivation of Sox32 (Poulain et al., 2006). Nodal signaling, which is mediated by Smad2, is active in the first five or six cell tiers of the margin in the blastula embryo, whereas FGF activity extends further (van Boxtel et al., 2015), leading to the idea that cells closest to the margin become endoderm due to high levels of Nodal signaling, whereas endoderm fate is repressed by FGF further from the margin. During mesendoderm formation, Eomesa interacts with Smad2 and other TFs, including its downstream target Mixl1, to activate endodermal CRMs, although the requirement for Eomesa to activate endodermal genes is transient (Du et al., 2012). In response to Nodal, *ta* and *tbx16* are induced at the margin, coincident with endodermal genes, such as *gata5* (Rodaway et al., 1999) and *mixl1*.

Our data suggest that this expression of *ta* and *tbx16* at the margin prior to gastrulation is key to establishing *sox32* expression via upstream regulators, including *mixl1*, thus locking down endoderm fate, whereas presence of Ta and Tbx16 in cell tiers further from the margin lacking phospho-Smad2 would be insufficient to promote endoderm fate. It is also tempting to speculate that, with downregulation of Eomesa activity during blastula stages (Bruce et al., 2003), Ta and Tbx16 may target Smad2 to CRMs promoting endoderm fate. Consistent with this, we show co-occupancy of Ta/Tbx16/Smad2 at such elements. This idea is also supported by the observation that Mixl1 overexpression can only induce endoderm at the margin (Bjornson et al., 2005), where Ta and Tbx16 are expressed.

At the onset of gastrulation, *ta* and *tbx16* (along with their target *mixl1*) are rapidly downregulated in endoderm. At this stage, they appear to act in mesoderm to control *cxcl12a/b* expression, thus non-autonomously promoting correct endoderm migration and proliferation. Downstream endoderm lineages are therefore likely lost in mutants due to a diminished endoderm progenitor pool.

The function of Ta/Tbx16 in mesoderm may be in part independent of Nodal because Smad2 is absent from mesodermal progenitors further from the margin but may instead rely on interaction with BMP-regulated Smads, such as Smad1 (Faial et al., 2015; Messenger et al., 2005), as well as Smad-independent mechanisms.

Conserved and Divergent Functions for T-Box Factors

T is highly conserved in sequence and function across all vertebrates, being required for notochord and posterior mesoderm formation (Naiche et al., 2005). Although not required, a role for T in endoderm formation may also be conserved. During mammalian stem cell differentiation, cells expressing T go on to form either mesoderm or endoderm, and T has been implicated in endoderm formation in mouse and human in vitro through binding endodermal CRMs (Faial et al., 2015; Lolas et al., 2014). In addition, in the presence of Smad2, with which it physically interacts, T is able to induce endoderm markers in differentiating human cells (Faial et al., 2015).

A role in endoderm formation for *tbx16* orthologs may also be conserved. For instance, the *Xenopus* ortholog of *tbx16*, *vegt* (Griffin et al., 1998), is required for endoderm formation (Zhang et al., 1998). Despite VegT being maternally contributed and *eomes* zygotically expressed (Showell et al., 2004)—the opposite of zebrafish where Tbx16 is zygotic and Eomesa maternal—the importance of Tbx16 to zebrafish endoderm formation highlights a clear parallel with *Xenopus*.

In mouse, of all T-box TFs, Eomes alone is required for endoderm formation (Arnold et al., 2008). It is interesting to note that *tbx16* orthologs are present in teleost, amphibian, and avian species but lost in mammals (Ahn et al., 2012); thus, it is possible that a key difference between zebrafish/*Xenopus* and mammals may be an increased dependency on Eomes due to loss of Tbx16/VegT.

An Augmented GRN for Zebrafish Endoderm Formation

Mixl1 is key to endoderm formation, with mutants failing to form the vast majority of endoderm (Kikuchi et al., 2000), though its direct target genes in zebrafish were unknown aside from *sox32* (Bjornson et al., 2005). We identified a panel of candidate Mixl1 target genes sufficient to explain the loss of endoderm in *mixl1* mutants, such as *pou5f3* and *gata5*, acting upstream of *sox32* (Kikuchi et al., 2001; Lunde et al., 2004), as well as *sox32* itself.

Previous understanding of the GRN controlling early zebrafish endoderm formation involved maternally contributed TFs, including Eomesa and Nanog, combining to induce YSL formation via Mxtx2, leading to Nodal production. Along with other maternal TFs, such as Pou5f3 and Smad2, they also control

Figure 5. Ta and Tbx16 Are Redundantly Required for Liver, Pancreas, and Gut Development

(A) GFP immunostaining in single and double morphant *sox17:eGFP* transgenic fish at 24 hpf and WISH analysis of broad endodermal organ marker *foxa3*, pancreas marker *ins*, and liver marker *cp* at 52–56 hpf in single and double morphants. l, liver; p, pancreas; s, stomach. Phenotypic classes as defined in (B) are indicated.

(B) Percentage of KD embryos in each phenotypic class identified by WISH. Compare with Figure S3D. Graphs represent 19–124 embryos per group.

(C) Genetic crosses and expected embryonic genotypes. *Ta*-enhanced ($ta^{-/-}$;$tbx16^{+/-}$) and *tbx16*-enhanced ($ta^{+/-}$;$tbx16^{-/-}$) genotypes are indicated.

(D) Phenotypic classes of embryos from genetic crosses indicated in (C) identified by WISH. Arrowheads indicate liver *cp* staining.

(E) Percentage of embryos in each phenotypic class from each genetic cross identified by WISH. Graphs represent 31–175 embryos per group. *$p \leq 3 \times 10^{-2}$; **$p \leq 5 \times 10^{-3}$; ***$p \leq 1 \times 10^{-4}$; all other comparisons with wild-type are not significant (p = 0.1–1); Fisher's exact test.

See also Figure S3.

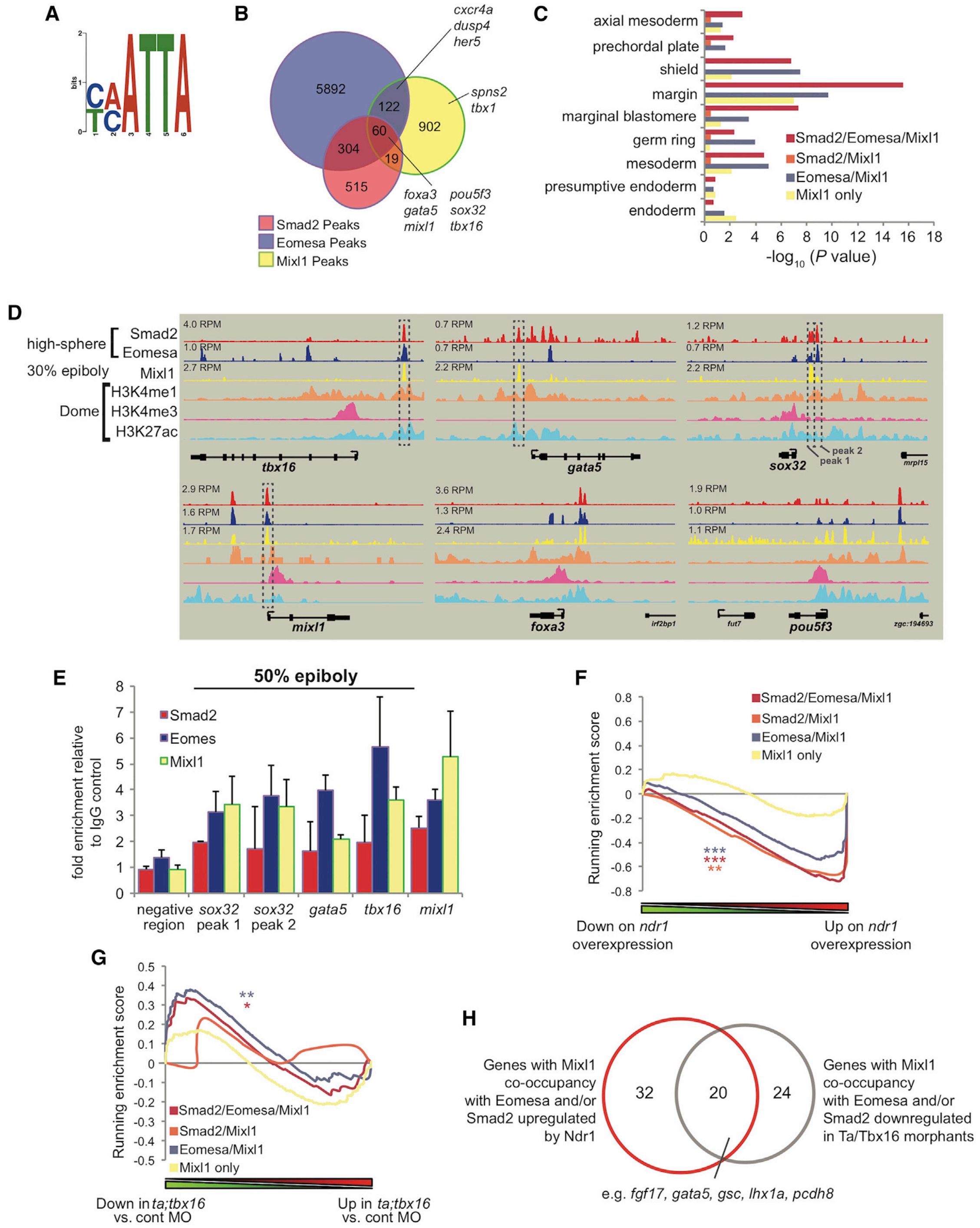

Figure 6. Mixl1 Occupies the Same Sites as Smad2 and Eomesa Proximal to Nodal-Responsive Endodermal Genes

(A) Motif identified within Mixl1 ChIP-seq peaks using DREME; e = 2.7×10^{-19}; p = 7.1×10^{-24}.

(B) Overlap of Eomesa and Smad2 ChIP-seq peaks at high-sphere stage (3.3–4 hpf) with Mixl1 peaks at 30%–50% epiboly (4.7–5.3 hpf). Endodermal regulators with occupancy of TFs are indicated.

(C) Enrichment for genes with Eomesa and/or Smad2 and/or Mixl1 proximal binding (as indicated in B). The graph shows enrichment for cell types where *ndr1* and/or *ndr2* are expressed.

(D) Smad2, Eomesa, Mixl1, H3K4me1, H3K4me3, and H3K27ac ChIP-seq at indicated stages proximal to *tbx16*, *gata5*, *sox32*, *mixl1*, *foxa3*, and *pou5f3*. Peak heights in reads per million (RPM) are indicated. Boxed regions indicate peaks used for ChIP-qPCR validation.

(E) ChIP-qPCR analysis of regions indicated in (D) at 50% epiboly (5.3 hpf). Data are represented as mean ± SEM.

(legend continued on next page)

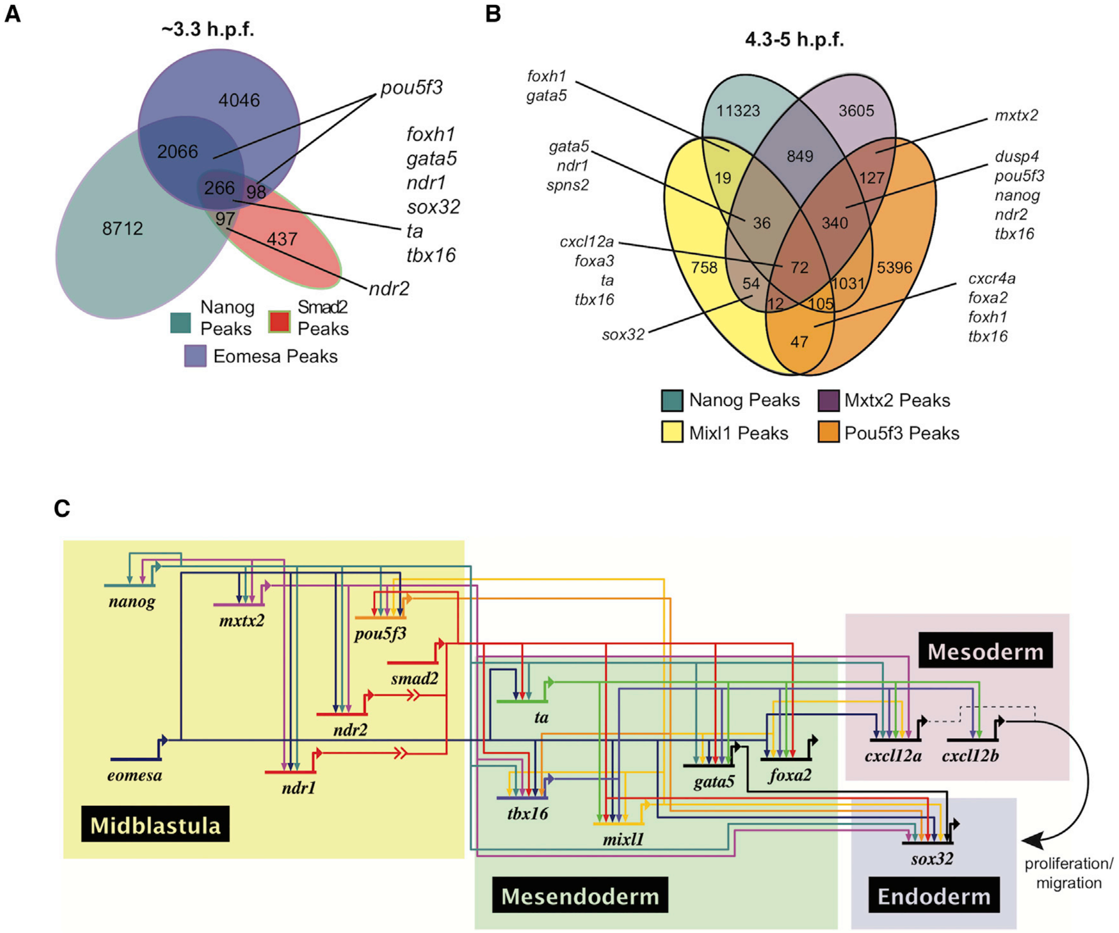

Figure 7. Nanog, Mxtx2, and Pou5f3 Occupy Sites Bound by Mixl1/Smad2/Eomesa Proximal to Key Endodermal Regulators

(A) Overlap of Nanog, Smad2, and Eomesa ChIP-seq peaks at high stage (3.3 hpf). Endodermal regulators with occupancy of TFs are indicated.

(B) Overlap of Nanog, Mxtx2, Pou5f3, and Mixl1 ChIP-seq peaks at 4.3–5 hpf. Endodermal regulators with occupancy of TFs are indicated.

(C) A GRN for endoderm formation informed by this study. Links within the network represent binding identified by ChIP plus expression change in this or cited studies. Illustrated boxes contain the following: "midblastula"—factors implicated in mesendoderm induction, a subset of which are maternally contributed; "mesendoderm"—TFs induced at the margin between onset of zygotic transcription and gastrulation, promoting endoderm formation; "endoderm"—master regulator of zebrafish endoderm formation Sox32, which ensures endoderm fate specification; and "mesoderm"—secreted chemokines induced in at the margin and expressed by mesoderm to promote endoderm proliferation and migration. >> indicates ligand-receptor binding, leading to Smad2 activation. Dotted line indicates the reported minor influence of Cxcl12a compared with Cxcl12b (Boldajipour et al., 2011).

See also Figures S5 and S6.

expression of key endoderm determinants, such as *gata5*, *mixl1*, and *sox32* in the emerging mesendoderm (Bjornson et al., 2005; Bruce et al., 2005; Dubrulle et al., 2015; Lunde et al., 2004; Reim et al., 2004; Xu et al., 2012). Combinations of these TFs positively regulate *sox32* expression, thus establishing endodermal fate. This study reveals roles for Ta and Tbx16 within this GRN, through regulation of *mixl1* and *cxcl12a/b*.

Intriguingly, our analyses also indicate that a subset of CRMs are bound by combinations of Eomesa, Smad2, Mixl1, Nanog, Mxtx2, and Pou5f3, including those proximal to other genes implicated in endoderm formation, such as *dusp4* (Brown et al., 2008), *cxcr4a* (Stückemann et al., 2012), and *spns2* (Osborne et al., 2008). It will be interesting to learn more about how these TFs collectively contribute to the function of the identified CRMs.

(F and G) GSEA plots of genes with proximal binding of Mixl1 alone or at the same CRMs as Eomesa and/or Smad2 (defined and color-coded as in B) compared with microarray data: (F) changes in expression on *ndr1* overexpression in blastulae—Mixl1 binding with Eomesa and/or Smad2 is highly correlated with genes induced by Ndr1; (G) changes in expression on *ta/tbx16* KD at shield (6 hpf)—Mixl1 binding with Eomesa and Smad2 is highly correlated with downregulated genes. *FWER p $\leq 2 \times 10^{-2}$; **FWER p $\leq 1 \times 10^{-3}$; ***FWER p $\leq 5 \times 10^{-4}$.

(H) Overlap of genes with occupancy of Mixl1 with Eomesa and/or Smad2 upregulated by Ndr1 (identified in F) or downregulated in Ta/Tbx16 morphants (identified in G).

See also Figure S4.

Whereas this study focused on endoderm formation, Ta, Tbx16, and Mixl1 also have key functions in mesoderm formation, which are represented in our data. We therefore provide a rich resource for future study, as well as adding additional players to the story of endoderm formation.

EXPERIMENTAL PROCEDURES

Details of immunohistochemistry, in situ hybridization, qRT-PCR, ChIP-qPCR, and cell counting are provided in Supplemental Experimental Procedures.

Animals

AB, *ta*$^{b195/+}$, *tbx16*$^{b104/+}$, and *ta*$^{b195/+}$;*tbx16*$^{b104/+}$ fish were reared as described (Westerfield, 2000). All zebrafish studies complied fully with the UK Animals (Scientific Procedures) Act 1986 as implemented by King's College London.

Morpholino Injection

One-cell stage embryos were injected with 0.5 pmol *tbx16* (Bisgrove et al., 2005) and 0.25 pmol *ta* (Feldman and Stemple, 2001), which recapitulate the mutant *ta* and *tbx16* phenotypes, respectively, or equivalent quantities of standard control MO (GeneTools).

ChIP-Seq and Data Analysis

For ChIP-seq, two independent replicate experiments were performed using 5,000 embryos each at the indicated developmental stage as described (Nelson et al., 2014) using previously characterized anti-Ta (Morley et al., 2009; Schulte-Merker et al., 1992) and anti-Tbx16 antibodies (Amacher et al., 2002; Garnett et al., 2009; Jahangiri et al., 2012) or a commercial anti-Mixl1 antibody (Anaspec 55613; Figure S4). Reads were mapped to the Zv9 zebrafish genome with Bowtie (Langmead et al., 2009) in Galaxy (Giardine et al., 2005; Goecks et al., 2010) using default parameters with the exceptions: -y -m2 -k2 –best (Table S1). We therefore used a maximum of two acceptable alignments, ensuring that best possible alignments were identified. Peak calling, relative to matched input samples, was performed using MACS (Zhang et al., 2008) with the parameters: Ta – m-fold 10, p value 1e−8; Tbx16 – m-fold 10, p value 1e−4; and Mixl1 – m-fold 10, p value 1e−5. For Ta ChIP-seq, one replicate gave lower signal-to-noise ratio. We therefore used the stronger replicate for further analyses. Key peak were validated by ChIP-qPCR (Figures 1H, 2D, and 3C). Peaks identified in both replicates are indicated in Data S1.

Histone ChIP-seq data were downloaded from NCBI GEO: GSE32483, mapped to the Zv9 genome as above, and peaks called using default MACS parameters with one exception (m-fold 20).

Other ChIP-seq data were downloaded from GEO: Nanog and Mxtx2, GEO: GSE34683; Pou5f3, GEO: GSE39780; and Eomesa and Smad2, GEO: GSE51894.

Peaks were associated with genes by annotating the nearest transcription start site ± 100 kb. Functional annotation analysis was performed using DAVID (Huang et al., 2009a, 2009b). De novo motif discovery was performed using Weeder (Pavesi et al., 2006) for Ta and Tbx16 peaks and DREME (Bailey, 2011) for Mixl1.

Microarray Experiments

Data for morphant embryos were generated at shield (6 hpf) and 90% epiboly (9 hpf) using Agilent Zebrafish Gene Expression Microarrays (V3) and analyzed as previously described (Nelson et al., 2014). Microarray data for control, *ta*, *tbx16*, and *ta*;*tbx16* double morphants at 75% epiboly (8 hpf; Garnett et al., 2009) were downloaded from GEO: GSE12857, cyclic loess normalized, and differential expression determined using the R package oneChannelGUI (Sanges et al., 2007). Data for *ndr1* overexpression in blastulae were previously described (Nelson et al., 2014; GEO: GSE51894).

For GSEA (Mootha et al., 2003; Subramanian et al., 2005), GSEA v2.2.2 was used applying 2,000 permutations to gene lists preranked on statistics obtained from microarray data analysis. To be sufficiently stringent when multiple gene sets were analyzed, the family-wise error rate (FWER) p value was used to establish significance.

Statistical Analysis

Quantitative data, including expression levels and cell counts, are expressed as mean ± SEM. Differences between groups were compared with a two-tailed Student's t distribution test. Differences in qualitatively scored phenotypes from mutant and wild-type matings or in knockdown experiments were compared using chi-square test. Differences in overlap of Ta and Tbx16 ChIP-seq peaks with histone marks were also compared using chi-square test. All tests were performed with a confidence level of 95%.

ACCESSION NUMBERS

The accession number for the microarray and ChIP-seq data reported in this paper is GEO: GSE84619.

SUPPLEMENTAL INFORMATION

Supplemental Information includes Supplemental Experimental Procedures, six figures, one table, and seven data files and can be found with this article online at http://dx.doi.org/10.1016/j.celrep.2017.06.011.

AUTHOR CONTRIBUTIONS

A.C.N. and F.C.W. conceived the study, performed the experiments, and wrote the manuscript; S.J.C., S.N.G., and I.D. performed the experiments; and D.L.S. participated in and facilitated acquisition of ChIP-seq data. All authors read and approved the final manuscript.

ACKNOWLEDGMENTS

This work was funded by an MRC Career Development Award (G0700213) and Lister Institute Research Prize to F.C.W. and a Wellcome Trust Programme grant (102811) to Elizabeth J. Robertson. We thank B. Draper and S. Schulte-Merker for reagents. We also thank E.K. Bikoff and E.J. Robertson for reagents, valuable conversation, and critical review of the manuscript.

Received: November 1, 2016
Revised: April 28, 2017
Accepted: May 31, 2017
Published: June 27, 2017

REFERENCES

Ahn, D., You, K.H., and Kim, C.H. (2012). Evolution of the tbx6/16 subfamily genes in vertebrates: insights from zebrafish. Mol. Biol. Evol. *29*, 3959–3983.

Amacher, S.L., Draper, B.W., Summers, B.R., and Kimmel, C.B. (2002). The zebrafish T-box genes no tail and spadetail are required for development of trunk and tail mesoderm and medial floor plate. Development *129*, 3311–3323.

Amack, J.D., Wang, X., and Yost, H.J. (2007). Two T-box genes play independent and cooperative roles to regulate morphogenesis of ciliated Kupffer's vesicle in zebrafish. Dev. Biol. *310*, 196–210.

Arnold, S.J., Hofmann, U.K., Bikoff, E.K., and Robertson, E.J. (2008). Pivotal roles for eomesodermin during axis formation, epithelium-to-mesenchyme transition and endoderm specification in the mouse. Development *135*, 501–511.

Bailey, T.L. (2011). DREME: motif discovery in transcription factor ChIP-seq data. Bioinformatics *27*, 1653–1659.

Bisgrove, B.W., Snarr, B.S., Emrazian, A., and Yost, H.J. (2005). Polaris and polycystin-2 in dorsal forerunner cells and Kupffer's vesicle are required for specification of the zebrafish left-right axis. Dev. Biol. *287*, 274–288.

Bjornson, C.R., Griffin, K.J., Farr, G.H., 3rd, Terashima, A., Himeda, C., Kikuchi, Y., and Kimelman, D. (2005). Eomesodermin is a localized maternal determinant required for endoderm induction in zebrafish. Dev. Cell *9*, 523–533.

Bogdanovic, O., Fernandez-Miñán, A., Tena, J.J., de la Calle-Mustienes, E., Hidalgo, C., van Kruysbergen, I., van Heeringen, S.J., Veenstra, G.J., and Gómez-Skarmeta, J.L. (2012). Dynamics of enhancer chromatin signatures

mark the transition from pluripotency to cell specification during embryogenesis. Genome Res. *22*, 2043–2053.

Boldajipour, B., Doitsidou, M., Tarbashevich, K., Laguri, C., Yu, S.R., Ries, J., Dumstrei, K., Thelen, S., Dörries, J., Messerschmidt, E.M., et al. (2011). Cxcl12 evolution–subfunctionalization of a ligand through altered interaction with the chemokine receptor. Development *138*, 2909–2914.

Brown, J.L., Snir, M., Noushmehr, H., Kirby, M., Hong, S.K., Elkahloun, A.G., and Feldman, B. (2008). Transcriptional profiling of endogenous germ layer precursor cells identifies dusp4 as an essential gene in zebrafish endoderm specification. Proc. Natl. Acad. Sci. USA *105*, 12337–12342.

Bruce, A.E., Howley, C., Zhou, Y., Vickers, S.L., Silver, L.M., King, M.L., and Ho, R.K. (2003). The maternally expressed zebrafish T-box gene eomesodermin regulates organizer formation. Development *130*, 5503–5517.

Bruce, A.E., Howley, C., Dixon Fox, M., and Ho, R.K. (2005). T-box gene eomesodermin and the homeobox-containing Mix/Bix gene mtx2 regulate epiboly movements in the zebrafish. Dev. Dyn. *233*, 105–114.

Chang, K.P., Yu, J.S., Chien, K.Y., Lee, C.W., Liang, Y., Liao, C.T., Yen, T.C., Lee, L.Y., Huang, L.L., Liu, S.C., et al. (2011). Identification of PRDX4 and P4HA2 as metastasis-associated proteins in oral cavity squamous cell carcinoma by comparative tissue proteomics of microdissected specimens using iTRAQ technology. J. Proteome Res. *10*, 4935–4947.

Danos, M.C., and Yost, H.J. (1996). Role of notochord in specification of cardiac left-right orientation in zebrafish and Xenopus. Dev. Biol. *177*, 96–103.

Dobrovolskaïa-Zavadskaïa, N. (1927). Sur la mortification spontanée de la queue chez la souris nouveau-née et sur l’existence d’un caractère (facteur) héréditaire “non viable”. C. R. Seanc. Soc. Biol. Fil. *97*, 114–116.

Du, S., Draper, B.W., Mione, M., Moens, C.B., and Bruce, A. (2012). Differential regulation of epiboly initiation and progression by zebrafish Eomesodermin A. Dev. Biol. *362*, 11–23.

Dubrulle, J., Jordan, B.M., Akhmetova, L., Farrell, J.A., Kim, S.H., Solnica-Krezel, L., and Schier, A.F. (2015). Response to Nodal morphogen gradient is determined by the kinetics of target gene induction. eLife *4*, e05042.

Evans, A.L., Faial, T., Gilchrist, M.J., Down, T., Vallier, L., Pedersen, R.A., Wardle, F.C., and Smith, J.C. (2012). Genomic targets of Brachyury (T) in differentiating mouse embryonic stem cells. PLoS ONE *7*, e33346.

Faial, T., Bernardo, A.S., Mendjan, S., Diamanti, E., Ortmann, D., Gentsch, G.E., Mascetti, V.L., Trotter, M.W., Smith, J.C., and Pedersen, R.A. (2015). Brachyury and SMAD signalling collaboratively orchestrate distinct mesoderm and endoderm gene regulatory networks in differentiating human embryonic stem cells. Development *142*, 2121–2135.

Feldman, B., and Stemple, D.L. (2001). Morpholino phenocopies of sqt, oep, and ntl mutations. Genesis *30*, 175–177.

Garnett, A.T., Han, T.M., Gilchrist, M.J., Smith, J.C., Eisen, M.B., Wardle, F.C., and Amacher, S.L. (2009). Identification of direct T-box target genes in the developing zebrafish mesoderm. Development *136*, 749–760.

Gentsch, G.E., Owens, N.D., Martin, S.R., Piccinelli, P., Faial, T., Trotter, M.W., Gilchrist, M.J., and Smith, J.C. (2013). In vivo T-box transcription factor profiling reveals joint regulation of embryonic neuromesodermal bipotency. Cell Rep. *4*, 1185–1196.

Germain, S., Howell, M., Esslemont, G.M., and Hill, C.S. (2000). Homeodomain and winged-helix transcription factors recruit activated Smads to distinct promoter elements via a common Smad interaction motif. Genes Dev. *14*, 435–451.

Giardine, B., Riemer, C., Hardison, R.C., Burhans, R., Elnitski, L., Shah, P., Zhang, Y., Blankenberg, D., Albert, I., Taylor, J., et al. (2005). Galaxy: a platform for interactive large-scale genome analysis. Genome Res. *15*, 1451–1455.

Goecks, J., Nekrutenko, A., and Taylor, J.; Galaxy Team (2010). Galaxy: a comprehensive approach for supporting accessible, reproducible, and transparent computational research in the life sciences. Genome Biol. *11*, R86.

Griffin, K.J., Amacher, S.L., Kimmel, C.B., and Kimelman, D. (1998). Molecular identification of spadetail: regulation of zebrafish trunk and tail mesoderm formation by T-box genes. Development *125*, 3379–3388.

Halpern, M.E., Ho, R.K., Walker, C., and Kimmel, C.B. (1993). Induction of muscle pioneers and floor plate is distinguished by the zebrafish no tail mutation. Cell *75*, 99–111.

Ho, R.K., and Kane, D.A. (1990). Cell-autonomous action of zebrafish spt-1 mutation in specific mesodermal precursors. Nature *348*, 728–730.

Howe, D.G., Bradford, Y.M., Conlin, T., Eagle, A.E., Fashena, D., Frazer, K., Knight, J., Mani, P., Martin, R., Moxon, S.A., et al. (2013). ZFIN, the zebrafish model organism database: increased support for mutants and transgenics. Nucleic Acids Res. *41*, D854–D860.

Huang, W., Sherman, B.T., and Lempicki, R.A. (2009a). Bioinformatics enrichment tools: paths toward the comprehensive functional analysis of large gene lists. Nucleic Acids Res. *37*, 1–13.

Huang, W., Sherman, B.T., and Lempicki, R.A. (2009b). Systematic and integrative analysis of large gene lists using DAVID bioinformatics resources. Nat. Protoc. *4*, 44–57.

Jahangiri, L., Nelson, A.C., and Wardle, F.C. (2012). A cis-regulatory module upstream of deltaC regulated by Ntla and Tbx16 drives expression in the tailbud, presomitic mesoderm and somites. Dev. Biol. *371*, 110–120.

Kikuchi, Y., Trinh, L.A., Reiter, J.F., Alexander, J., Yelon, D., and Stainier, D.Y. (2000). The zebrafish bonnie and clyde gene encodes a Mix family homeodomain protein that regulates the generation of endodermal precursors. Genes Dev. *14*, 1279–1289.

Kikuchi, Y., Agathon, A., Alexander, J., Thisse, C., Waldron, S., Yelon, D., Thisse, B., and Stainier, D.Y. (2001). casanova encodes a novel Sox-related protein necessary and sufficient for early endoderm formation in zebrafish. Genes Dev. *15*, 1493–1505.

Kimelman, D. (2016). Tales of tails (and trunks): forming the posterior body in vertebrate embryos. Curr. Top. Dev. Biol. *116*, 517–536.

Kispert, A., and Hermann, B.G. (1993). The Brachyury gene encodes a novel DNA binding protein. EMBO J. *12*, 4898–4899.

Langmead, B., Trapnell, C., Pop, M., and Salzberg, S.L. (2009). Ultrafast and memory-efficient alignment of short DNA sequences to the human genome. Genome Biol. *10*, R25.

Leichsenring, M., Maes, J., Mössner, R., Driever, W., and Onichtchouk, D. (2013). Pou5f1 transcription factor controls zygotic gene activation in vertebrates. Science *341*, 1005–1009.

Lolas, M., Valenzuela, P.D., Tjian, R., and Liu, Z. (2014). Charting Brachyury-mediated developmental pathways during early mouse embryogenesis. Proc. Natl. Acad. Sci. USA *111*, 4478–4483.

Lunde, K., Belting, H.G., and Driever, W. (2004). Zebrafish pou5f1/pou2, homolog of mammalian Oct4, functions in the endoderm specification cascade. Curr. Biol. *14*, 48–55.

Martin, B.L., and Kimelman, D. (2008). Regulation of canonical Wnt signaling by Brachyury is essential for posterior mesoderm formation. Dev. Cell *15*, 121–133.

Messenger, N.J., Kabitschke, C., Andrews, R., Grimmer, D., Núñez Miguel, R., Blundell, T.L., Smith, J.C., and Wardle, F.C. (2005). Functional specificity of the Xenopus T-domain protein Brachyury is conferred by its ability to interact with Smad1. Dev. Cell *8*, 599–610.

Mizoguchi, T., Verkade, H., Heath, J.K., Kuroiwa, A., and Kikuchi, Y. (2008). Sdf1/Cxcr4 signaling controls the dorsal migration of endodermal cells during zebrafish gastrulation. Development *135*, 2521–2529.

Mootha, V.K., Lindgren, C.M., Eriksson, K.F., Subramanian, A., Sihag, S., Lehar, J., Puigserver, P., Carlsson, E., Ridderstråle, M., Laurila, E., et al. (2003). PGC-1alpha-responsive genes involved in oxidative phosphorylation are coordinately downregulated in human diabetes. Nat. Genet. *34*, 267–273.

Morley, R.H., Lachani, K., Keefe, D., Gilchrist, M.J., Flicek, P., Smith, J.C., and Wardle, F.C. (2009). A gene regulatory network directed by zebrafish No tail accounts for its roles in mesoderm formation. Proc. Natl. Acad. Sci. USA *106*, 3829–3834.

Mueller, R.L., Huang, C., and Ho, R.K. (2010). Spatio-temporal regulation of Wnt and retinoic acid signaling by tbx16/spadetail during zebrafish mesoderm differentiation. BMC Genomics *11*, 492.

Naiche, L.A., Harrelson, Z., Kelly, R.G., and Papaioannou, V.E. (2005). T-box genes in vertebrate development. Annu. Rev. Genet. *39*, 219–239.

Nair, S., and Schilling, T.F. (2008). Chemokine signaling controls endodermal migration during zebrafish gastrulation. Science *322*, 89–92.

Nelson, A.C., Cutty, S.J., Niini, M., Stemple, D.L., Flicek, P., Houart, C., Bruce, A.E., and Wardle, F.C. (2014). Global identification of Smad2 and Eomesodermin targets in zebrafish identifies a conserved transcriptional network in mesendoderm and a novel role for Eomesodermin in repression of ectodermal gene expression. BMC Biol. *12*, 81.

Osborne, N., Brand-Arzamendi, K., Ober, E.A., Jin, S.W., Verkade, H., Holtzman, N.G., Yelon, D., and Stainier, D.Y. (2008). The spinster homolog, two of hearts, is required for sphingosine 1-phosphate signaling in zebrafish. Curr. Biol. *18*, 1882–1888.

Pavesi, G., Mereghetti, P., Zambelli, F., Stefani, M., Mauri, G., and Pesole, G. (2006). MoD tools: regulatory motif discovery in nucleotide sequences from co-regulated or homologous genes. Nucleic Acids Res. *34*, W566–W570.

Poulain, M., Fürthauer, M., Thisse, B., Thisse, C., and Lepage, T. (2006). Zebrafish endoderm formation is regulated by combinatorial Nodal, FGF and BMP signalling. Development *133*, 2189–2200.

Reim, G., Mizoguchi, T., Stainier, D.Y., Kikuchi, Y., and Brand, M. (2004). The POU domain protein spg (pou2/Oct4) is essential for endoderm formation in cooperation with the HMG domain protein casanova. Dev. Cell *6*, 91–101.

Rodaway, A., Takeda, H., Koshida, S., Broadbent, J., Price, B., Smith, J.C., Patient, R., and Holder, N. (1999). Induction of the mesendoderm in the zebrafish germ ring by yolk cell-derived TGF-beta family signals and discrimination of mesoderm and endoderm by FGF. Development *126*, 3067–3078.

Roehl, H., and Nüsslein-Volhard, C. (2001). Zebrafish pea3 and erm are general targets of FGF8 signaling. Curr. Biol. *11*, 503–507.

Sanges, R., Cordero, F., and Calogero, R.A. (2007). oneChannelGUI: a graphical interface to Bioconductor tools, designed for life scientists who are not familiar with R language. Bioinformatics *23*, 3406–3408.

Schier, A.F. (2009). Nodal morphogens. Cold Spring Harb. Perspect. Biol. *1*, a003459.

Schulte-Merker, S., Ho, R.K., Herrmann, B.G., and Nüsslein-Volhard, C. (1992). The protein product of the zebrafish homologue of the mouse T gene is expressed in nuclei of the germ ring and the notochord of the early embryo. Development *116*, 1021–1032.

Schulte-Merker, S., van Eeden, F.J., Halpern, M.E., Kimmel, C.B., and Nüsslein-Volhard, C. (1994). no tail (ntl) is the zebrafish homologue of the mouse T (Brachyury) gene. Development *120*, 1009–1015.

Seebald, J.L., and Szeto, D.P. (2011). Zebrafish eve1 regulates the lateral and ventral fates of mesodermal progenitor cells at the onset of gastrulation. Dev. Biol. *349*, 78–89.

Shin, C.H., Chung, W.S., Hong, S.K., Ober, E.A., Verkade, H., Field, H.A., Huisken, J., and Stainier, D.Y. (2008). Multiple roles for Med12 in vertebrate endoderm development. Dev. Biol. *317*, 467–479.

Showell, C., Binder, O., and Conlon, F.L. (2004). T-box genes in early embryogenesis. Dev. Dyn. *229*, 201–218.

Slagle, C.E., Aoki, T., and Burdine, R.D. (2011). Nodal-dependent mesendoderm specification requires the combinatorial activities of FoxH1 and Eomesodermin. PLoS Genet. *7*, e1002072.

Stückemann, T., Wegleiter, T., Stefan, E., Nägele, O., Tarbashevich, K., Böck, G., Raz, E., and Aanstad, P. (2012). Zebrafish Cxcr4a determines the proliferative response to Hedgehog signalling. Development *139*, 2711–2720.

Subramanian, A., Tamayo, P., Mootha, V.K., Mukherjee, S., Ebert, B.L., Gillette, M.A., Paulovich, A., Pomeroy, S.L., Golub, T.R., Lander, E.S., and Mesirov, J.P. (2005). Gene set enrichment analysis: a knowledge-based approach for interpreting genome-wide expression profiles. Proc. Natl. Acad. Sci. USA *102*, 15545–15550.

van Boxtel, A.L., Chesebro, J.E., Heliot, C., Ramel, M.C., Stone, R.K., and Hill, C.S. (2015). A temporal window for signal activation dictates the dimensions of a Nodal signaling domain. Dev. Cell *35*, 175–185.

Warga, R.M., Mueller, R.L., Ho, R.K., and Kane, D.A. (2013). Zebrafish Tbx16 regulates intermediate mesoderm cell fate by attenuating Fgf activity. Dev. Biol. *383*, 75–89.

Westerfield, M. (2000). The Zebrafish Book. A Guide for the Laboratory Use of Zebrafish (Danio rerio), Fourth Edition (Eugene: University of Oregon Press).

Xu, C., Fan, Z.P., Müller, P., Fogley, R., DiBiase, A., Trompouki, E., Unternaehrer, J., Xiong, F., Torregroza, I., Evans, T., et al. (2012). Nanog-like regulates endoderm formation through the Mxtx2-Nodal pathway. Dev. Cell *22*, 625–638.

Zhang, J., Houston, D.W., King, M.L., Payne, C., Wylie, C., and Heasman, J. (1998). The role of maternal VegT in establishing the primary germ layers in Xenopus embryos. Cell *94*, 515–524.

Zhang, Y., Liu, T., Meyer, C.A., Eeckhoute, J., Johnson, D.S., Bernstein, B.E., Nusbaum, C., Myers, R.M., Brown, M., Li, W., and Liu, X.S. (2008). Model-based analysis of ChIP-seq (MACS). Genome Biol. *9*, R137.

Zhang, H., Fraser, S.T., Papazoglu, C., Hoatlin, M.E., and Baron, M.H. (2009). Transcriptional activation by the Mixl1 homeodomain protein in differentiating mouse embryonic stem cells. Stem Cells *27*, 2884–2895.

Cell Reports, Volume *19*

Supplemental Information

In Vivo Regulation of the Zebrafish Endoderm Progenitor Niche by T-Box Transcription Factors

Andrew C. Nelson, Stephen J. Cutty, Saule N. Gasiunas, Isabella Deplae, Derek L. Stemple, and Fiona C. Wardle

Supplemental Figures

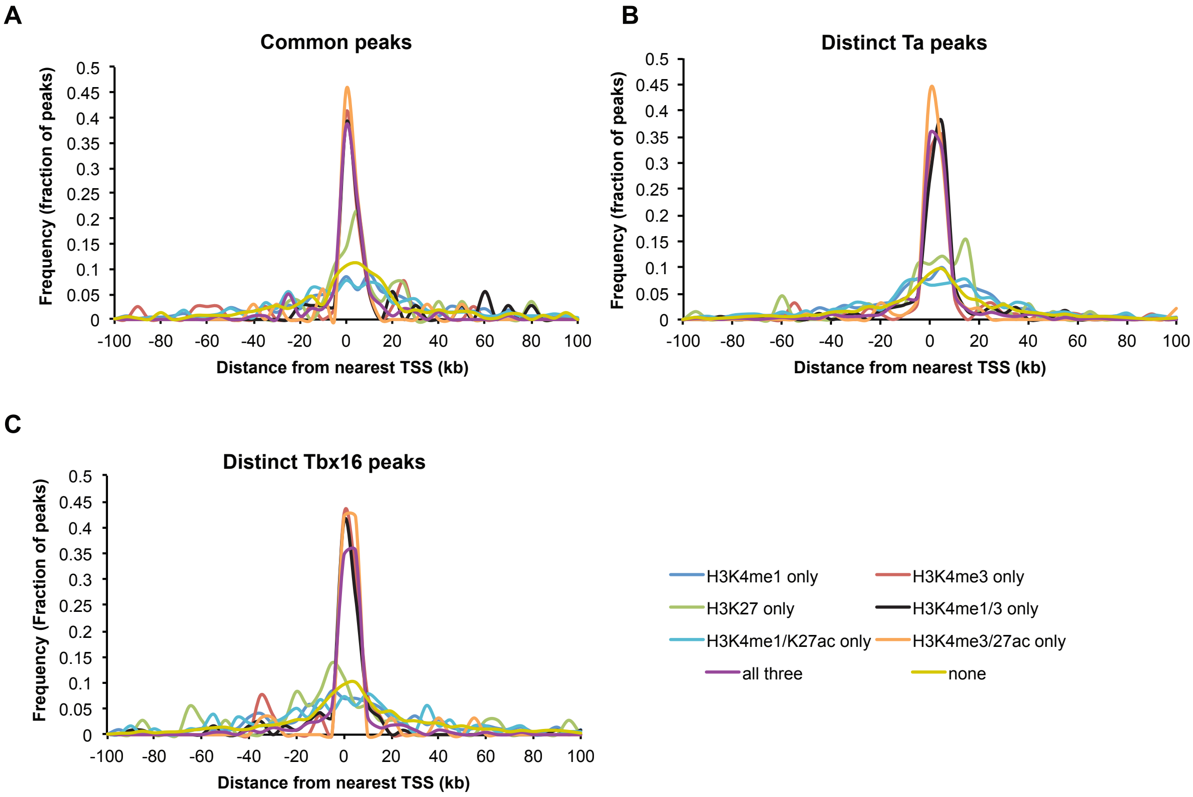

Figure S1 (relates to Figure 1). Distribution of Ta and Tbx16 peaks overlapping histone marks relative to the nearest TSS ±100kb.

(A-C) Graphs indicating the distribution of Ta/Tbx16 common and distinct peaks at each combination of histone marks as defined in Figure 1H. Though nearest TSS ±100kb were annotated the majority of peaks are markedly closer. As expected, Ta and Tbx16 ChIP-seq peaks overlapping H3K4me3 (putative promoters) tend to occur in close proximity to TSSs, while those overlapping H3K4me1 (putative enhancers) are more distant.

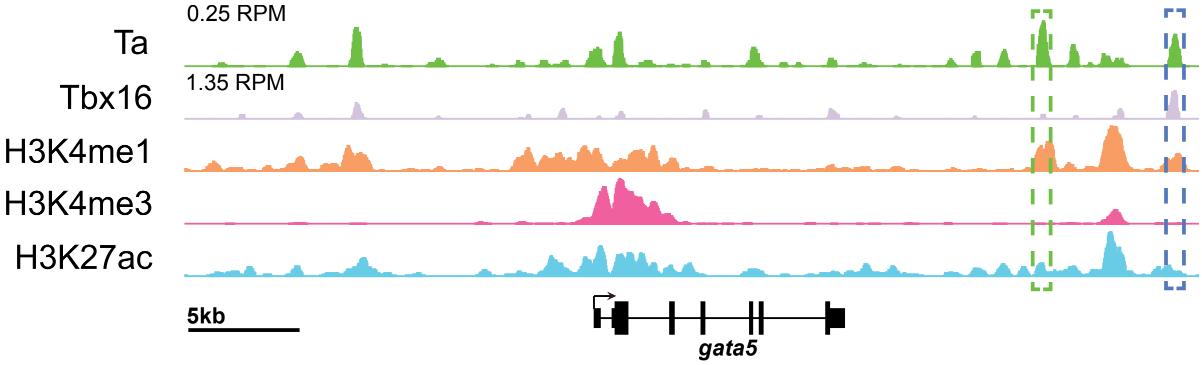

Figure S2 (relates to Figure 4). Endodermal regulator *gata5* exhibits proximal binding of Ta and Tbx16. Stage-matched Ta, Tbx16, H3K4me1, H3K4me3 and H3K27ac and ChIP-seq at the *gata5* locus. Peak heights in reads per million (RPM) are indicated. Common (blue box) and Distinct Ta (green box) peaks called as significant are indicated.

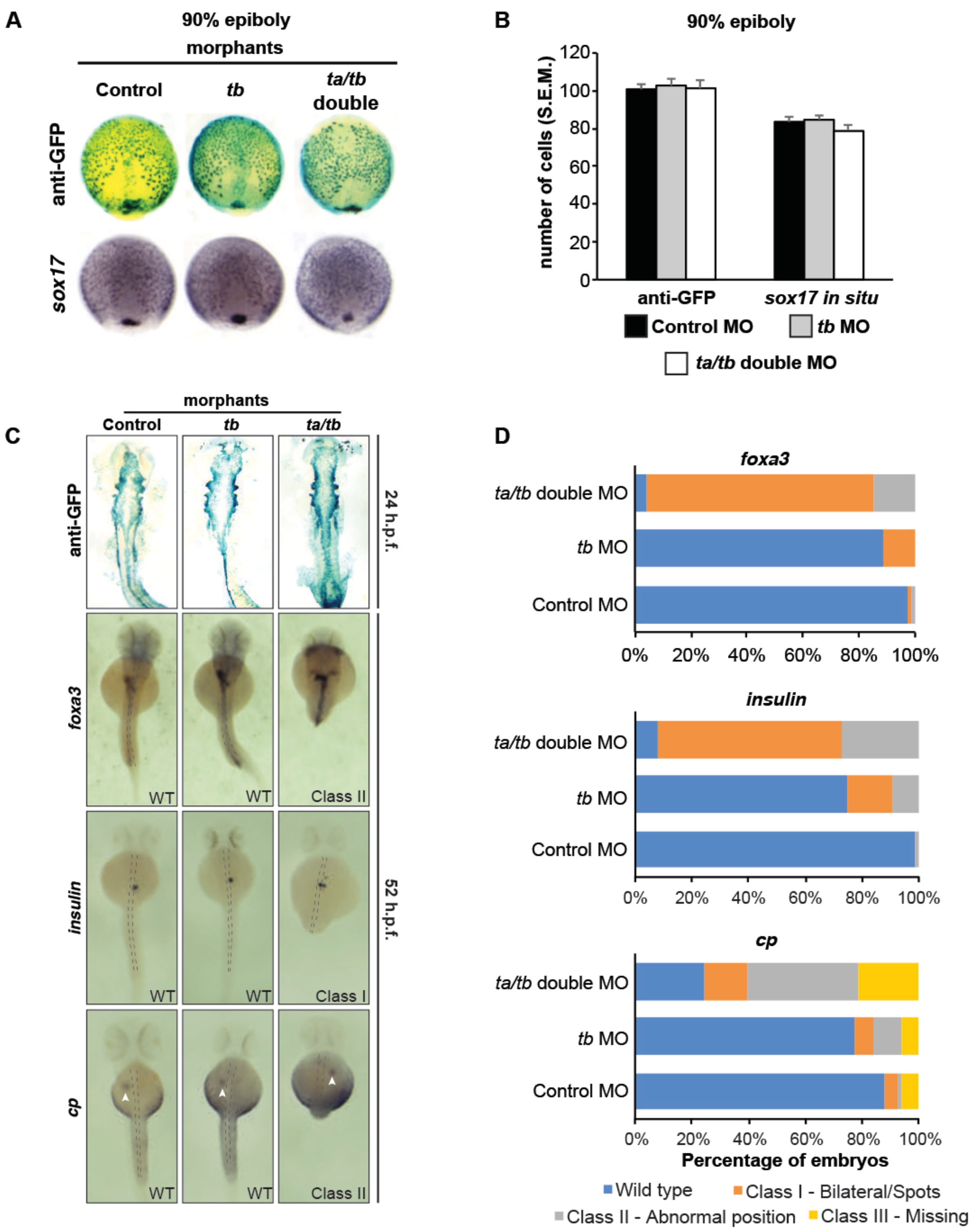

Figure S3 (relates to Figures 4 and 5). Morpholino knockdown of Brachyury homologue Tb does not substantially enhance the Ta-mediated effects on endoderm progenitor numbers at 90% epiboly, but does enhance downstream laterality defects. (A) Immunological and WISH analysis of a *sox17:eGFP* transgene and endogenous *sox17* expression at 90% epiboly (9 h.p.f.) in single and double morphants as indicated. (B) Quantification of cell numbers identified by immunostaining and WISH in panel A. Cell numbers are representative of at least 20 embryos per condition. (C) Anti-GFP immunostaining of flat-mounted *sox17:eGFP* embryos at 24 h.p.f. and WISH analysis of broad marker of endodermal organs *foxa3*, pancreas marker *ins* and liver marker *cp* at 52-56 h.p.f. in single and double morphants. Examples of phenotypic classifications are indicated. WT = wild type. Dashed lines indicate the midline. (D) Phenotypic quantification of morphant embryos from panel C experiment. Each group is representative of at least 30 embryos.

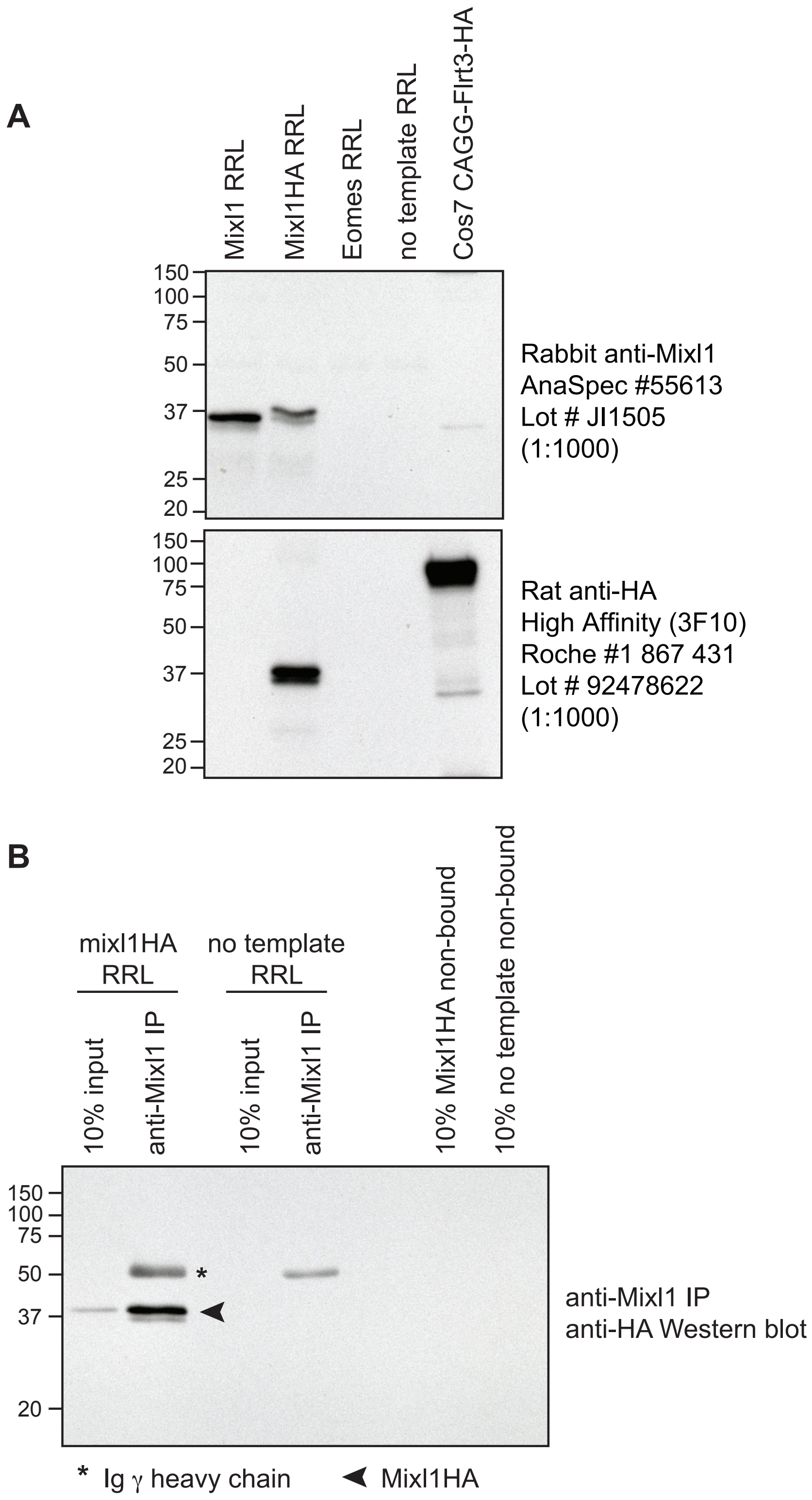

Figure S4 (relates to Figure 6). The rabbit polyclonal antibody used for anti-Mixl1 ChIP-seq recognizes Mixl1 protein in both Western blot and immunoprecipitation assays. (A) Western blot demonstrating that the anti-Mixl1 rabbit polyclonal antibody cleanly recognizes HA-tagged and untagged Mixl1 protein produced in rabbit reticulocyte lysate (RRL), whereas a rat anti-HA monoclonal antibody only recognizes HA-tagged proteins. Molecular weight standards in kDa are indicated. (B) Immunoprecipitation of Mixl1HA protein using anti-Mixl1 rabbit polyclonal antibody followed by anti-HA Western blot demonstrates that Mixl1 protein was efficiently captured.

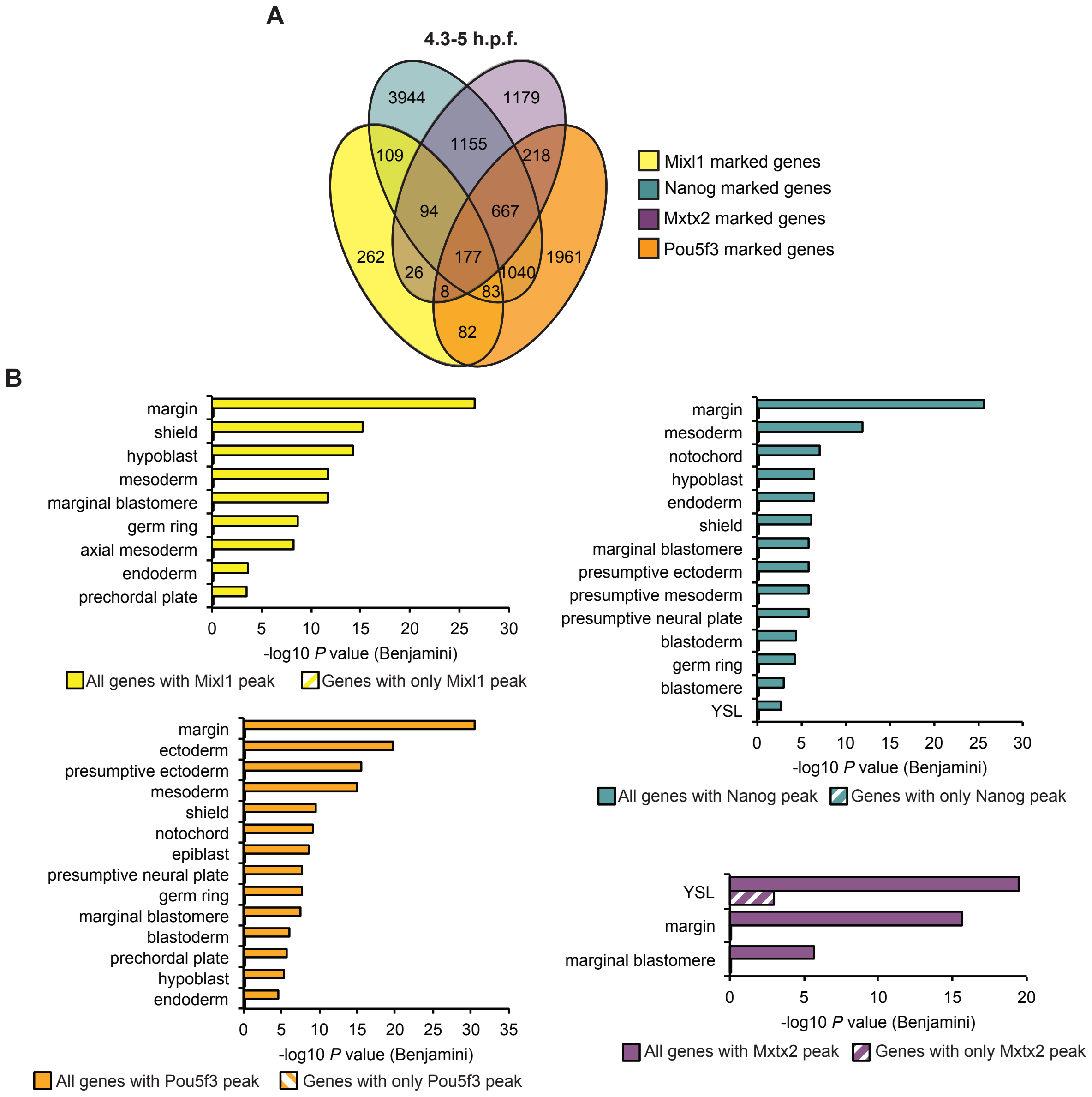

Figure S5 (relates to Figure 7). Common putative targets of Mixl1/Nanog/Mxtx2/Pou5f3 correlate with their expression patterns in the early embryo.

(A) Venn diagram indicating the number of genes with binding of Mixl1, Nanog, Mxtx2 or Pou5f3 (nearest TSS ±100kb; different to Figure 7B which indicated the overlap between ChIP-seq peaks – this Venn diagram indicates number of genes marked by ChIP-seq peaks regardless of whether the peaks overlap). Note the lower proportion of genes marked by a single factor, compared with ChIP-seq peaks unique to a single factor. This is due to many genes displaying binding for multiple factors at different CRMs.

(B) Anatomical enrichment analysis of total genes bound by each factor (nearest TSS ±100kb), or of genes bound by only each individual factor. Analysis was performed relative to anatomical expression patterns recorded in the ZFIN database using DAVID. The reported terms are for cell types/tissues where the factors are reported to be expressed, or which are known to be directly altered in loss-of-function mutants. Note that with the exception of Mxtx2, genes showing binding for individual factors alone are poorly enriched for anatomical terms. Mxtx2 alone shows binding at YSL genes as expected for this YSL expressed factor. This analysis suggests combinations of these four factors regulate their known cell type-specific functions. Tables of significant functional and anatomical terms are in Data S5.

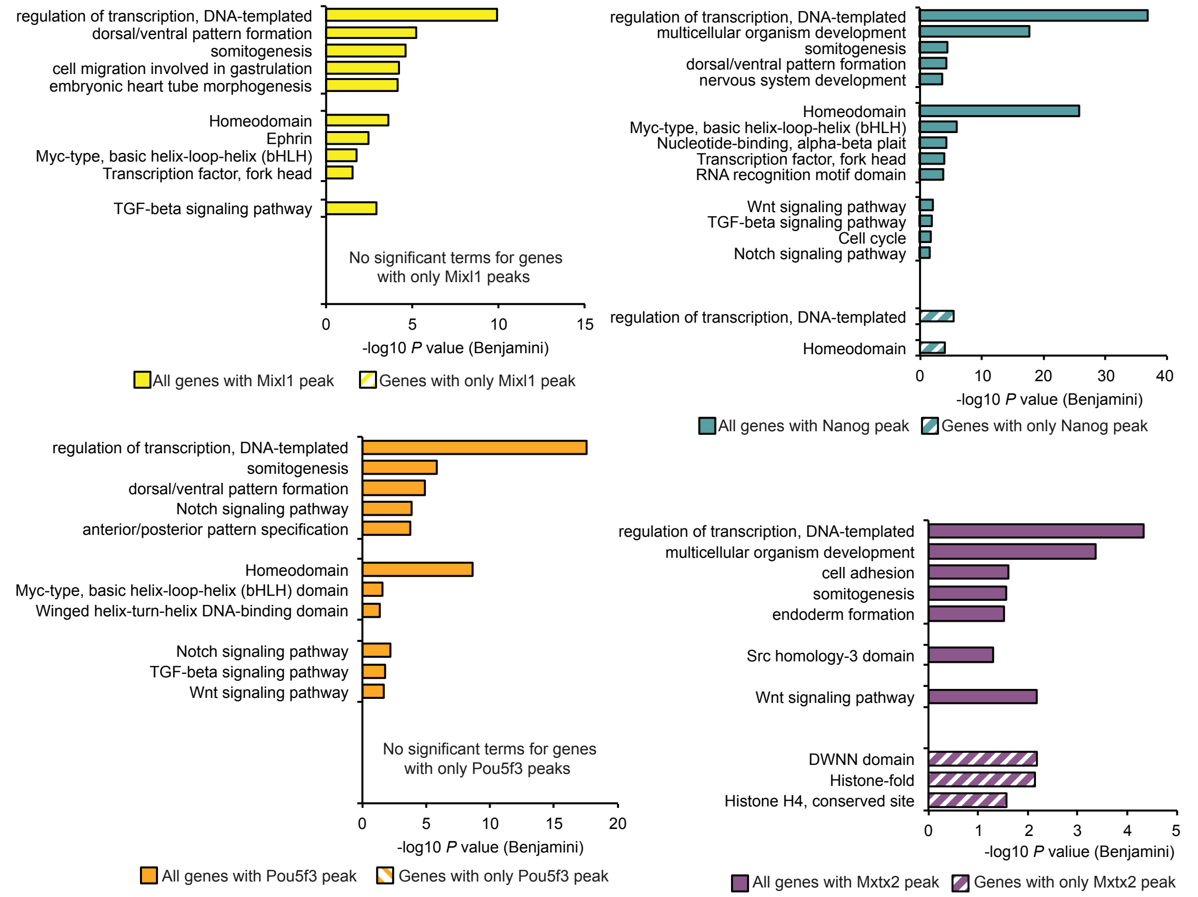

Figure S6 (relates to Figure 7). Genes with binding of individual factors show poor enrichment for functional annotation terms.

Reported for each of Mixl1, Nanog, Pou5f3 and Mxtx2 are the five most significant (fewer if fewer significant terms) non-redundant Gene Ontology Biological Process, InterPro and KEGG Pathway terms significantly enriched amongst genes with ChIP-seq peaks (nearest TSS ±100kb). Note that with the exception of Mxtx2, genes showing binding for individual factors alone are poorly enriched for functional terms. This analysis strongly suggests that these factors act in combination to regulate their target genes in early development. Tables of significant functional and anatomical terms are in Data S5.

Factor	Stage	Sample	Read length	Total number of reads	Final number of mapped reads	Percentage of total
**Ta**	75-85%	ChIP1	38	11,396,372	2,779,764	24.4
	epiboly	ChIP2	38	25,406,256	6,021,884	23.7
		Input	38	10,690,296	2,692,914	25.2
**Tbx16**	75-85%	ChIP1	38	31,622,398	6,957,583	22.0
	epiboly	ChIP2	38	29,880,883	7,173,663	24.0
		Input	38	12,412,184	4,850,591	39.1
**Mixl1**	30-50%	ChIP1	63	29,671,488	5,913,904	19.9
	epiboly	ChIP2	63	239,043,044	7,984,896	3.3
		Input	63	107,848,351	10,018,032	9.3

Table S1 (relates to Figures 1-3 and 6-7). Summary of reads and mapping statistics for Ta, Tbx16 and Mixl1 ChIP-seq experiments.

Supplemental Data

Data S1. Ta and Tbx16 ChIP-seq coordinates. The nearest TSS ±100kb of each peak and associated gene descriptions and distance from the peak are provided, as well as whether each peak overlaps H3K4me1, H3K4me3 and H3K27ac ChIP-seq peaks from the Bogdanovic *et al.* (2012) dataset (relates to Figure 1).

Data S2. Anatomical and functional annotation analysis of genes marked by common and distinct Ta and Tbx16 peaks (relates to Figure 2).

Data S3. Overlap of Ta and Tbx16 ChIP-seq peaks with Eomesa ChIP-seq peaks, plus Mixl1 ChIP-seq peak overlaps with Eomesa and Smad2 ChIP-seq peaks (relates to Figures 3 and 6). The nearest TSS ±100kb of each peak and associated gene descriptions are provided.

Data S4. Microarray data analysis of wild type vs. *ta;tbx16* double morphants at 90% epiboly (9 h.p.f.; relates to Figure 4).

Data S5. Anatomical annotation analysis of genes marked by Mixl1 ChIP-seq peaks overlapping Smad2 and/or Eomesa ChIP-seq peaks (relates to Figure 6). Also anatomical and functional annotation analysis of genes marked by Mixl1, Nanog, Mxtx2 and/or Pou5f3 related to Figure S5-6.

Data S6. Microarray data analysis of wild type vs. *ta;tbx16* double morphants at shield stage (6 h.p.f.), and list of 20 genes from leading edge analysis highlighted in Figure 6H.

Data S7. Tables of genomic coordinates occupied by two or more of Eomesa, Mixl1, Mxtx2, Nanog, Pou5f3 and Smad2 (relates to Figure 7). The nearest TSS ±100kb of each peak and associated gene descriptions are provided.

Supplemental Experimental Procedures

RNA extraction, cDNA synthesis and qRT-PCR

Extraction of RNA, synthesis of cDNA and qPCR was performed as reported previously (Cutty et al., 2012). The primers used for qRT-PCR analysis are listed below:

gene	forward	reverse	reference
sox32	CCGTACATGCAAGAAGCAGA	ATCAAAGGTGGCATTTGGAG	
mixl1	ACCCAAAGGAACACGAACAG	AACAGGCGATGTGTTTAGGG	
gata5	AGAGACGGAACCGGACACTA	TCTCCTCCACAGTGTTGTCG	

ChIP-qPCR analysis

ChIP-qPCR was performed on approximately 300 to 500 embryos at the indicated stages using 1ul anti-Mixl1 antibody (Anaspec 55613, lot number JI1505), 3ul anti-Ta antibody, 3ul anti-Tbx16 antibody, 3ul anti-Eomesa antibody, 0.5ug anti-Smad2 antibody (clone 31H15L4; Thermo Fisher Scientific) and normal rabbit IgG as described (Garnett et al., 2009; Jahangiri et al., 2012; Morley et al., 2009; Nelson et al., 2014). qPCR was carried out on a Mx3005P (Agilent Technologies, Santa Clara, CA, USA) using SYBR Green 1 Master kit (Roche Basel, Switzerland) according to the manufacturer's instructions. Values for each condition were calculated as a percentage of input and are presented as fold enrichment relative to the IgG control. Primer sequences were as follows:

gene	forward	reverse	reference
col8a1a	ATGGTGTCTTTGCATGCGTG	GCCTCTAATGGACGTCTGCT	
p4ha2	ACCCGACTGCTTCCTGTTAA	GCTGTTCTGAAAAGCCGACT	
etv4	GTGGGAGTTGACTGAATGGC	AGGTGGGAGCTGGACTTTAC	
eve1	CCTCCAGAAAAGCTTTCTTCT CTAT	CAGAGGGAGGTGTTAAATTGTC TT	Morley et al., 2009
itga6a	TCACATTCTTCCGGCTTCCT	TGGCAGCTCGTATCTCTCTG	
dmd	TCGCATTGTCGAGAGTTCCA	CCCTCGCTGTCACACATAGT	
tbx16 peak	CAGAAAGCAAACCCCTCGAG	GCATGAATAGAGAGCCTGCTG	
cxcl12b	TCTGACACCTCACACATGCA	GCAGATTTGGGAGTTCAGCC	Nelson et al., 2014 (Eomes peak 3484)
mixl1 peak	ATTAAACTCGCACACGAACCT T	CCTGAAATGAGTGGCTTTTCTT	Nelson et al., 2014 (Common Peak 187)
foxh1	TCTGCAGTCAAGGTGATGGT	CAGTCTGAGGGGTTGAGGAG	
foxa2	CCTGTGGCCCAATTGAAGAG	CCTGCGAGTGACTGCAATAC	
sox32 peak 1	GCCCGTGTGTAGTGAGAGAT	AACTGCGCGACTATTTCTGA	
sox32 peak 2	AATTGTGCGAAATGGCCAC	ACTACAGCACAGTCACACGT	
gata5 peak	TAAGCCGTGTAACCATGCAG	TGATGTTGCGACCTGTGAAC	
Negative region (*rhod*)	GACTCCACACAATCTGCAACA T	ACCACCTACGCTAAAGAAACCA	Morley et al., 2009

Whole-mount immunostaining and *in situ* hybridization

Embryos fixed in 4% PFA were washed (wash buffer: Phosphate Buffered Saline (PBS);1%DMSO; 0.1%Triton X-100), blocked (2% goat serum;1% Bovine Serum Albumin (BSA) in wash buffer) for 1 hour at room temperature, rewashed and incubated with mouse monoclonal anti-GFP 3E6 IgG2a in wash buffer (A11120, Molecular Probes, 1:300 in block) overnight at 4°C. Embryos were rewashed, reblocked and incubated with Goat anti-mouse HRP- conjugate in wash buffer (Thermo Scientific diluted to 1μg/ml in block) for four hours at room temperature. Embryos were rewashed, developed using HistoGreen (Linaris) according to the manufacturer's instructions, rewashed, processed through a glycerol gradient and imaged on a Leica MZ16F dissecting

microscope. 24 h.p.f. were permeabilized with ice cold acetone for 7 minutes at -20°C prior to primary antibody incubation.

In situ hybridization was performed as described (Jowett and Lettice, 1994). Dioxygenin-labelled RNA probes for *sox32* (Alexander et al., 1999), *sox17* (Alexander and Stainier, 1999), *foxa3* (Odenthal and Nusslein-Volhard, 1998), *ins* (Milewski et al., 1998) and *cp* (Korzh et al., 2001) were produced as previously described.

Cell counting
Photographs of each embryo were taken from a dorsal perspective and using PowerPoint, sharpness was increased to 100% on each photograph to enhance contrast between stained cells and background. A grid was overlaid to ensure the orientation of the embryo was the same in each photograph, with the embryonic midline central on the grid and the dorsal forerunner cells located in the bottom central square, and the cells were manually counted.

Tb morpholino injection
0.5 pmol of *tb* translation blocking morpholino (GACAGTCACCGGAGTCCATTCTATC; Martin and Kimelman, 2008) was injected with 0.25 pmol of the p53 translation blocking morpholino (GCGCCATTGCTTTGCAAGAATTG; Langheinrich et al., 2002) into one-cell stage embryos.

***In vitro* protein production and Western blot analyses**
Mixl1 expression constructs were pCS2+ *mixl1*, pCS2+ *mix1HA* and pCS2+ *Eomesodermin*. Plasmids were linearized using NotI. Linearized constructs were transcribed with SP6 polymerase to generate capped mRNA according to standard protocols. Capped mRNA for each protein was used to synthesize protein in rabbit reticulocyte lysate according to manufacturer's instructions (Promega). Reticulocyte lysates were subjected to SDS/PAGE on a 10% gel, then blotted to PVDF membrane (GE Healthcare) according to standard protocols. Membranes were blocked for 1 hour at room temperature in 5% skimmed milk powder in Tris buffered saline/0.1% Tween 20 (TBST), then incubated for 16 hours at 4°C in antibodies diluted in block as indicated in Figure S2. Blots were then washed extensively in TBST, incubated for 1 hour in either anti-rat or anti-rabbit HRP-conjugated antibodies (GE Healthcare), washed extensively in TBST before detection with Amersham ECL Prime Western Blotting Detection Reagent (GE Healthcare).

Immunoprecipitation
1μl of *in vitro* translated Mixl1HA protein was incubated for 16 hours with 1μg of anti-Mixl1 rabbit polyclonal antibody (Anaspec 55613, lot number JI1505) and 15μl BSA-blocked Dynabeads Protein G (ThermoFisher) at 4 ^{o}C followed by extensive washing with RIPA buffer. Dynabeads were then boiled in 1x SDS sample buffer and eluate subjected to anti-HA Western blot analysis.

Supplemental References

Alexander, J., Rothenberg, M., Henry, G.L., and Stainier, D.Y. (1999). casanova plays an early and essential role in endoderm formation in zebrafish. Dev Biol *215*, 343-357.
Alexander, J., and Stainier, D.Y. (1999). A molecular pathway leading to endoderm formation in zebrafish. Curr Biol *9*, 1147-1157.
Cutty, S.J., Fior, R., Henriques, P.M., Saude, L., and Wardle, F.C. (2012). Identification and expression analysis of two novel members of the Mesp family in zebrafish. Int J Dev Biol *56*, 285-294.
Korzh, S., Emelyanov, A., and Korzh, V. (2001). Developmental analysis of ceruloplasmin gene and liver formation in zebrafish. Mech Dev *103*, 137-139.
Langheinrich, U., Hennen, E., Stott, G., and Vacun, G. (2002). Zebrafish as a model organism for the identification and characterization of drugs and genes affecting p53 signaling. Curr Biol *12*, 2023-2028.
Milewski, W.M., Duguay, S.J., Chan, S.J., and Steiner, D.F. (1998). Conservation of PDX-1 structure, function, and expression in zebrafish. Endocrinology *139*, 1440-1449.
Odenthal, J., and Nusslein-Volhard, C. (1998). fork head domain genes in zebrafish. Dev Genes Evol *208*, 245-258.
